# ﻿Revision of genus *Zele* Curtis (Hymenoptera, Braconidae, Euphorinae) from China, with description of nineteen new species

**DOI:** 10.3897/zookeys.1248.158182

**Published:** 2025-08-05

**Authors:** Yu Fang, Cornelis van Achterberg, Pu Tang, Xue-xin Chen

**Affiliations:** 1 State Key Lab of Rice Biology and Breeding, Zhejiang University, Hangzhou 310058, China Zhejiang University Hangzhou China; 2 Ministry of Agriculture and Rural Affairs, Key Lab of Molecular Biology of Crop Pathogens and Insects, Zhejiang University, Hangzhou 310058, China Zhejiang University Hangzhou China; 3 Zhejiang Provincial Key Laboratory of Biology and Ecological Regulation of Crop Pathogens and Insects, Zhejiang University, Hangzhou 310058, China Zhejiang University Hangzhou China; 4 Institute of Insect Sciences, College of Agriculture and Biotechnology, Zhejiang University, Hangzhou 310058, China Zhejiang University Hangzhou China

**Keywords:** DNA barcodes, key, Meteorini, new species, phylogeny, reinstated species

## Abstract

*Zele* Curtis is a braconid parasitoid wasp genus within the subfamily Euphorinae (Hymenoptera, Braconidae), consisting of only 30 species worldwide. The Chinese species of the genus *Zele* are revised and 29 species are now recognised, including 19 species new to science: *Z.aquilus* Fang, van Achterberg & Chen, **sp. nov.**, *Z.carinatus* Fang, van Achterberg & Chen, **sp. nov.**, *Z.confusus* Fang, van Achterberg & Chen, **sp. nov.**, *Z.cristatus* Fang, van Achterberg & Chen, **sp. nov.**, *Z.curvatus* Fang, van Achterberg & Chen, **sp. nov.**, *Z.curvinervis* Fang, van Achterberg & Chen, **sp. nov.**, *Z.densipunctatus* Fang, van Achterberg & Chen, **sp. nov.**, *Z.extensus* Fang, van Achterberg & Chen, **sp. nov.**, *Z.fulgidus* Fang, van Achterberg & Chen, **sp. nov.**, *Z.fuscatus* Fang, van Achterberg & Chen, **sp. nov.**, *Z.impolitus* Fang, van Achterberg & Chen, **sp. nov.**, *Z.inclinator* Fang, van Achterberg & Chen, **sp. nov.**, *Z.irregularis* Fang, van Achterberg & Chen, **sp. nov.**, *Z.petiolatus* Fang, van Achterberg & Chen, **sp. nov.**, *Z.rugulosus* Fang, van Achterberg & Chen, **sp. nov.**, *Z.sculpticoxis* Fang, van Achterberg & Chen, **sp. nov.**, *Z.shaanxiensis* Fang, van Achterberg & Chen, **sp. nov.**, *Z.syntomus* Fang, van Achterberg & Chen, **sp. nov.**, and *Z.vacatus* Fang, van Achterberg & Chen, **sp. nov.** 31 barcode region sequences of mitochondrial cytochrome c oxidase I (COI) from the genus *Zele* were obtained and combined with 102 sequences from BOLD. They were used to validate new species and to get an indication of similarity among species. Three species are reinstated: *Zeleperonatus* (Shestakov, 1940), *Z.romani* (Fahringer, 1929), and *Z.rufulus* (Thomson, 1895). In addition, an identification key for the *Zele* species recorded in China (plus one expected species) is provided.

## ﻿Introduction

The genus *Zele* Curtis, 1832, is currently placed in the subfamily Euphorinae (Stigenberg et al. 2015; Chen and van Achterberg 2019), but it had previously been classified into various subfamilies, including Meteorinae (Muesebeck 1923), Euphorinae (Muesebeck 1936), Macrocentrinae (Nixon 1938; Eady and Clark 1964; Čapek 1969), Helconinae (Watanabe 1969), Zelinae (Tobias 1967, 1971; Mason 1973; van Achterberg 1976) and Zeleinae (Marsh 1979). In Muesebeck (1923), *Zemiotes* Foerster, 1863 (now considered as a junior synonym of *Zele*) was treated as a junior synonym of *Meteorus* Haliday, 1835, that had been classified the subfamily Meteorinae. However, by 1936, Muesebeck revised this position, recognising *Zemiotes* as a synonym of *Meteorus*, and placed the latter genus in the Euphorinae (Muesebeck 1936). Watanabe (1969) suggested placing *Zele* in the tribe Zelini within the subfamily Helconinae. However, Čapek (1969) included *Zele* of the subfamily Macrocentrinae based on studies of the head structure of the last instar larva, aligning with the views of Nixon (1938) and Eady and Clark (1964). Later, Čapek (1970) included *Zele* (now known as *Homolobus* Foerster, 1863) in the status of a tribe Zelini of the Macrocentrinae. Significant contributions to the genus *Zele* were made by van Achterberg (1976, 1979), noting the distinct differences in morphology and host range between *Zele* and Helconinae, initially suggesting that *Zele* should be included in a separate subfamily Zelinae, as earlier proposed by Tobias (1967, 1971).

Mason (1973) accorded generic rank to the genus *Zemiotes* and five of the species that Muesebeck (1923) had treated as *Meteorus* were transferred to *Zemiotes*. Mason advocated that *Zemiotes* should be transferred to the “Zelini” (equivalent to the group now recognised as Homolobinae). Marsh (1979) followed Mason (1973) and classified *Zemiotes* “as a distinct genus related to *Zele*.” Marsh included seven species in *Zemiotes* and classified the genus as a member of the subfamily “Zeleinae,” noting that “the subfamily name correctly spelled “Zelinae” is preoccupied in the Hemiptera. Therefore, Marsh advocated for spelling the braconid subfamily name as “Zeleinae” based on a precedent set by Opinion 140 of the International Commission of Zoological Nomenclature, 1943. “Zeleinae” as discussed by Mason (1973), Marsh (1979), and Shaw (1985) is similar to the current usage of the Homolobinae. In 1979, van Achterberg fundamentally changed the status of *Zele*, making *Zemiotes* a synonym of *Zele*, and transferred *Zele* to the tribe Meteorini within the subfamily Euphorinae. In his revision he also included a new key to *Zele*, redescribed the known species, and identified and documented four new species (van Achterberg 1979). Shaw (1985) elevated meteorines to subfamily status, Meteorinae, and treated *Meteorus* (Meteorinae) as the sister-group of the Euphorinae (s. str.). Shaw (1985) followed Mason (1973) and Marsh (1979) in excluding *Zele* from the Meteorini and included the genus in Zeleinae (Homolobinae, in the current sense). Shaw’s study, therefore, did not include any species of *Zele* (in the sense of this paper), as his study was focused on determining phylogenetic relationships among the adult-parasitoid euphorines, and used *Meteorus* species only to root the euphorine tree. The Euphorinae, as characterised by Shaw (1985), are characterised by the use of mainly adult insects as host (or late instar nymphs), a unique development absent in *Meteorus* and *Zele*.

The study by Stigenberg et al. (2015) unravelled the phylogenetic relationships of 52 genera within the subfamily Euphorinae by integrating molecular data (18S, 28S, CAD, COI) with morphological and biological (host) data. They proposed an improved classification and placed the Meteorini (including *Meteorus* and *Zele*) as basal tribe within the subfamily Euphorinae, thereby offering a refined perspective on evolutionary relationships within this extremely diverse group. The position of *Zele* within the tribe Meteorini remains uncertain; either differentiated from *Meteorus* (classical view) or nested inside the genus *Meteorus* s.l. (Stigenberg et al. 2015). In the latter case the genus *Meteorus* may have to be subdivided to create monophyletic genera. The most basal placed species of *Zele* in the analysis by Stigenberg et al. (2015) is *Z.caligatus* (Haliday) with dorsope comparatively close to base of the relatively robust tergite and the marginal cell of hind wing approximately 1.5 times wider apically than subbasally. The marginal cell is distinctly narrowed apically compared to the subbasal width in part of *Meteorus* as in Cenocoeliinae (the sister group of Euphorinae). The more posterior position of the dorsope in the slender tergite and the marginal cell of hind wing approximately twice wider apically than subbasally are considered apomorphies in *Zele*. Other evolutionary trends in *Zele* are larger wings, longer ovipositor sheath, legs and antenna, and longer vein 1r-m of hind wing compared to vein 1-M.

Research on this genus in China began in the middle of the last century, with one record of *Z.testaceator* Curtis reported in 1957 (Xia 1957). Thirty years later, Chen et al. (1987) systematically researched *Zele* in China, reporting five species, *Z.caligatus* (Haliday), *Z.chlorophthalmus* (Spinola), *Z.deceptor* (Wesmael), *Z.niveitarsis* (Cresson), and *Z.ruricola* Maetô. Later, three species of *Zele* were also reported from Taiwan province, of which one was a new record for China, *Z.admirabilis* Maetô (Chou and Chou 1993). Finally, a new species of *Zele* was discovered in the Three Gorge Reservoir area of the Yangtze River, viz., *Z.chinensis* Chen & He (Chen et al. 1997). Up to now, the taxonomic study of *Zele* has remained dormant, awaiting further exploration and discovery.

At present, only *Z.chlorophthalmus* has been implemented in biological control, demonstrated to be an effective agent against the cosmopolitan pest *Loxostegesticticalis* L. This parasitoid species exhibits useful biological adaptations, completing its larval development within a single host generation while synchronising its diapause with the overwintering host larvae (Li et al. 2017), highlighting its potential as a biocontrol agent.

## ﻿Materials and methods

The specimens examined in this study were collected by using sweep nets, Malaise traps and light trapping. Most of the specimens examined in this study are deposited in the Parasitic Hymenoptera Collection of Zhejiang University, Hangzhou (**ZJUH**, China). Acronyms for the museums that provided specimens used in this study are **NWU** for the Northwest University, Xian (China); **RMNH** for the Naturalis Biodiversity Center, Leiden (Netherlands); **KBIN** for Koninklijk Belgische Instituut voor Natuurwetenschappen, Brussels (Belgium); **NRMS** for the Naturhistoriska Riksmuseum, Stockholm (Sweden); and **NMI** for the National Museum of Ireland, Dublin (Ireland). After the holotype data the repository of the holotype is indicated by its acronym in brackets.

The terminology and measurements used follow van Achterberg (van Achterberg 1979). The following abbreviations are used: **POL** = postocellar line; **OOL** = ocular-ocellar line, measured from ocellus directly to eye; **OD** = maximum diameter of lateral ocellus; length of the first tergite is measured from the apex of adductor to the apex of tergite in lateral view; width of the first tergite is measured as the maximum width of tergite in dorsal view.

Descriptions and measurements were made under a ZEISS Stemi 305 binocular microscope. Photographs were made with the Keyence (VHX-7000) digital microscope (Keyence Corporation, Osaka, Japan) and the photos were slightly processed (mainly cropped and modification of background) in Adobe Photoshop 2023.

All DNA in this study were was extracted using the QIAamp DNA Mini Kit (Qiagen, Hilden, Germany) and following a non-destructive protocol (Cruaud et al. 2019). For some of the newly described species, DNA was extracted from either the holotype or paratype specimen. Sequence data for these new species, as well as for previously described species included in the analyses, are provided in in Suppl. material 1: table S1. Amplification of approximately 658 bp fragment of COI barcode region (Hebert et al. 2003) was conducted using the primers LCO1490 and HCO2198 (Black et al. 1994). PCR amplifications were performed using KOD One™ PCR Master Mix, and the PCR was run with the following setup: initial denaturation at 98 °C for 5 min and a five-cycle preamplification (30 s at 98 °C, 40 s at 45 °C, and 1 min at 72 °C), followed by 35 cycles of 30 s at 98 °C, 40 s at 55 °C, and 1 min at 72 °C, and a final extension of 5 min at 72 °C.

Sequencing of the final product was performed in both forward and reverse directions and edited using Geneious Prime 2024.0.5. In addition, 102 sequences of congeneric species were downloaded from BOLD Systems v. 3, and one sequence of a single outgroup species were downloaded from NCBI (Suppl. material 1: table S2). All the sequences were translated into amino acids in Geneious Prime 2024.0.5 to identify any stop codons and then aligned using the MAFFT v. 7.505 (Katoh and Standley 2013; Nakamura et al. 2018). The final alignment had a length of 681 bp including undefined nucleotides (N) for some sequences.

Sequence divergences for intraspecific and interspecific pairwise genetic distances were computed based on the Kimura-2parameter (K2P) model in MEGA-X (Kumar et al. 2018) (Suppl. material 2). Maximum-likelihood (ML) analyses were performed using IQ-TREE v. 2.1.3 (Nguyen et al. 2015) and the best-fitting substitution model was identified using Model Finder implemented in IQ-TREE (MFP). FigTree v. 1.4.4 was utilised to visualise and illustrate the inferred phylogenetic trees.

Two different methods were used for species delimitation: the distance-based method Automatic Barcode Gap Discovery (ABGD) and the tree-based method Poisson Tree Process (PTP) (Zhang et al. 2013). The ABGD analysis was used for species delimitation, automatically partitioning sequences into candidate species based on the barcode gap (difference between intra- and inter-specific variation) without needing a priori thresholds (Puillandre et al. 2012). The ABGD analysis was conducted via a web interface (https://bioinfo.mnhn.fr/abi/public/abgd/abgdweb.html), using the K2P model to classify species based on genetic distances. The relative gap width (X) was set to 1.0, and the remaining parameters were set to default. The bPTP method was performed online (https://species.h-its.org/ptp/), with unrooted selected for tree type, 500,000 specified for NO. MCMC generations, and default parameters used for the rest.

## ﻿Results

### ﻿Species delimitation

The COI fragments were sequenced from 13 new species and four known species deposited in GenBank (accession numbers in Suppl. material 1: table S1). Despite the comprehensive use of multiple methods, we could not obtain the COI sequences for dry pinned specimens. Pairwise distances were estimated by using the P-distance model with the option for pairwise deletion. Interspecific distance ranged from 0.0276 to 0.3493 (Suppl. material 2).

**Figure 1.**
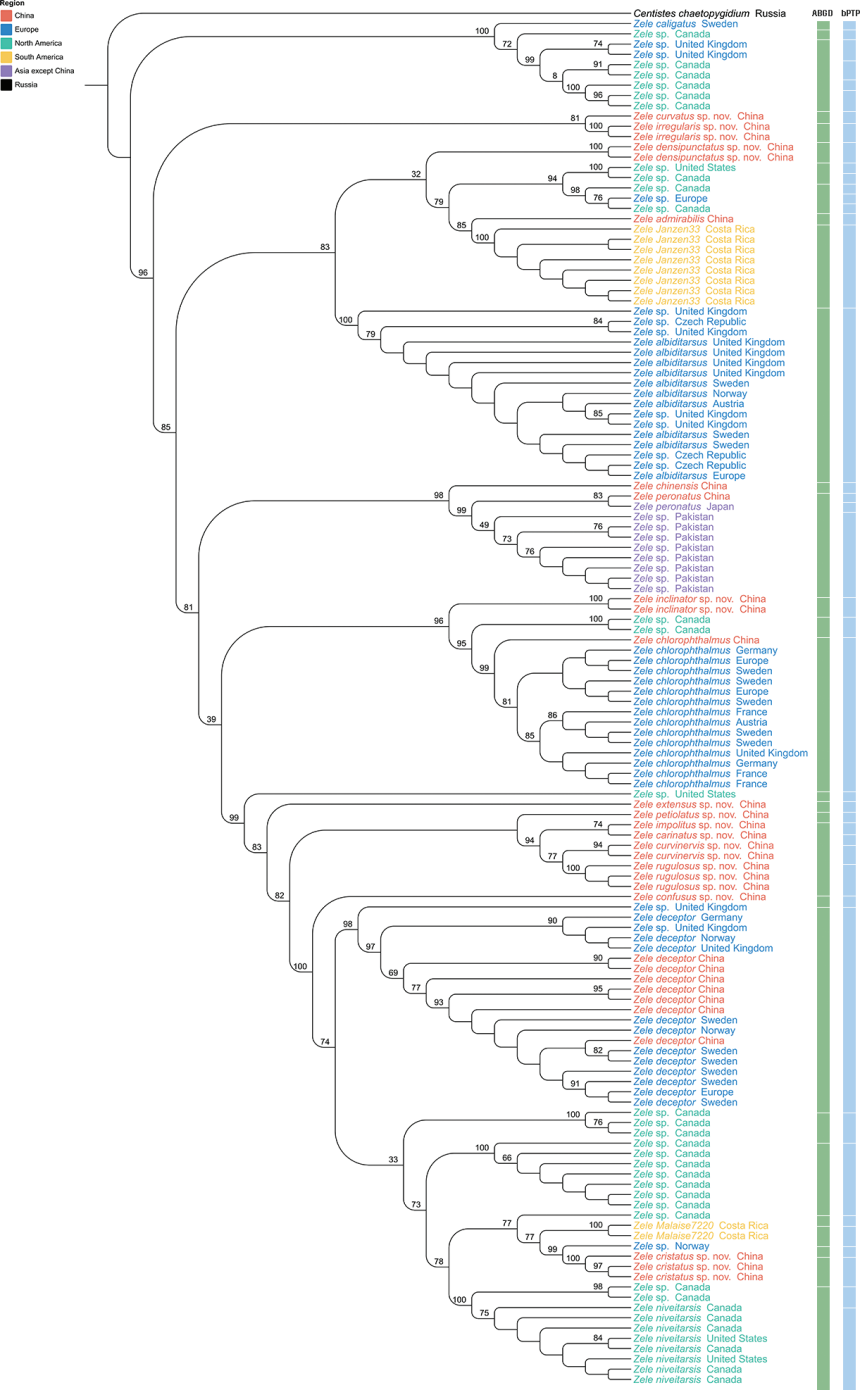
ML phylogenetic tree based on 134 COI sequences highlighting the results of two delimitation analyses in *Zele*. Bootstrap values are shown below the branches. The results of delimitation analyses are displayed with the vertical bars corresponding to putative species (MOTUs) inferred by ABGD and bPTP methods.

Species delimitation results from the two approaches are summarised in Fig. 1. The ABGD analysis divided COI sequences of the *Zele* group into 29 molecular operational taxonomic units (MOTUs), but the bPTP analysis divided the group into 42 MOTUs. We partly adopted the bPTP approach for delineating the MOTUs, when we found a combination of both analyses showing the best correspondence with the morphological differences observed among the specimens.

In summary, 19 new species were identified: seven species (*Z.aquilus* sp. nov., *Z.fulgidus* sp. nov., *Z.fuscatus* sp. nov., *Z.sculpticoxis* sp. nov., *Z.shaanxiensis* sp. nov., *Z.syntomus* sp. nov., and *Z.vacatus* sp. nov.) are only based on morphological evidence, and 12 species are supported by both morphological and molecular evidence. In addition, an identification key to species of *Zele* from China is provided in this paper.

### ﻿Taxonomy

#### ﻿ Zele

Taxon classificationAnimaliaHymenopteraBraconidae

Curtis, 1832

1267E553-E6B4-51E5-B3A4-C5513BB62274

Zele Curtis, 1832: 415. Type species (by original designation): Zeletestaceator Curtis, 1832 (= Zelealbiditarsus Curtis, 1832; van Achterberg, 1979: 359). Type locality: U.K. • ♀; England, Coomb Wood; 25 Jul.; (Melbourne Museum, Carlton).

##### Diagnosis.

Antescutal depression of pronotum absent; first tergite almost always not or only slightly narrowed behind spiracles and spiracle situated submedially or behind middle of tergite; first metasomal tergite petiolate or very elongate and dorsal carinae absent; The genus can be recognised from other genera of Euphorinae by the presence of the vein r-m of the fore wing, the second submarginal cell of fore wing rhomboid or quadrate; an apically widened marginal cell of the hind wing (but sometimes only slightly so), and largely setose fourth and fifth metasomal tergites (but specimens stored for long time in alcohol may have lost their setae).

##### Distribution.

Widespread, but absent in the Afrotropical and Australian regions.

##### Biology.

Koinobiont endoparasitoids of larvae of Lepidoptera species belonging to the families Arctiidae, Gelechiidae, Geometridae, Hepialidae, Lasiocampidae, Limacodiadae, Lymantriidae, Noctuidae, Pyralidae, and Saturniidae.

### ﻿Key to Chinese species of the genus *Zele* Curtis

**Table:** 

1	Setose part of ovipositor sheath 0.41–0.61× fore wing and ovipositor slender subbasally (Fig. 7F, D); **if** intermediate then pterostigma largely dark brown or blackish; vein cu-a of fore wing often antefurcal or interstitial	**2**
–	Setose part of ovipositor sheath 0.19–0.38× fore wing and comparatively robust (Fig. 4F, D); **if** intermediate then pterostigma largely brownish yellow or yellow; vein cu-a of fore wing postfurcal or interstitial, rarely antefurcal	**8**
2	Vein 1r-m of hind wing 3.3–5.2× longer than vein 1-M; frons and vertex distinctly punctate (Figs 6G, 25H)	**3**
–	Vein 1r-m of hind wing 1.6–2.7× longer than vein 1-M; frons and vertex often punctulate or nearly smooth (Figs 16G, 21G)	**5**
3	Head in dorsal view black or largely so and contrasting with reddish brown mesosoma (Figs 6C, G); scutellar sulcus shallow antero-laterally; apical 1/2 of hind tibia dark brown; middle of hind femur and apex of hind tibia dark brown (Fig. 6A, M); area in front of lateral ocellus only finely punctate; ocelli larger, OD 1.2–1.5× POL (Fig. 6G)	***Z.chinensis* Chen & He, 1997**
–	Head and mesosoma in dorsal view not contrasting and both black (Fig. 7H, C); scutellar sulcus deep antero-laterally, rarely intermediate (Figs 20C, 25C); apical 1/3 or 2/3 of hind tibia dark brown (Figs 20A, 25A); middle of hind femur brown or dark brown, paler than blackish brown apex of hind tibia; area in front of lateral ocellus more or less depressed and more or less sculptured; ocelli smaller, OD 0.8–1.0× POL (Figs 20H, 25H)	**4**
4	Hind coxa densely punctate-rugulose, rather matt, and without distinct smooth interspaces (Fig. 26A); dorsope of first tergite small and narrow elliptical (Fig. 26K); hind tibia (except apical 1/3) ivory; frons usually partly rugose (Fig. 26G); base of hind basitarsus only infuscated	***Z.sculpticoxis* sp. nov.**
–	Hind coxa punctate and with small smooth interspaces, rather shiny (Fig. 20A); dorsope of first tergite large and oval (Fig. 20K); hind tibia (except basal 1/4) dark brown or brown; frons largely smooth, rugulose or finely carinate (Fig. 20H); base of hind basitarsus often blackish brown, rarely infuscated	***Z.peronatus* (Shestakov, 1940), reinstated**
5	Length of first metasomal tergite ~1.7× its apical width, first tergite robust, petiolate part shorter, 0.2× length of first tergite, dorsope comparatively close to base of tergite, dorsope in basal of 0.2× first tergite (Fig. 15J, K); precoxal sulcus comparatively narrowly sculptured anteriorly and mesopleuron shiny (Fig. 15B); eyes in dorsal view of ♀ 1.2× as long as temple (Fig. 15H); malar space 0.6× as long as basal width of mandible; scutellum yellowish, strongly contrasting with dark brown mesosoma (Fig. 15C); pterostigma of ♀ pale yellowish (Fig. 15D)	***Z.fulgidus* sp. nov.**
–	Length of first metasomal tergite 2.1–2.8× its apical width, first tergite slender, petiolate part longer, 0.45–0.50× length of first tergite, dorsope far from base of tergite, dorsope in basal of 0.35–0.40× first tergite (Fig. 16I, J); precoxal sulcus comparatively widely sculptured anteriorly and mesopleuron less shiny (Figs 16B, 21B); eyes in dorsal view of ♀ 1.8–2.3× as long as temple (Figs 7H, 21B); malar space 0.1–0.4× as long as basal width of mandible; scutellum yellowish or blackish, weakly contrasting with mesosoma (Figs 7C, 16C); colour of pterostigma of ♀ variable	**6**
6	Malar space 0.4× as long as basal width of mandible (Fig. 16H); pterostigma of ♀ dark brown (Fig. 16D); ventral 1/2 of temple yellowish, strongly contrasting with blackish mesosoma (Fig. 16G, C); face 1.4× wider than high (Fig. 16F)	***Z.fuscatus* sp. nov.**
–	Malar space 0.1–0.3× as long as basal width of mandible (Figs 7I, 21H); pterostigma of ♀ yellowish or pale brown (Figs 7D, 21D); ventral 1/2 of temple brown or yellowish and weakly contrasting with mesosoma (Figs 7A, 21A); face 1.0–1.2× wider than high (Figs 7G, 21F)	**7**
7	Pterostigma of both sexes yellowish, laterally more or less darkened (Fig. 7D); length of first metasomal tergite 2.1–2.4× its apical width (Fig. 7J, K); first tergite less narrowed in front of dorsope (Fig. 7K); hind tarsus yellowish medially, similar to apex of hind tibia or nearly so (Fig. 7A); setae of ovipositor sheath less erect, more slanted (Fig. 7F)	***Z.chlorophthalmus* (Spinola, 1808)**
–	Pterostigma of ♀ (♂ unknown) pale brown (Fig. 21D); length of first metasomal tergite 2.7–2.9× its apical width (Fig. 21I, J); first tergite obviously narrowed in front of dorsope (Fig. 21J); hind tarsus white or ivory medially, distinctly contrasting with apex of hind tibia (Fig. 21A); setae of ovipositor sheath conspicuous and erect or semi-erect (rather conspicuous: Fig. 21E)	***Z.petiolatus* sp. nov.**
8	Densely sculptured area of precoxal sulcus medium-sized or narrow medially (Fig. 18B); hind tibia and tarsus yellowish or infuscated; ocelli comparatively small, POL 1.7–1.8× diameter of posterior ocellus (Fig. 18H); first metasomal tergite comparatively robust, 1.7–2.2× longer than its apical width (Fig. 18K); [ovipositor sheath 0.19–0.28× as long as fore wing]	**9**
–	Densely sculptured area of precoxal sulcus wide medially (Figs 3B, 9B, 24B), **if** medium-sized (*Z.aquilus*, males of *Z.ruricola*) **then** hind tarsus ivory or whitish; ocelli large, POL approximately equal to diameter of posterior ocellus, rarely somewhat longer (Fig. 10G); first metasomal tergite usually comparatively slender, 2.3–4.0× longer than its apical width (Figs 10J, 19L), but first tergite comparatively robust (1.7–2.0× longer than its apical width) in *Z.romani* and *Z.ruricola*	**10**
9	Marginal cell of hind wing strongly widened apically (Fig. 18E); densely sculptured area of precoxal sulcus medium-sized medially (Fig. 18B); malar space ~0.2× as long as basal width of mandible; metanotum with enlarged smooth knob medio-posteriorly and median carina in front of it short (Fig. 18C); [vein cu-a of fore wing slightly oblique (Fig. 18D)]	***Z.inclinator* sp. nov.**
–	Marginal cell of hind wing slightly widened apically (fig. 758 in van Achterberg 1979); sculptured area of precoxal sulcus narrow medially; malar space ~0.4× as long as basal width of mandible; metanotum with small smooth knob medio-posteriorly and median carina in front of it absent; [basal 1/2 of propodeum largely smooth and shiny (except slightly curved subbasal carina)]	***Z.caligatus* (Haliday, 1835)**
10	Length of first metasomal tergite 3.5–4.0× its apical width (Fig. 2K, L); second tergite densely setose, very finely sculptured and rather matt; first tergite with fine longitudinal carina behind level of dorsope (Fig. 2L); hind coxa distinctly punctate and with some rugae dorsally; [pterostigma of ♀ and ♂ yellowish brown (Fig. 2D); precoxal sulcus very densely rugose (Fig. 2B); fore wing and first subdiscal cell wide; vein r of hind wing vaguely indicated (Fig. 2E); eye of ♀ 2.9–3.2× as long as temple in dorsal view]	***Z.admirabilis* Maetô, 1986**
–	Length of first metasomal tergite 1.6–2.8× its apical width; second tergite largely bare, smooth and shiny; first tergite without fine medio-longitudinal carina; hind coxa less sculptured and without rugae (Figs 12C, 22C)	**11**
11	Eyes comparatively large (Fig. 10G), in dorsal view of ♀ ~3.6× as long as temple; dorsope comparatively large, and space between dorsope approximately equal to width of dorsope (Fig. 10J); temple directly narrowed and lowered behind eye (Fig. 10G, H); [vein r of hind wing vaguely present (Fig. 10D); outline of propodeum rather curved in lateral view (Fig. 10B); subbasal carina of propodeum rather arched and median carina in front of carina comparatively long]	***Z.curvatus* sp. nov.**
–	Eyes smaller (Figs 9G; 19I), in dorsal view of ♀ 1.4–2.7× as long as temple; dorsope comparatively small, and area between dorsope slightly or distinctly wider than dorsope (Fig. 10J), **if** dorsope comparatively large **then** entire middle lobe of mesoscutum densely punctate and rather dull (*Z.densipunctatus*); temple usually gradually narrowed and lowered behind eye (Figs 9G, H, 19I, J)	**12**
12	Vein 2-SC+R of hind wing distinctly longer than vein 1-M (Fig. 14E); metanotum with three distinct parallel carinae and no knob medio-posteriorly (Fig. 14C); tip of ovipositor sheath dark brown (Fig. 14F) [basal 1/3 of first tergite comparatively wide in lateral view (Fig. 14K); eyes distinctly protruding in dorsal view; fore wing and first subdiscal cell comparatively narrow; pterostigma infuscated (Fig. 14D)]	***Z.extensus* sp. nov.**
–	Vein 2-SC+R of hind wing approximately as long or shorter than vein 1-M (Figs 8D, 28D), but only slightly longer in *Z.romani* (Fig. 22E); metanotum with two distinct and more or less converging carinae and with knob medio-posteriorly (Fig. 23C); **if** with one median carina (*Z.cristatus*) **then** carina short and knob present (Fig. 9C); tip of ovipositor sheath usually pale yellowish (Figs 11F, 19E)	**13**
13	Ovipositor comparatively slender subbasally and setose part of its sheath 0.34–0.40× as long as fore wing (Fig. 25F, D); length of hind femur of ♀ 5.5–6.2× its maximum width (Fig. 25L); length of first metasomal tergite 1.6–1.9× its apical width, robust (Fig. 25J, K); [notauli narrow and only very finely crenulate anteriorly (Fig. 25C)]	***Z.ruricola* Maetô, 1986**
–	Ovipositor more robust subbasally and setose part of its sheath 0.19–0.33× as long as fore wing; length of hind femur 6.2–9.0× its maximum width; length of first tergite 1.8–2.8× its apical width, **if** length of first tergite 1.8–2.0× its apical width **then** fore tibial spur 0.4–0.5× as long as fore basitarsus and vein r of hind wing present (*Z.albiditarsus*)	**14**
14	Subbasal carina of propodeum close to anterior margin of propodeum (Fig. 3C); fore femur robust, 5.0–5.5× as long as wide, rarely up to 6.2× (Fig. 3L); fore tibial spur comparatively well developed, 0.4–0.5× as long as fore basitarsus (Fig. 3M); vein r of hind wing present as vague (“spectral”) and more or less pigmented vein (Fig. 3E); [propodeum with long median carina (Fig. 3C)]	***Z.albiditarsus* Curtis, 1832**
–	Subbasal carina more or less removed from anterior margin of propodeum (Figs 13C, 23C); fore femur comparatively slender, 6.0–10.0× as long as wide; fore tibial spur comparatively less developed, 0.3–0.4× as long as fore basitarsus (Fig. 10M); vein r of hind wing usually absent (but present in *Z.irregularis*)	**15**
15	Vein 1r-m of hind wing ~7.0× longer than vein 1-M (Fig. 22E); first metasomal tergite slightly robust (~2.0× longer than its apical width), dorsope comparatively close to base of tergite (dorsope in basal of 0.27× first tergite) and tergite largely smooth (Fig. 22J, K); head largely dark brown (except yellowish clypeus and mandible) (Fig. 22G, H); precoxal sulcus area mainly coarsely punctate; [hind tarsus largely ivory or white; length of malar space 0.2× basal width of mandible; pedicellus yellowish; hind coxa, first and second tergites dark brown; length of hind femur ~6.3× as long as wide (Fig. 22M); length of eye 2.3–2.4× temple] [not yet found in China, but most likely occurring in northeast China]	***Z.romani* (Fahringer, 1929), reinstated**
–	Vein 1r-m of hind wing 1.6–4.0× as long as vein 1-M (Fig. 12D); first metasomal tergite usually slender (2.3–2.8× longer than its apical width), dorsope comparatively far from base of tergite (dorsope in basal of 0.32–0.37× first tergite) and tergite largely sculptured (Fig. 17K), **if** smooth **then** segments of apical 1/3 of antenna of ♀ shortened and ~1.4× longer than wide (*Z.syntomus*); head often yellowish brown (Fig. 12G) or pale brown; precoxal sulcus rugose or densely rugulose	**16**
16	Propodeum with coarse and irregular sculpture including subbasal carina (Fig. 19C, B) propodeum oblique anteriorly in front of subbasal carina (Fig. 19B); fore femur comparatively robust, 6.0–6.3× as long as wide; laterally mesosoma usually more darkened and contrasting with dorsal part; vein r of hind wing present as unsclerotised vein (Fig. 19D); [vein 1r-m of hind wing comparatively long, vein 1r-m of hind wing 1.6–2.0× as long as vein 1-M; fore tibial spur medium-sized (Fig. 19G); dorsope of first tergite medium-sized and area between dorsope wider than dorsope, basal part of first tergite in front of dorsope largely smooth (Fig. 19L)]	***Z.irregularis* sp. nov.**
–	Propodeal sculpture less coarse and usually more regular (Figs 11C, 23C), **if** coarsely sculptured (Fig. 9C) **then** basal part of first tergite distinctly sculptured in front of small dorsope (Fig. 9J); propodeum horizontal anteriorly (Fig. 9B); fore femur comparatively slender, 7.0–9.5× as long as wide; laterally colour of mesosoma usually less contrasting with dorsal part; vein r of hind wing usually absent	**17**
17	Malar space comparatively long in anterior view (Fig. 8F) and its length 0.4–0.5× basal width of mandible; face comparatively wide, face 1.4–1.6× wider than high (Fig. 29F)	**18**
–	Malar space short in anterior view (Figs 7F, 23G) and its length 0.2–0.3× basal width of mandible; face comparatively narrow, face 1.0–1.4× wider than high (Figs 10F, 13F)	**20**
18	Segments of apical 1/3 of antenna of ♀ ~1.4× longer than wide (Fig. 28O); first tergite shiny and with comparatively large dorsope and part behind dorsope smooth or finely sculptured (Fig. 28K); hind coxa largely smooth or finely punctulate dorsally; pterostigma of ♀ pale yellowish (Fig. 28D); eyes length of eye 2.0–2.2× temple in dorsal view	***Z.syntomus* sp. nov.**
–	Segments of apical 1/3 of antenna of ♀ at least ~2.0× longer than wide (Fig. 8A); first tergite less shiny and with comparatively small dorsope and part behind dorsope irregular and obvious rugose (Fig. 8J); hind coxa densely punctate dorsally; pterostigma of ♀ brown or pale brown (Fig. 8D); length of eye 1.4–1.8× temple in dorsal view	**19**
19	Hind femur slender, basal part of ovipositor comparatively robust, maximum width of basal part of ovipositor 0.6× maximum width of hind femur (Fig. 8L, M); subbasal transverse carina of propodeum well developed, different from surrounding sculpture; hind femur largely black, only apically brown and hind tibia mostly (except basal 1/3) black (Fig. 8A); [fore femur slender, ~9.5× as long as wide (Fig. 8K)]	***Z.confusus* sp. nov.**
–	Hind femur robust, basal part of ovipositor comparatively slender, maximum width of basal part of ovipositor 0.4× maximum width of hind femur (Fig. 29L, M); subbasal transverse carina of propodeum not discernible from surrounding sculpture (Fig. 29C); hind femur reddish brown and apical 1/2 of hind tibia dark brown (Fig. 29A); [fore femur robust, ~7.4× as long as wide (Fig. 29K)]	***Z.vacatus* sp. nov.**
20	Metanotum with enlarged smooth knob medio-posteriorly and median carina in front of it comparatively short (Fig. 4C); precoxal sulcus narrowly sculptured and mesopleuron shiny (Fig. 4B); vein 1r-m of hind wing 1.8–2.2× longer than vein 1-M (Fig. 4E); pterostigma of ♀ dark brown (Fig. 4D); [hind femur 6.0–7.0× as long as wide; first tergite costate medio-posteriorly (Fig. 4K]	***Z.aquilus* sp. nov.**
–	Metanotum with small smooth knob medio-posteriorly and median carina in front of it comparatively long (Fig. 11C); precoxal sulcus widely sculptured and mesopleuron less shiny; vein 1r-m of hind wing 2.5–4.0× longer than vein 1-M (Fig. 4E); pterostigma of ♀ usually yellowish brown, infuscated or pale yellowish (Fig. 5C)	**21**
21	Entire middle lobe of mesoscutum densely punctate and rather dull, mesoscutum and without rugae or rugulae medio-posteriorly (Fig. 13C); dorsope of first tergite large and space between dorsope approximately as wide as dorsope (Fig. 13J); length of first tergite 2.6–2.8× its maximum width (Fig. 13I, J); face 1.3–1.4× wider than high (Fig. 13F); [hind femur 7.5–9.0× longer than wide (Fig. 13L)]	***Z.densipunctatus* sp. nov.**
–	Anterior 1/2 of middle lobe of mesoscutum smooth or densely punctulate and often shiny, and mesoscutum medio-posteriorly more or less rugulose or rugose; dorsope of first tergite small and space between dorsope wider than dorsope (Fig. 13J); length of first tergite 2.1–2.6× its maximum width; width of face 1.0–1.3× its height	**22**
22	Third metasomal tergite strongly shiny and black; hind femur black or nearly so; first metasomal tergite black and its dorsope minute, rarely elongate; fore wing and its first subdiscal cell often comparatively wide (Figs 11D, 23D); [propodeum latero-posteriorly with protruding corners in dorsal view]	**23**
–	Third tergite less shiny and yellowish or hind femur entirely dark reddish brown or brownish yellow, but may be dark brown in *Z.cristatus*; first tergite often yellowish brown and its dorsope often medium-sized, but black or dark reddish brown and with small dorsope in *Z.cristatus*; fore wing and its first subdiscal cell narrower (Fig. 9D)	**27**
23	Pterostigma dark brown or brown (Fig. 5D) and mandible yellow (except dark apex) (Fig. 5F); subbasal carina of propodeum distinctly oblique medially (Fig. 5C); propodeum depressed posteriorly and slightly shorter in lateral view (Fig. 5B); mesoscutum medio-posteriorly with short longitudinal carina (Fig. 5C); [vein r of fore wing oblique]	***Z.carinatus* sp. nov.**
–	Pterostigma pale yellow (Fig. 11D) and/or mandible dark brown (Fig. 23B); subbasal carina of propodeum transverse or weakly curved medially (Figs 11C, 17C, 23C); propodeum not depressed posteriorly (except in *Z.rugulosus*; Fig. 23B) and slightly longer in lateral view (Figs 11B, 17B); mesoscutum medio-posteriorly with longitudinal carina (Figs 11C, 17C)	**24**
24	Pterostigma partly infuscated (Fig. 17D); lateral lobes of mesoscutum comparatively matt and remotely punctulate; metanotum with three medium-sized carinae dorsally and posterior knob keeled (Fig. 17C); anteriorly propodeum superficially sculptured between carinae (Fig. 17C); [mandible dark brown or brown; laterope wide elliptical (Fig. 17K)]	***Z.impolitus* sp. nov.**
–	Pterostigma pale yellowish, at most its posterior rim slightly infuscated (Figs 11D, 23D); lateral lobes of mesoscutum comparatively shiny and densely punctulate; metanotum with two rather long carinae dorsally and posterior knob without keel; anteriorly propodeum remotely sculptured between carinae (Figs 11C, 23C)	**25**
25	Propodeum depressed posteriorly in lateral view (Fig. 24B); mandible blackish (Fig. 24I); first tergite more widened posteriorly (compared to its minimum width, Fig. 24K)	***Z.rugulosus* sp. nov.**
–	Propodeum gradually lowered posteriorly in lateral view (Fig. 11B); mandible yellowish brown (except dark apex: Fig. 11I); first tergite less widened posteriorly (Fig. 11K)	**26**
26	Eyes more protruding and temples more directly narrowed in dorsal view (Fig. 11H); vein m-cu of fore wing slightly curved (Fig. 11D); anterior tentorial pits close to eyes (Fig. 11G); second tergite black	***Z.curvinervis* sp. nov.**
–	Eyes less protruding and temples less directly narrowed in dorsal view (Fig. 27H); vein m-cu of fore wing straight (Fig. 27D); anterior tentorial pits distinctly removed from eyes (Fig. 27G); second tergite dark brown basally	***Z.shaanxiensis* sp. nov.**
27	Posterior oblique part of propodeum usually distinctly differentiated from anterior part; propodeum with irregular subbasal carina and dorsal face of propodeum with more or less developed posterior crest, and dorsally behind transverse carina with distinct rugae (Fig. 9C); first metasomal tergite dark brown or blackish, at most its apical 1/3 brown and part in front of dorsope sculptured (Fig. 9J); dorsope hardly visible in lateral view and laterope medium-sized (Fig. 9I)	***Z.cristatus* sp. nov.**
–	Posterior oblique part of propodeum not or hardly differentiated from anterior part; subbasal carina more regular and dorsal face without posterior crest and often with weak intermittent sculpture (Figs 12C, 22C); first metasomal tergite yellowish brown and smooth in front of dorsope (Figs 12J, 22K); dorsope distinct in lateral view and laterope deep, large (Figs 12J, 22K)	**28**
28	Subbasal carina of propodeum medially subparallel with anterior margin of propodeum (carina rather irregular and area somewhat wider laterally) and enclosed area comparatively wide and rugose (Fig. 23C); vein 1-M of hind wing distinctly wider than vein M+CU (Fig. 23E); hind tarsus largely whitish	***Z.rufulus* (Thomson, 1895), reinstated**
–	Subbasal carina of propodeum distinct diverging from anterior margin of propodeum medially with enclosed area narrow medially and superficially sculptured (Fig. 12C); vein 1-M of hind wing approximately as wide as vein M+CU (Fig. 12D); hind tarsus brownish yellow or yellowish brown, rarely pale yellowish	***Z.deceptor* (Wesmael, 1835)**

#### ﻿ Zele admirabilis

Taxon classificationAnimaliaHymenopteraBraconidae

Maetô, 1986

C0D4DC7C-45D5-503C-BB28-065A5E3B18D2

Fig. 2

Zele admirabilis Maetô, 1986: 250; Chou and Chou 1993: 447; Belokobylskij 2000: 223.

##### Material examined.

China – **Guangxi Prov.** • 1 ♂; Guilin, Mt. Maoer; 8 Aug. 2005; Wen-wu Shang leg.; (ZJUH) No. 202401072. GenBank accession no. PV356321.

##### Diagnosis.

**Figure 2.**
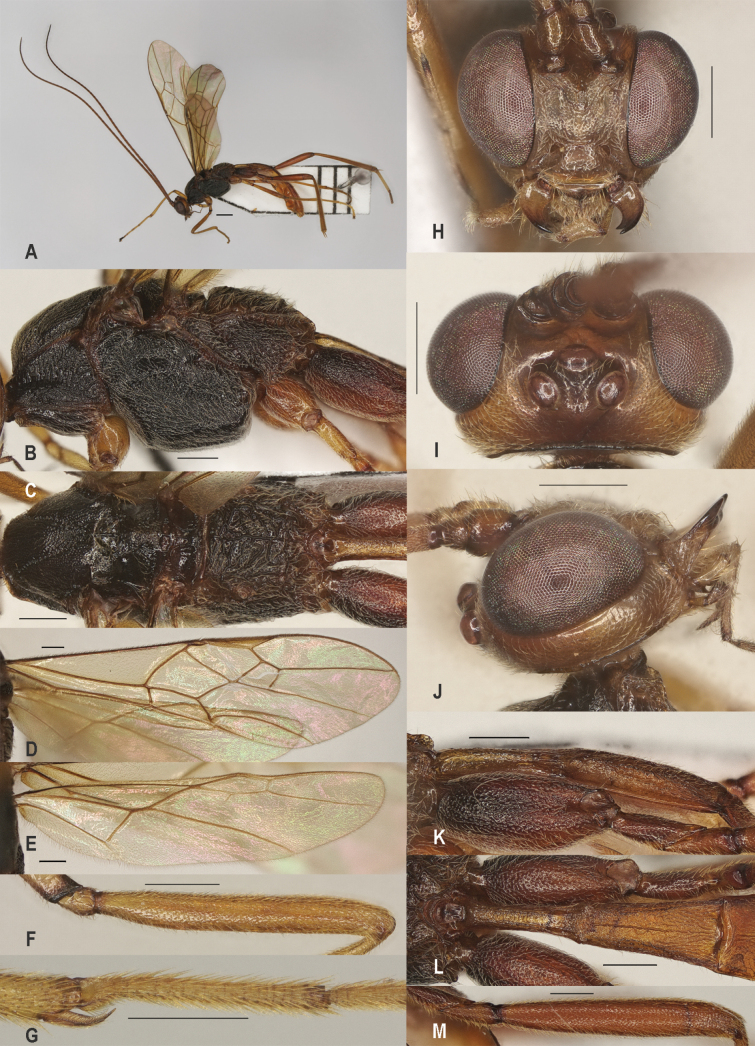
*Zeleadmirabilis* Maetô, China, Guangxi, ♂ A. Habitus, lateral aspect; B. Mesosoma, lateral aspect; C. Mesosoma, dorsal aspect; D. Fore wing; E. Hind wing; F. Fore femur, lateral aspect; G. Fore tibial spur and fore basitarsus; H. Head, anterior aspect; I. Head, dorsal aspect; J. Head, lateral aspect; K. First metasomal tergite, lateral aspect; L. First metasomal tergite, dorsal aspect; M. Hind femur, lateral aspect. Scale bars: 1000 μm (A); 500 μm (B–M).

Length of first metasomal tergite 3.5–4.0× its apical width (Fig. 2K, L); first tergite yellowish brown, with a longitudinal carina behind dorsope (Fig. 2L); dorsope narrow and space between dorsope much wide than dorsope (Fig. 2L); second tergite densely setose, very finely sculptured and rather matt; hind coxa distinctly punctate and with some rugae dorsally; basal 1/2 of fore wing pale brownish; pterostigma of ♀ and ♂ yellowish brown (Fig. 2D); precoxal sulcus very densely rugose (Fig. 2B), but more or less reduced in males; fore wing and first subdiscal cell wide; vein r of hind wing vaguely indicated (Fig. 2E); hind tarsus mainly white; ovipositor sheath ~0.35× as long as fore wing.

##### Distribution.

China (Guangxi, Taiwan), Japan, Russia (south of Far East).

##### Biology.

Unknown.

#### ﻿ Zele albiditarsus

Taxon classificationAnimaliaHymenopteraBraconidae

Curtis, 1832

4A10F9DB-A103-5F37-8F4E-4C5821528F23

Fig. 3

Zele albiditarsus Curtis, 1832: 415.
Zele testaceator Curtis, 1832: 415; Xia 1957: 311. Syn. by van Achterberg 1979.
Meteorus albitarsis Haliday, 1835: 24. Syn. by van Achterberg 1979.
Zele albiditarsus f. albiditarsus : van Achterberg 1979: 380–383.
Zele albiditarsus : van Achterberg 1984: 110; Chen et al. 1987: 94.

##### Type material examined.

***Lectotype*** of *Zeletestaceator*. U.K. • ♀; England, Coomb Wood; 25 Jul.; (Melbourne Museum, Carlton).

##### Other material examined.

China – **Henan Prov.** • 1 ♀; Jiyuan; 5 Jun. 2000; Ping Cai leg.; (ZJUH) No. 200102002. – **Hunan Prov.** • 1 ♀; Liuyang; 7 Jul. 1977; Yan-cheng Zhang leg.; (ZJUH) No. 790016. • 2 ♀♀; Liuyang; 21 Oct. 1985; Xin-wang Tong leg.; (ZJUH) Nos. 864622, 8646223. – **Jilin Prov.** • 1 ♀; Mt. Changbai; 7 Jul. 1977; Yan-cheng Zhang leg.; (ZJUH) No. 790016. • 1 ♀; Mt. Changbai; 4 Jul. 1981; Jin-kuan Yang leg.; (ZJUH) No. 948240. – **Heilongjiang Prov.** • 3 ♀♀; Harbin; Jun. 1986; Lv-hong Zhang leg.; (ZJUH) Nos. 948201–948203. – **Shaanxi Prov.** • 1 ♀; Xunyangba, Ningshan; 33.55°N, 108.55°E; alt. 1481 m; 20 May–23 Jun. 2016; Jiang-li Tan, Qing-Qing Tan leg; Green Malaise trap; (ZJUH) No. 202315009. • 1 ♂; same locality as for preceding; 1 Jul.–17 Aug. 2016; Jiang-li Tan, Qing-Qing Tan leg; Yellow and Green Malaise trap; (ZJUH) No. 202315010.

##### Diagnosis.

**Figure 3.**
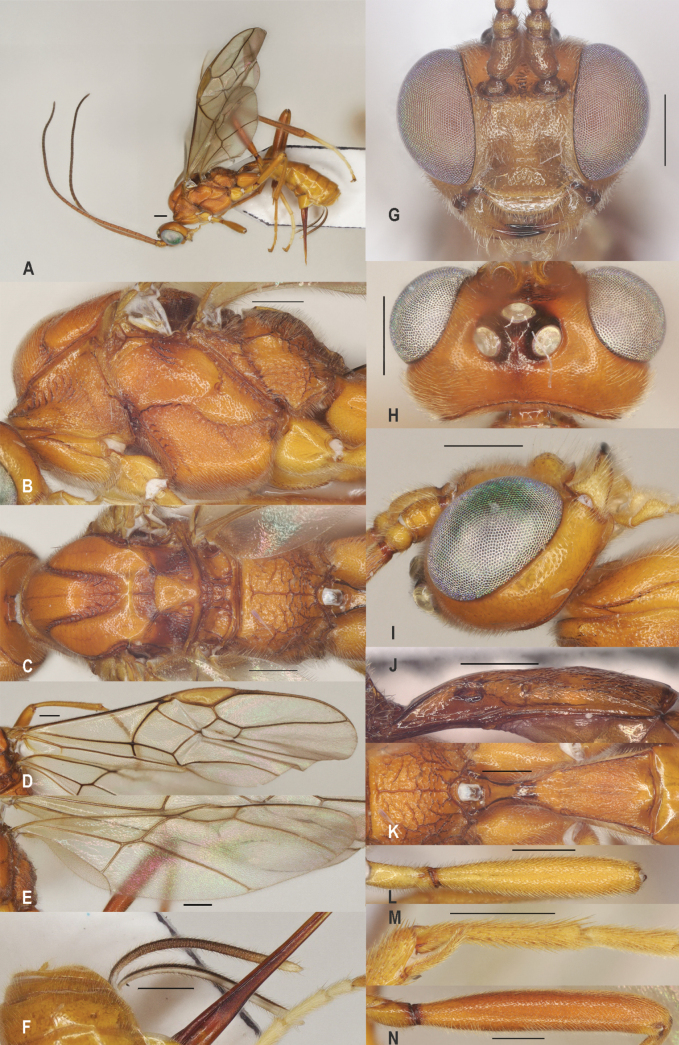
*Zelealbiditarsus* Curtis, China, Shaanxi, ♀ A. Habitus, lateral aspect; B. Mesosoma, lateral aspect; C. Mesosoma, dorsal aspect; D. Fore wing; E. Hind wing; F. Ovipositor sheath; G. Head, anterior aspect; H. Head, dorsal aspect; I. Head, lateral aspect; J. First metasomal tergite, lateral aspect; K. First metasomal tergite, dorsal aspect; L. Fore femur, lateral aspect; M. Fore tibial spur and fore basitarsus; N. Hind femur, lateral aspect. Scale bars: 500 μm.

Subbasal carina of propodeum close to anterior margin of propodeum (Fig. 3C); fore femur robust, 5.0–5.5× as long as wide, rarely up to 6.2× (Fig. 3L); fore tibial spur comparatively well developed, 0.4–0.5× as long as fore basitarsus (Fig. 3M); vein r of hind wing present as vague (“spectral”) and more or less pigmented vein (Fig. 3E); first tergite 1.8–2.5×, rarely up to 2.8× longer than its apical width; dorsope rather large, space between dorsope slightly wider than dorsope (Fig. 3K); hind tarsus mainly white; ovipositor sheath 0.21–0.30× as long as fore wing; propodeum with long median carina, comparatively reticulate (Fig. 3C).

##### Distribution.

Armenia, Austria, Azerbaijan, Belgium, Bulgaria, China (Fujian, Guizhou, Heilongjiang, Hubei, Hunan, Jilin, Sichuan), Croatia, Cyprus, Czech Republic, Denmark, Finland, France, Georgia, Germany, Hungary, India, Iran, Ireland, Italy, Japan, Kazakhstan, Korea, Latvia, Lithuania, Moldova, Nepal, Netherlands, Norway, Poland, Romania, Russia, Slovakia, Sweden, Switzerland, Turkey, United Kingdom, former Yugoslavia.

##### Biology.

Mainly reared from Noctuidae, but some also Geometridae. Specimens reared from other host groups need confirmation.

#### ﻿ Zele aquilus

Taxon classificationAnimaliaHymenopteraBraconidae

Fang, van Achterberg & Chen sp. nov.

81743AC0-22F0-5C0E-A26B-AD94132F66A2

https://zoobank.org/9B6ABD9C-2EB5-4105-81F9-A83231EFE0D0

Fig. 4

##### Type material.

***Holotype*.** China – **Shaanxi Prov.** • ♀; Pingheliang, Ningshan; 33.47°N, 108.50°E; alt. 2105 m; 25 Jul.–22 Oct. 2017; Jiang-li Tan, Qing-qing Tan leg.; B[lack] Malaise trap; (ZJUH) No. 202315012. ***Paratypes*.** China – **Shaanxi Prov.** • 1 ♀; same data as for holotype; (ZJUH) No. 202315002. • 1 ♀; Pingheliang, Ningshan; alt. 2188 m; 17 Aug.–1 Oct. 2016; Jiang-li Tan, Qing-qing Tan leg.; B[lack] Malaise trap; (NWU) No. 202315001.

##### Diagnosis.

**Figure 4.**
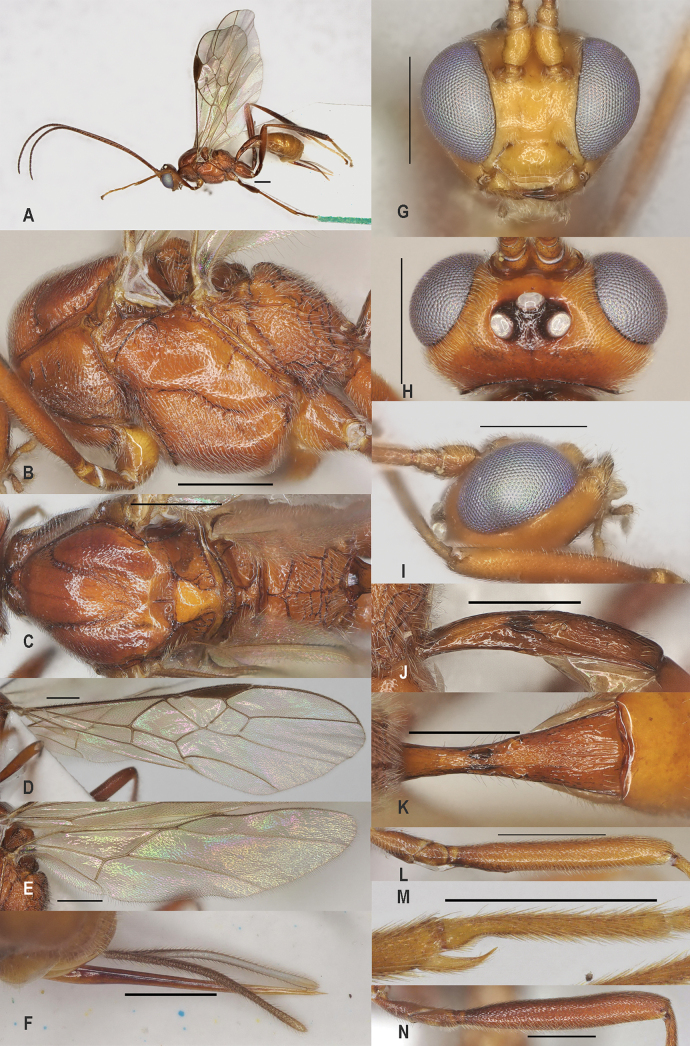
*Zeleaquilus* sp. nov., holotype, ♀ A. Habitus, lateral aspect; B. Mesosoma, lateral aspect; C. Mesosoma, dorsal aspect; D. Fore wing; E. Hind wing; F. Ovipositor sheath; G. Head, anterior aspect; H. Head, dorsal aspect; I. Head, lateral aspect; J. First metasomal tergite, lateral aspect; K. First metasomal tergite, dorsal aspect; L. Fore femur, lateral aspect; M. Fore tibial spur and fore basitarsus; N. Hind femur, lateral aspect. Scale bars: 500 μm.

Metanotum with enlarged smooth knob medio-posteriorly and median carina in front of it short (Fig. 4C); precoxal sulcus sparsely sculptured anteriorly and mesopleuron shiny (Fig. 4B); vein 1r-m of hind wing 1.8–2.2× longer than vein 1-M (Fig. 4E); pterostigma of ♀ dark brown (Fig. 4D); length of malar space 0.2× basal width of mandible; first tergite 2.3–2.6× longer than its apical width; dorsope of first tergite medium-sized (Fig. 4K), but space between dorsope wider than dorsope; hind tarsus mainly white; ovipositor sheath 0.25–0.33× as long as fore wing; first tergite costate medio-posteriorly (Fig. 4K).

##### Comparative diagnosis.

Very similar to *Z.inclinator* but differs mainly by the large ocelli (smaller in *Z.inclinator*), the white hind tarsus (yellowish in *Z.inclinator*) and the blackish brown pterostigma (pale yellow in *Z.inclinator*).

##### Description.

Holotype, ♀, length of fore wing 5.1 mm, of body 4.5 mm, and antenna 1.2× as long as fore wing.

***Head*.** Antennal segments 34, third segment 1.1× longer than fourth segment and third, fourth and penultimate segments 3.6×, 3.4×, and 2.0× longer than wide, respectively; length of maxillary palp 1.3× longer than height of head; frons smooth and behind antennal sockets distinctly impressed; POL: diameter of posterior ocellus: OOL = 7: 5: 5; vertex superficially finely punctulate and densely setose (Fig. 4H); clypeus strongly convex in lateral view, distinctly punctate and with long setae (Fig. 4I); face superficially finely punctate and distinctly narrowed ventrally, minimum width of face 1.2× height of face (Fig. 4G); length of eye 2.5× temple in dorsal view (Fig. 4H); length of malar space 0.2× basal width of mandible.

***Mesosoma*.** Length of mesosoma 1.5× its height; dorsally side of pronotum shiny and smooth, medially punctate and with three carinae and posteriorly largely smooth except crenulate posterior border; prepectal carina no-lamelliform; precoxal sulcus dorsally distinctly crenulate and anteriorly broadly rather superficially punctate and shiny; remainder of mesopleuron smooth and shiny but antero-dorsally punctate (Fig. 4B); mesosternum largely smooth and shiny; metapleuron spaced rugose; mesoscutal lobes superficially punctate or punctulate, interspaces smooth and shiny; scutellar sulcus deep anteriorly, with one long and four short carinae; scutellum slightly convex, and punctulate, its medio-posterior depression rather wide and laterally crenulate; metanotum with large posterior knob and with a pair of rather short carinae medially; propodeum completely areolate, remotely rugose, subbasal carina complete, angulate, medio-longitudinal carina complete, and mostly weakly developed; in lateral view propodeum gradually lowered posteriorly except for dorsal part in front of subbasal carina, dorsal part comparatively large, mainly smooth and shiny (Fig. 4B, C).

***Wings*.** Fore wing (Fig. 4D): r:3-SR:SR1 = 9:29:134; 2-SR:3-SR: r-m = 37:29:23; 1-CU1:2-CU1 = 2:28; cu-a vertical, postfurcal. Hind wing (Fig. 4E): r absent; M+CU:1-M = 70:22; 1r-m 1.8× 1-M.

***Legs*.** Hind coxa largely smooth dorsally; length of fore femur 7.5× its width (Fig. 4L); length of fore tibial spur 0.3× fore basitarsus (Fig. 4M); lengths of hind femur and basitarsus 7.0× and 9.2× their widths, respectively (Fig. 4N).

***Metasoma*.** First tergite 2.6× longer than its apical width, part behind level of spiracles distinctly widened and rugose but posteriorly striate, dorsope elliptical and medium-sized, area behind it depressed (Fig. 4K), laterope medium-sized and sublateral (Fig. 4J); second tergite glabrous, smooth and strongly shiny; ovipositor comparatively slender basally; length of ovipositor sheath 0.22× as long as fore wing and sheath with semi-erect, medium-sized setae (Fig. 4A, F).

***Colour*.** Body rather dark reddish brown, but head (except posteriorly), scutellum, and metasoma (except first tergite) brownish yellow; palpi and tegulae pale yellowish basally but palpi largely whitish, hind tarsus largely white, but telotarsi dorsally and base of basitarsus brownish; veins and pterostigma dark brown; wings subhyaline with slight infuscation; apex of ovipositor sheath brown.

***Variation*.** Vein 1r-m of hind wing 1.8–2.2× as long as vein 1-M; fore femur of ♀ 7.0–7.5× longer than wide; hind femur of ♀ 6.0–7.0× longer than wide; first metasomal tergite 2.3–2.6× its apical width. Antennal segments of ♀ 34(1), 35(2); of ♂ unknown.

##### Distribution.

China (Shaanxi).

##### Biology.

Unknown.

##### Etymology.

Named after the blackish brown pterostigma; *aquilus* is Latin for blackish.

#### ﻿ Zele caligatus

Taxon classificationAnimaliaHymenopteraBraconidae

(Haliday, 1835)

6EF8455A-BA4A-5D27-96C8-F1EF28955ECF

Meteorus caligatus Haliday, 1835: 25.
Meteorus neesii Ruthe, 1862: 22, 23. Syn. by Schmiedeknecht 1897.
Dyscoletes alaskensis Ashmead, 1902: 247. Syn. by van Achterberg 1979.
Meteorus caligatus var. sibiricus : Fahringer 1929: 8. Syn. by van Achterberg 1979.
Zele caligatus : van Achterberg 1984: 110; Chen et al. 1987: 94.

##### Type material examined.

***Lectotype*** of *Zelecaligatus.* Ireland • ♀; Dublin, Jullymore; Haliday leg.; (NMI Dublin).

##### Other material examined.

China – **Hubei Prov.** • 1 ♀; Xingshan, Longmen River; alt. 1300 m; 9 May 1994; You-wei Zhang leg.; light trap; (ZJUH) No. 135714.

##### Diagnosis.

Precoxal sulcus narrowly sculptured medially; basal 1/2 of propodeum largely smooth and shiny (except slightly curved subbasal carina); first metasomal tergite 1.7–2.0× longer than its apical width; dorsope medium-sized and space between dorsope ~2.0× width of dorsope; ovipositor sheath 0.19–0.28× as long as fore wing.

##### Distribution.

China (Anhui, Gansu, Hebei, Heilongjiang, Hubei, Jilin, Liaoning, Ningxia, Shanxi, Xinjiang, Zhejiang), Finland, France, Germany, Ireland, Italy, Japan, Netherlands, Norway, Poland, Russia, Sweden, Switzerland, United Kingdom.

##### Biology.

Reared from Geometridae and Nymphalidae.

#### ﻿ Zele carinatus

Taxon classificationAnimaliaHymenopteraBraconidae

Fang, van Achterberg & Chen sp. nov.

EC29CA9C-F294-5E4D-AB97-9F4E29AEE212

https://zoobank.org/7CF5797F-FB6E-47DD-B301-EBF24245C1D9

Fig. 5

##### Type material.

***Holotype***. China – **Sichuan Prov.** • ♀; Ganzizangzu Zizhizhou, Luding, Moxi; 18 Jun. 2005; Jing-xian Liu leg.; (ZJUH) No. 202401086. GenBank accession no. PV356325.

##### Diagnosis.

**Figure 5.**
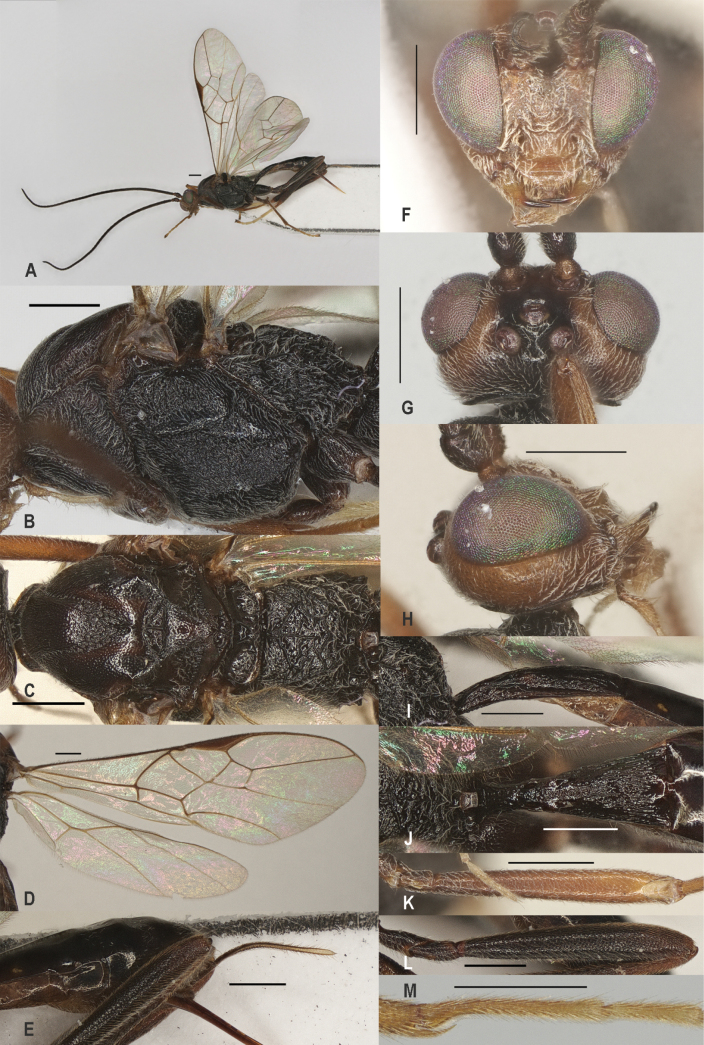
*Zelecarinatus* sp. nov., holotype, ♀ A. Habitus, lateral aspect; B. Mesosoma, lateral aspect; C. Mesosoma, dorsal aspect; D. Wings; E. Ovipositor sheath; F. Head, anterior aspect; G. Head, dorsal aspect; H. Head, lateral aspect; I. First metasomal tergite, lateral aspect; J. First metasomal tergite, dorsal aspect; K. Fore femur, lateral aspect; L. Fore tibial spur and fore basitarsus; M. Hind femur, lateral aspect. Scale bars: 500 μm.

Pterostigma dark brown or brown (Fig. 5D) and mandible yellow (except dark apex) (Fig. 5F); subbasal carina of propodeum distinctly oblique medially (Fig. 5C); propodeum depressed posteriorly and slightly shorter in lateral view (Fig. 5B); mesoscutum medio-posteriorly with short longitudinal carina (Fig. 5C); first tergite ~2.3× longer than its apical width; dorsope of first tergite rather small and space between dorsope much wider than dorsope and sculptured (Fig. 5J); hind tarsus mainly white; ovipositor sheath ~0.23× as long as fore wing; vein r of fore wing oblique.

##### Comparative diagnosis.

Very similar to *Z.rugulosus* but differs mainly by the blackish brown pterostigma (pale yellow in *Z.rugulosus*), the distinctly oblique subbasal carina of propodeum (transverse in *Z.rugulosus*) and the short longitudinal carina on the mesoscutum (long in *Z.rugulosus*).

##### Description.

Holotype, ♀, length of fore wing 6.9 mm, of body 6.7 mm, and antenna 1.1× as long as fore wing.

***Head*.** Antennal segments 42, third segment nearly as long as fourth segment and third, fourth and penultimate segments 2.8×, 2.7×, and 2.0× longer than wide, respectively; length of maxillary palp 1.5× longer than height of head; frons smooth and behind antennal sockets impressed; POL: diameter of posterior ocellus: OOL = 8: 6: 6; vertex less convex, smooth and densely setose (Fig. 5G); clypeus rather convex in lateral view, widely smooth, only punctate medially; face largely crenulate and matt, widened ventrally, minimum width of face 1.2× height of face (Fig. 5F); length of eye 1.8× temple in dorsal view (Fig. 5G); length of malar space 0.3× basal width of mandible.

***Mesosoma*.** Length of mesosoma 1.6× its height; side of pronotum striate ventrally, rugulose-crenulate medially, smooth postero-dorsally; epicnemial area smooth; precoxal sulcus densely rugose, only posteriorly narrowly smooth; dorsal of mesopleuron largely smooth with indistinct punctulation (Fig. 5B); mesosternum finely punctulate dorsally; metapleuron mainly smooth and matt, ventrally rugose; mesoscutal lobes finely punctulate and shiny; notauli anteriorly finely and narrowly crenulate, posteriorly narrowly crenulate-rugose; mesoscutum medio-posteriorly with a short carina; scutellar sulcus deep and rather wide with one long obvious median carina; scutellum rather convex and punctulate; metanotum with small and smooth knob medio-posteriorly, one short media carina on knob, but without median carina in front of it; propodeum widely reticulate-rugose, subbasal carina of propodeum straight and oblique medially, comparatively inconspicuously rugulose anteriorly; propodeum with long straight median carina, rather convex and short in lateral view; in lateral view propodeum gradually lowered posteriorly, posterior of it not separated from antero-dorsal part distinctly (Fig. 5B, C).

***Wings*.** Fore wing (Fig. 5D): r:3-SR:SR1 = 10:20:100; 2-SR:3-SR: r-m = 26:25:17; 1-CU1:2-CU1 = 3:75; cu-a vertical, postfurcal. Hind wing (Fig. 5D): r absent; M+CU:1-M = 75:15; 1r-m 2.6× 1-M.

***Legs*.** Hind coxa densely punctate dorsally; length of fore femur 7.6× its width (Fig. 5K); length of fore tibial spur 0.3× fore basitarsus (Fig. 5M); lengths of hind femur and basitarsus 7.2× and 9.2× their widths, respectively (Fig. 5L).

***Metasoma*.** First tergite 2.3× longer than its apical width, its surface irregularly rugulose; dorsope elliptical and comparatively small (Fig. 5J), laterope comparatively small and very narrow (Fig. 5I); second tergite mainly bare and smooth; ovipositor comparatively slender basally; length of ovipositor sheath 0.23× as long as fore wing, sheath with rather slanted and long setae (Fig. 5E).

***Colour*.** Antenna, mesosoma, coxae of all legs, hind leg (except hind tarsus) and metasoma, largely black; head, fore and middle legs mainly reddish brown; hind tibia largely black but its apical 1/5 reddish brown; hind tarsus largely whitish yellow, but its telotarsi dorsally and base of basitarsus brown; palpi pale yellow; vein C+SC+R of fore wings, pterostigma and ovipositor sheath (except pale brown apex) dark brown.

##### Distribution.

China (Sichuan).

##### Biology.

Unknown.

##### Etymology.

Named after the long straight median carina of the propodeum; *carinatus* is Latin for ridge.

#### ﻿ Zele chinensis

Taxon classificationAnimaliaHymenopteraBraconidae

Chen & He, 1997

7885C1E9-C6FC-5241-B58B-3F9DCB9EBD5D

Fig. 6

Zele chinensis Chen & He, 1997: 1655.

##### Type material examined.

***Holotype*.** China – **Sichuan Prov.** • ♀; Fengdu, Shiping; 6 Oct. 1994; Jian Yao leg.; (ZJUH) No. 149944.

##### Other material examined.

China – **Beijing Prov.** • 1 ♀; Dajuesi; 14 Sept. 1980; light trap; (ZJUH) No. 200012461. – **Guandong Prov.** • 1 ♂; Shaoguan, Ruyuan, Yaozu Zizhixian, Nanling; 10 Oct. 2004; Zai-fu Xu leg.; (ZJUH) No. 202401071. – Fujian Prov. • 1 ♀; Dehua, Mt. Jiuxian; alt. 1400 m; 12 Sept. 2002; Xiao-xia Yu leg.; (ZJUH) No. 20024939. – **Zhejiang Prov.** • 1 ♀; Anji, Mt. Longwang; 31 Aug. 1993; Xiao-xia Yu leg.; (ZJUH) No. 9310776. • 1 ♀; Longquan, Mt. Fengyang; 22 Aug. 1982; Yu-lin Wu leg.; (ZJUH) No. 202315024. GenBank accession no. PV356320.

##### Diagnosis.

**Figure 6.**
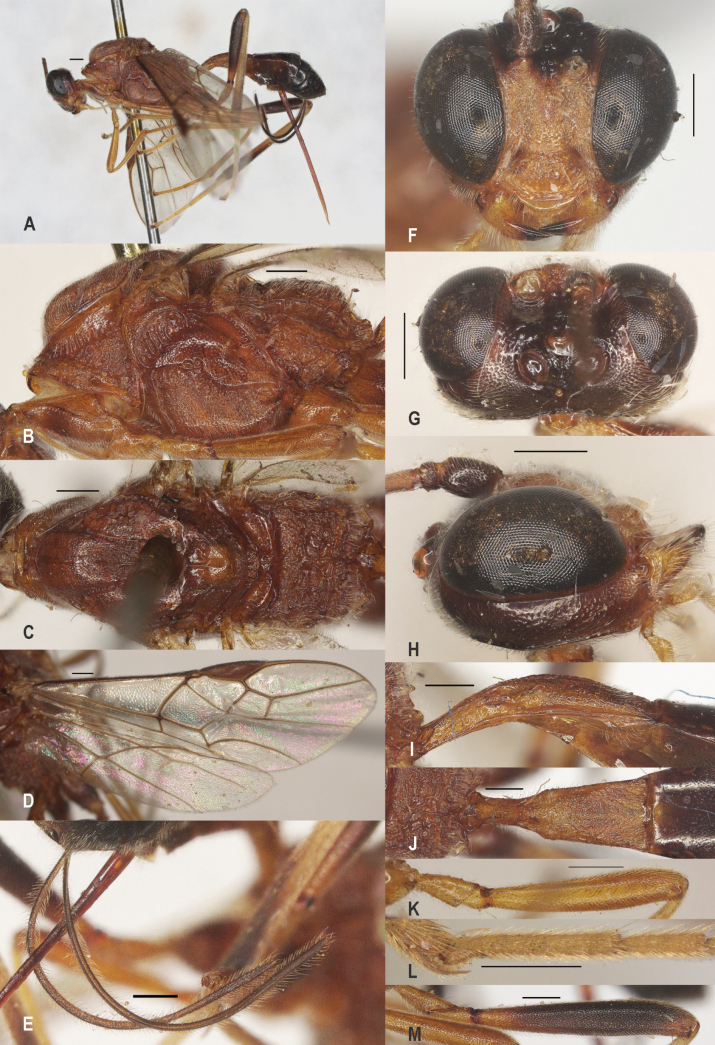
*Zelechinensis* Chen & He, holotype, ♀ A. Habitus, lateral aspect; B. Mesosoma, lateral aspect; C. Mesosoma, dorsal aspect; D. Wings; E. Ovipositor sheath; F. Head, anterior aspect; G. Head, dorsal aspect; H. Head, lateral aspect; I. First metasomal tergite, lateral aspect; J. First metasomal tergite, dorsal aspect; K. Fore femur, lateral aspect; L. Fore tibial spur and fore basitarsus; M. Hind femur, lateral aspect. Scale bars: 500 μm.

Head in dorsal view black or largely so and contrasting with reddish brown mesosoma (Fig. 6C, G); scutellar sulcus shallow antero-laterally; apical 1/2 of hind tibia dark brown; middle of hind femur and apex of hind tibia dark brown (Fig. 6A, M); area in front of lateral ocellus only finely punctate; ocelli comparatively larger (Fig. 6G); first tergite 2.4–2.6× longer than its apical width; dorsope of first tergite large and space between dorsope somewhat wider than dorsope and sculptured (Fig. 6J); hind tarsus mainly ivory; ovipositor sheath 0.48–0.64× as long as fore wing.

##### Distribution.

China (Beijing, Fujian, Guangdong, Sichuan, Zhejiang).

##### Biology.

Unknown.

#### ﻿ Zele chlorophthalmus

Taxon classificationAnimaliaHymenopteraBraconidae

(Spinola, 1808)

22928760-A14B-56FD-BDB0-D5DCB7235E7D

Fig. 7

Bracon chlorophthalmus Spinola, 1808: 133–134.
Ichneumon nudator Thunberg, 1822: 263. Syn. by van Achterberg 1979.
Meteorus splendens Costa, 1884: 171. Syn. by van Achterberg 1979.
Meteorus (Zemiotes) nigricollis Thomson, 1895: 2150. Syn. by van Achterberg 1979.
Zele chlorphthalmus : van Achterberg 1979: 370; Chen et al. 1987: 94.

##### Type material examined.

***Neotype*** of *Braconchlorophthalmus.* Netherlands • ♀; Asperen; 8 Sep. 1972; C. J. Zwakhals leg (RMNH).

##### Other material examined.

China – **Anhui Prov.** • 1 ♀; Yuexi; 22 Sept. 1981; Fu-an Yang leg.; (ZJUH) No. 820518. – **Guizhou Prov.** • 1 ♀; Mayanghe Wanjia; 27–30 Sept. 2007; Jing-xian Liu leg.; (ZJUH) No. 200708481. – **Hebei Prov.** • 1 ♀; Handan; 19 Sept. 1977; Zhong-shi Ma leg., light trap; (ZJUH) No. 801484. – **Heilongjiang Prov.** • 1 ♀; Harbin; 27 Jun.–15 Jul. 1957; San-yang Fang leg.; (ZJUH) No. 948187–948191, 948193–948197. • 2 ♀♀; Harbin; 1 Aug. 1977; Jun-hua He leg.; light trap; (ZJUH) Nos. 771651, 771521. • 1 ♀; Harbin; 28 Jul. 1982; Lv-hong Zhang leg.; (ZJUH) No. 948208. • 4 ♂♂; Harbin; 25 Jul. 1983; Lv-hong Zhang leg.; (ZJUH) No. 947130 (the four have the same number). • 3 ♀♀, 7 ♂♂; Harbin; 21–26 Jul. 1984; Lv-hong Zhang leg.; (ZJUH) Nos. 947102–947111. • 4 ♀♀, 4 ♂♂; Keshan; 5 May 1983; Lv-hong Zhang leg.; (ZJUH) Nos. 947093–947099, 947101. – **Jilin Prov.** • 1 ♀; Mt. Changbai; 9 Aug. 1977; Jun-hua He leg.; light trap; (ZJUH) No. 770572. – **Liaoning Prov.** • 1 ♀; Shenyang; 21 Aug. 1977; Jun-hua He leg.; light trap; (ZJUH) No. 771209. • 1 ♀; Shenyang, Dongjun; 29 Jun. 1984; Li-min Sun leg.; (ZJUH) No. 948278. • 4 ♀♀; Shenyang, Dongjun; Jul. 1984; Li-min Sun leg.; (ZJUH) Nos. 948302 to 948305. – **Nei Mongol Zizhiqu** • 1 ♀; Wuqian; 8 Jul. 1978; Ji-Kun Yang leg.; (ZJUH) No. 200012252. • 1 ♂; Mt. Helan; 6 Aug. 2007; Bo Qiu leg.; (ZJUH) No. 202401078. • 1 ♀; Zhengxiangbaiqi Grassland; 30 Aug. 1998; Yuan-chao Guo leg.; (ZJUH) No. 200010349. – **Ningxia Huizu Zizhiqu** • 1 ♀; Yinchuan; 8 Sept. 1983; Wen-zhong Xu leg.; (ZJUH) No. 840999. – **Sichuan Prov.** • 1 ♂; Mt. Qingcheng; 21 Jun. 1987; Gang Chen leg.; (ZJUH) No. 200012347. – **Xinjiang Uygur Zizhiqu** • 1 ♀; Aksu; 14 Jun. 1983; Unknown leg.; (ZJUH) No. 860035. • 1 ♀; Bole; Jun.–Sept. 1982; Ya-qiong Chen leg.; (ZJUH) No. 826465. • 1 ♀; Lilong; 2 Aug. 1981; De-fu He leg.; (ZJUH) No. 820005. • 1 ♀; Yili, Xinyuan; 19 Jul. 2005; Hong-ying Zhang leg.; (ZJUH) No. 201912971. • 1 ♀; Yumin Tast; 16 Jul. 2005; Hong-ying Zhang leg.; (ZJUH) No. 201705529. – **Zhejiang Prov.** • 2 ♀♀; Qingyuan, Baishanzu; 18–19 Jul. 1994; Hong Wu leg.; (ZJUH) Nos. 948559, 945955. GenBank accession no. PV356322.

##### Diagnosis.

**Figure 7.**
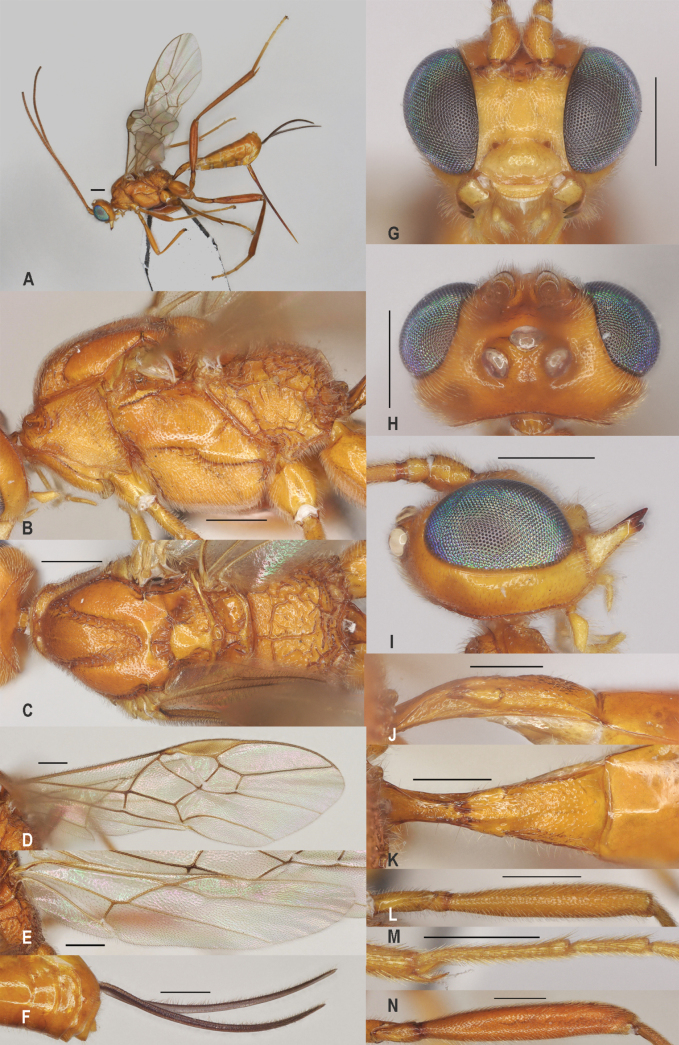
*Zelechlorophthalmus* (Spinola), China, Guizhou, ♀ A. Habitus, lateral aspect; B. Mesosoma, lateral aspect; C. Mesosoma, dorsal aspect; D. Fore wing; E. Hind wing; F. Ovipositor sheath; G. Head, anterior aspect; H. Head, dorsal aspect; I. Head, lateral aspect; J. First metasomal tergite, lateral aspect; K. First metasomal tergite, dorsal aspect; L. Fore femur, lateral aspect; M. Fore tibial spur and fore basitarsus; N. Hind femur, lateral aspect. Scale bars: 500 μm.

Pterostigma of both sexes yellowish, laterally more or less darkened (Fig. 7D); length of first metasomal tergite 2.1–2.4× its apical width and moderately narrowed in front of dorsope (Fig. 7J, K); hind tarsus yellowish medially, similar to apex of hind tibia or nearly so (Fig. 7A); dorsope of first tergite large and space between dorsope approximately as wide as dorsope (Fig. 7K); ovipositor sheath 0.41–0.53× as long as fore wing.

##### Distribution.

Armenia, Austria, Azerbaijan, Belgium, Bulgaria, Cape Verde Islands, China (Anhui, Gansu, Guizhou, Hebei, Heilongjiang, Hubei, Jiangsu, Jilin, Liaoning, Nei Mongol, Ningxia, Xinjiang, Sichuan, Zhejiang), Croatia, Cyprus, Czech Republic, Denmark, Egypt, Finland, France, Georgia, Germany, Hungary, India, Iran, Italy (including Sardinia and Sicily), Lithuania, Moldova, Netherlands, North Macedonia, Norway, Poland, Romania, Russia, Slovakia, Spain, Sweden, Switzerland, U.K.

##### Biology.

Parasitoids of Arctiidae, Geometridae, Lasiocampidae, Limacodidae, Lymantriidae, Noctuidae, Pyralidae, Tortricidae and Zygaenidae.

#### ﻿ Zele confusus

Taxon classificationAnimaliaHymenopteraBraconidae

Fang, van Achterberg & Chen sp. nov.

E97DFD59-4032-51A3-85C0-43BC2D320867

https://zoobank.org/1A115FD7-C145-426A-B181-E49CF8494E07

Fig. 8

##### Type material.

***Holotype*.** China – **Yunnan Prov.** • ♀; Yinjiang; 22 May 2009; Su-jiong Zhang leg.; (ZJUH) No. 202401050. GenBank accession no. PV356300.

##### Diagnosis.

**Figure 8.**
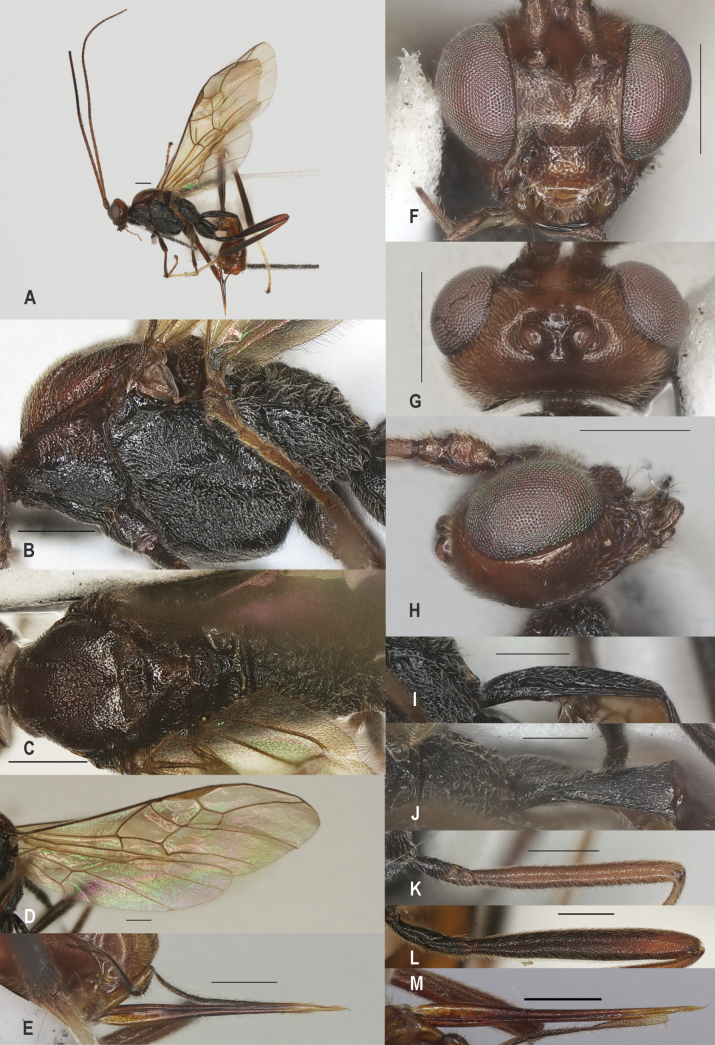
*Zeleconfusus* sp. nov., holotype, ♀ A. Habitus, lateral aspect; B. Mesosoma, lateral aspect; C. Mesosoma, dorsal aspect; D. Wings; E. Ovipositor sheath; F. Head, anterior aspect; G. Head, dorsal aspect; H. Head, lateral aspect; I. First metasomal tergite, lateral aspect; J. First metasomal tergite, dorsal aspect; K. Fore femur, lateral aspect; L. Hind femur, lateral aspect; M. Ovipositor. Scale bars: 500 μm.

Hind femur slender, and basal part of ovipositor comparatively robust, maximum width of basal part of ovipositor 0.6× maximum width of hind femur (Fig. 8L, M); subbasal transverse carina of propodeum well developed, different from surrounding sculpture; fore femur slender, ~9.5× as long as wide (Fig. 8K); hind femur largely black only apical brown and hind tibia (except basal 1/3) black (Fig. 8A); hind tarsus mainly white; first tergite ~2.5× longer than its apical width, moderately shiny and with comparatively small dorsope (Fig. 8J) with space between dorsope much wider than dorsope; ovipositor sheath ~0.23× as long as fore wing.

##### Comparative diagnosis.

Very similar to *Z.cristatus* but differs mainly by the more developed malar space (narrow in *Z.cristatus*), wider face, smaller eyes in dorsal view and the darker mesosoma (especially scutellum usually dark reddish in *Z.cristatus*).

##### Description.

Holotype, ♀, length of fore wing 6.2 mm, of body 6.2 mm, and antenna 1.2× as long as fore wing.

***Head*.** Antennal segments 39, third segment 0.9× shorter than fourth segment and third, fourth and penultimate segments 3.3×, 3.3×, and 2.0× longer than wide, respectively; length of maxillary palp 2.1× longer than height of head; frons smooth and behind antennal sockets impressed; POL: diameter of posterior ocellus: OOL = 13: 9: 8; vertex convex, punctulate and densely setose (Fig. 8G); clypeus rather convex in lateral view, widely punctate (Fig. 8H); face mainly punctulate, it widened ventrally, minimum width of face 1.4× height of face (Fig. 8F); length of eye 1.8× temple in dorsal view (Fig. 8G); length of malar space 0.5× basal width of mandible.

***Mesosoma*.** Length of mesosoma 1.6× its height; side of pronotum densely reticulate-rugose ventrally and posteriorly, sulcate anteriorly; epicnemial area punctulate anteriorly, punctulate-rugose postero-dorsally; precoxal sulcus dorsally narrowly crenulate, densely rugose-reticulate dorsally; dorsal of mesopleuron coarsely punctate, narrowly smooth (Fig. 8B); mesosternum punctulate; metapleuron widely smooth but near coxa rugulose; mesoscutal lobes densely punctate; notauli finely and narrowly crenulate, mesoscutum medio-posteriorly widely crenulate-rugose and no carina; scutellar sulcus deep and wide with one long median carina; scutellum rather convex and finely punctulate; metanotum with small smooth knob medio-posteriorly and without median carina in front of it; propodeum reticulate-rugose, subbasal transverse carina of propodeum mainly straight to curved posteriad, area comparatively reticulate-punctate; in lateral view propodeum gradually lowered posteriorly, posterior part not distinctly separated from antero-dorsal part (Fig. 8B, C).

***Wings*.** Fore wing (Fig. 8D): r:3-SR:SR1 = 13:20:98; 2-SR:3-SR: r-m = 20:17:13; 1-CU1:2-CU1 = 5:62; cu-a vertical, postfurcal. Hind wing (Fig. 8D): r absent; M+CU:1-M = 62:12; 1r-m 2.1× 1-M.

***Legs*.** Hind coxa densely punctate dorsally; length of fore femur 9.5× its width (Fig. 8K); length of fore tibial spur 0.4× fore basitarsus; lengths of hind femur and basitarsus 7.5× and 10.3× their widths, respectively (Fig. 8L).

***Metasoma*.** First tergite 2.5× longer than its apical width, its surface rugulose anteriorly, irregular and obvious rugose behind spiracles; dorsope elliptical and comparatively small, area in front of them depressed (Fig. 8J), laterope comparatively small (Fig. 8I); second tergite mainly bare, smooth; ovipositor comparatively robust basally; length of ovipositor sheath 0.23× as long as fore wing, sheath with short semi-erect setae (Fig. 8E).

***Colour*.** Mesosoma (but side of pronotum reddish brown), coxae and trochanters of all legs and first metasomal tergite largely black; head, antenna, fore and middle legs, all metasoma (except first tergite) mainly reddish brown; hind femur largely black except the reddish brown basal 1/3; hind tibia nearly black except reddish brown apical 1/3; hind tarsus white; veins, pterostigma and ovipositor sheath (except pale brown apex) brown; wings subhyaline with largely infuscation.

##### Distribution.

China (Yunnan).

##### Biology.

Unknown.

##### Etymology.

Named after the confusing morphology of the species; *confusus* is Latin for confused.

#### ﻿ Zele cristatus

Taxon classificationAnimaliaHymenopteraBraconidae

Fang, van Achterberg & Chen sp. nov.

598EEC65-0EDD-5C88-A16B-CAC2CA9E876F

https://zoobank.org/648274E1-13BE-4D98-A20E-A4CC553A0B7F

Fig. 9

##### Type material.

***Holotype*.** China – **Zhejiang Prov.** • ♀; Hangzhou, Mt. Tianmu; 2 Jul. 2010; Jiang-li Tan leg.; (ZJUH) No. 202401046. ***Paratypes*.** China – **Anhui Prov.** • 1 ♀; Yuexi; 21 Sep. 1981; Fu-an Yang leg.; (ZJUH) No. 820519. – **Fujian Prov.** • 1 ♀; Guadun; 2 Jul. 1982; Shi-cheng Qi leg.; (ZJUH) No. 202315031. – **Guangdong Prov.** • 1 ♀; Shaoguan, Nanling Nature Reserve; 1 Oct. 2004; Zai-fu Xu leg.; (ZJUH) No. 202401055. – **Guizhou Prov.** • 1 ♀; Dashahe; 22 Aug. 2004; Qiong Wu leg.; light trap; (ZJUH) No. 200617535. • 1 ♀; Kuankuoshui Reserve; 6–8 Jun. 2004; Jiang-li Tan leg.; (ZJUH) No. 201005567. – **Hubei Prov.** • 1 ♀; Shennongjia, Songbai; 19 Jul. 1997; Yu-zhou Du leg.; (ZJUH) No. 975178. • 1 ♀; Huanggang, Mt. Dabie, Mt. Wujia Scenic Spot; 30 Jun. 2014; Zhen Liu leg.; (ZJUH) No. 202401056. – **Hunan Prov.** • 1 ♀; Shimen, Mt. Huping, Sanhecun; 11 Jul. 2009; Li Ma leg.; (ZJUH) No. 200901933. – **Shaanxi Prov.** • 1 ♀; Baoji, Fengxian, Huangniupu; 20 Aug. 2013; Bin-bin Tu leg.; (ZJUH) No. 201308574. – **Sichuan Prov.** • 1 ♀; Yajiang; 14 Jun. 1996; Yu-zhou Du leg.; (ZJUH) No. 977673. – **Yunnan Prov.** • 1 ♀; Kunming; 18 May 1981; Jun-hua He leg.; (ZJUH) No. 814731. – **Zhejiang Prov.** • 2 ♀♀; Anji; 11 Jul. 1991; Jun-hua He leg.; (ZJUH) Nos. 915978, 915980. • 1 ♀; Mt. Xitianmu; 8 Oct. 1982; Kun-yan Zhu leg.; (ZJUH) No. 825943. • 2 ♀♀; same locality as for preceding; 4 Jun. 1994; Xue-xin Chen leg.; (ZJUH) Nos. 941882, 941883. • 1 ♀; same locality as for preceding; 6 Jul. 1994; Jun-hua He leg.; (ZJUH) No. 965027. • 1 ♀; Mt. Tianmu; 23 Jun. 1984; Tao-liang Zhu leg.; (ZJUH) No. 842003. • 1 ♀; same locality as for preceding; 21 Jul. 1987; Xue-xin Chen leg.; (ZJUH) No. 873539. • 1 ♀; Hangzhou, Mt. Xitianmu, Sanmuping; 7 Jul. 1998; Ming-shui Zhao leg.; (ZJUH) No. 20003596. • 3 ♀♀; Hangzhou, Mt. Xitianmu, Xianrending; 29 Jul. 1998; Ming-shui Zhao leg.; light trap; (ZJUH) Nos. 993181, 993182, 993621. • 1 ♂; same locality and collector as for preceding; 10 Aug. 1998; light trap; (ZJUH) No. 997225. • 1 ♀; same locality and collector as for preceding; 27 May 1999; light trap; (ZJUH) No. 996029. • 1 ♀; same locality and collector as for preceding; 3 Jul. 1999; light trap; (ZJUH) No. 996527. • 1 ♀; same locality and collector as for preceding; 20 Jul. 1998; Malaise trap; (ZJUH) No. 992688. • 1 ♀; same locality and collector as for preceding; 20 Jul. 1998; Malaise trap; (ZJUH) No. 992963. • 1 ♀; same locality as for preceding; 20 Jul. 1998; Yang Ma; light trap; (ZJUH) No. 997955. • 1 ♂; same locality as for preceding; 29 Jul. 2003; Qiong Wu leg.; (ZJUH) No. 20039762. • 1 ♀; Hangzhou, Mt. Tianmu, Xianrending; 11 Jul. 1998; Ming-shui Zhao leg.; (ZJUH) No. 200010745. • 4 ♀♀, 1 ♂; same locality as for preceding; alt. 1520 m; 1 Jul. 2001; Mei-hua Piao leg.; (ZJUH) Nos. 200106251, 200106258, 200106260, 200106292, 200106252. • 1 ♀1 ♂; same locality as for preceding; 27 Jul. 2011; Zhen Liu leg.; (ZJUH) Nos. 201101436, 201101446. • 1 ♀; Hangzhou, Mt. Xitianmu, Houshanmen; 3 Nov. 1998; Ming-shui Zhao leg.; light trap; (ZJUH) No. 20000468. • 2 ♀♀; Hangzhou, Mt. Xitianmu, Laodian; 3 Aug. 1998; Ming-shui Zhao leg.; light trap; (ZJUH) Nos. 20001049, 20001050. • 1 ♀; same locality and collector as for preceding; 23 Aug. 1998; light trap; (ZJUH) No. 20001045. • 1 ♀; same locality as for preceding; 10 Aug. 1998; Yun Ma leg.; (ZJUH) No. 20001715. • 4 ♀♀; Qingyuan, Baishanzu; 25 Oct. 1993; Hong Wu leg.; (ZJUH) Nos. 945636, 945849, 945852, 945857. • 1 ♀; Jingning; Aug. 1994; Shu-feng Ye leg.; (ZJUH) No. 943698. • 1 ♀; Mt. Longwang; 20 Oct. 1987; Ying Qian leg.; (ZJUH) No. 87–320. • 1 ♂; Mt. Longwang; 12 May 1996; light trap; (ZJUH) No. 202315032. • 1 ♀; Longquan, Mt. Fengyang, Fengyangjian; 27 Jul. 2007; Jing-xian Liu leg.; (ZJUH) No. 200801351. • 1 ♀; Mt. Longwang, Qianmutian, Bingchuandashigu; 28 Jul. 2011; Sheng-nan Song leg.; (ZJUH) No. 201102536. • 1 ♀; Lishui, Longquan Mt. Fengyang; 12 Dec. 2008; Jin-hua Xu & Li-quan Liu leg.; (ZJUH) No. 201914070. • 1 ♂; Lishui; 15 Oct. 1981; Kun-yan Zhu leg.; (ZJUH) No. 815801. GenBank accession no. PV356297, PV356306, PV356316.

##### Diagnosis.

**Figure 9.**
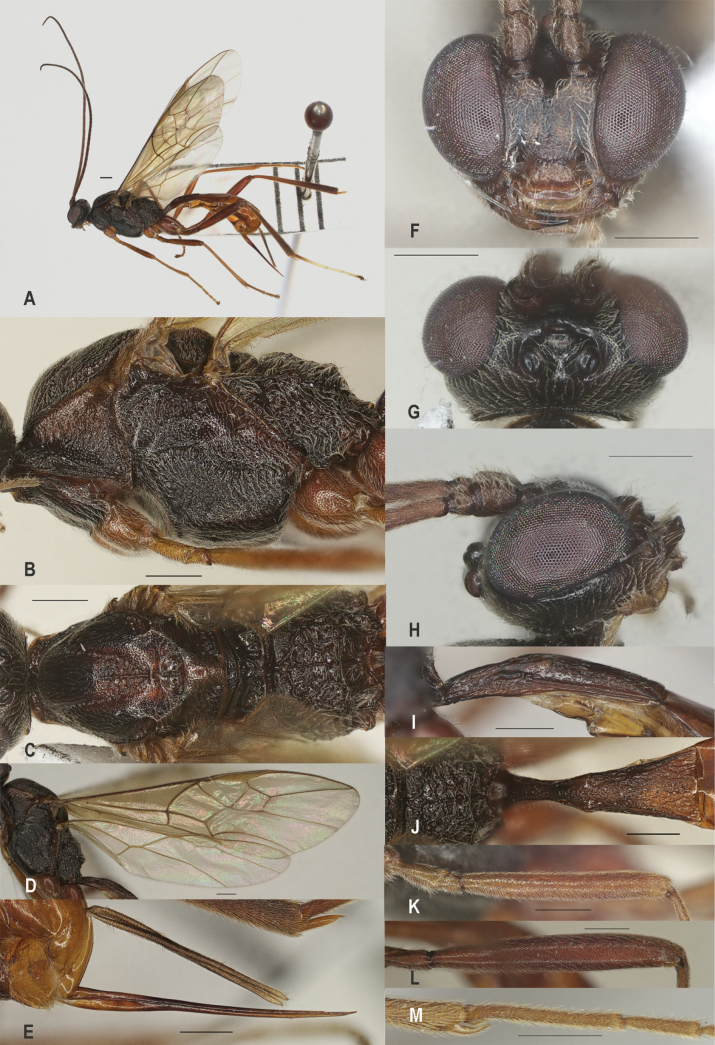
*Zelecristatus* sp. nov., holotype, ♀ A. Habitus, lateral aspect; B. Mesosoma, lateral aspect; C. Mesosoma, dorsal aspect; D. Wings; E. Ovipositor sheath; F. Head, anterior aspect; G. Head, dorsal aspect; H. Head, lateral aspect; I. First metasomal tergite, lateral aspect; J. First metasomal tergite, dorsal aspect; K. Fore femur, lateral aspect; L. Hind femur, lateral aspect; M. Fore tibial spur and fore basitarsus. Scale bars: 500 μm.

First metasomal tergite dark brown or blackish, at most apical 1/3 brown (Fig. 9J) and 2.6–2.7× longer than its apical width; Posterior part of propodeum usually distinctly differentiated from dorsal part; propodeum with irregular subbasal transverse carina and dorsal face of propodeum with more or less developed posterior crest, and dorsally behind transverse carina with distinctly spaced rugae (Fig. 9C); first metasomal tergite dark brown or blackish, at most its apical 1/3 brown (Fig. 9J); dorsope hardly visible in lateral view (and hardly so in lateral view: Fig. 9I), rather small and space between dorsope much wider than dorsope and sculptured (Fig. 9J); hind tarsus mainly whitish; ovipositor sheath ~0.23× as long as fore wing.

##### Comparative diagnosis.

In the past often identified as Z.f.rufulus (Thomson), but Chinese specimens differ in colour (hind tibia apically distinctly contrasting with whitish tarsus vs weakly contrasting in *Z.rufulus*), marginal cell of hind wing (moderately widened vs strongly widened apically), pterostigma (slightly elongate vs not elongate), shape of first tergite (posterior 1/2 less robust vs more robust), clypeus (lower 1/2 mainly below lower level of eyes vs mainly between eyes) and propodeum (often with (or part of) medial crest (lateral view) vs absent or indistinct).

##### Description.

Holotype, ♀, length of fore wing 7.9 mm, of body 8.8 mm, and antenna 1.2× as long as fore wing.

***Head*.** Antennal segments 43, third segment 1.1× longer than fourth segment and third, fourth and penultimate segments 3.9×, 3.3×, and 1.6× longer than wide, respectively; length of maxillary palp incomplete; frons smooth and behind antennal sockets impressed; POL: diameter of posterior ocellus: OOL = 10: 7: 5; vertex convex, punctulate and densely setose (Fig. 9G); clypeus rather convex in lateral view, widely smooth, only punctate medially (Fig. 9H); face punctulate and matt, widened ventrally, minimum width of face 1.1× height of face (Fig. 9F); length of eye 2.6× temple in dorsal view (Fig. 9G); length of malar space 0.3× basal width of mandible.

***Mesosoma*.** Length of mesosoma 1.5× its height; side of pronotum striate-rugose ventrally, reticulate-rugose medially, crenulate and punctate postero-dorsally; epicnemial area widely reticulate-rugose; precoxal sulcus densely reticulate-rugose dorsally, only narrowly punctate ventrally; dorsal of mesopleuron densely double-punctate; (Fig. 9B); mesosternum largely punctulate and shiny; metapleuron anteriorly narrowly striate near pleural suture, mainly smooth and shiny, posteriorly largely rugose; mesoscutal lobes finely punctulate and comparatively shiny; notauli anteriorly widely crenulate, mesoscutum medio-posteriorly narrowly crenulate-rugose with a medium-sized carina; scutellar sulcus deep and comparatively narrow with one long median carina; scutellum slightly convex and finely punctulate; metanotum with three long carinae medially, lateral ones rather converging; propodeum widely reticulate-rugose, subbasal carina of propodeum irregular, comparatively conspicuously rugulose anteriorly, dorsally behind transverse carina distinctly spaced rugose; propodeum with distinctly developed posterior crest, no long median carina, in lateral view gradually lowered posteriorly, posterior part of propodeum usually distinctly differentiated from dorsal part (Fig. 9B, C).

***Wings*.** Fore wing (Fig. 9D): r:3-SR:SR1 = 10:32:130; 2-SR:3-SR: r-m = 31:32:18; 1-CU1:2-CU1 = 3:80; cu-a vertical, postfurcal. Hind wing: r absent; M+CU:1-M = 85:18; 1r-m 2.5× 1-M.

***Legs*.** Hind coxa largely punctulate dorsally; length of fore femur 8.5× its width (Fig. 9K); length of fore tibial spur 0.3× fore basitarsus (Fig. 9M); lengths of hind femur and basitarsus 7.2× and 10.0× their widths, respectively (Fig. 9L).

***Metasoma*.** First tergite 2.6× longer than its apical width, its surface irregularly rugulose; dorsope narrow elliptical and rather small, area in front of dorsope depressed (Fig. 9J), laterope comparatively narrow and deep (Fig. 9I); second tergite mainly bare, smooth; ovipositor comparatively robust basally; length of ovipositor sheath 0.25× as long as fore wing, sheath with moderately erect and short setae (Fig. 9E).

***Colour*.** Antenna, legs (except hind tibia and tarsus) and metasoma (but first tergite somewhere blackish) reddish brown; head and mesosoma largely black; clypeus, mandible, hind tibia (except blackish apical 1/3), veins C+SC+R, 1-M, and vein cu-a of fore wing, ovipositor sheath (except pale yellow apex) dark brown; hind tarsus largely whitish yellow, but its telotarsi dorsally and base of basitarsus dark brown; palpi and pterostigma pale yellowish; wings subhyaline with some infuscation.

***Variation*.** Vein 1r-m of hind wing 2.5–3.0× as long as vein 1-M; fore femur of ♀ 8.0–8.5× longer than wide; hind femur of ♀ 6.9–7.2× longer than wide; first metasomal tergite 2.6–2.7× its apical width. Antennal segments of ♂ 40–44, pterostigma of ♂ dark brown;

##### Distribution.

China (Anhui, Guangdong, Guizhou, Hubei, Hunan, Shaanxi, Sichuan, Yunnan, Zhejiang).

##### Biology.

Unknown.

##### Etymology.

Named after the more or less developed postero-dorsal crest of the propodeum; *cristatus* is Latin for crested.

#### ﻿ Zele curvatus

Taxon classificationAnimaliaHymenopteraBraconidae

Fang, van Achterberg & Chen sp. nov.

2BA72323-CB09-5379-9B30-DC0126438128

https://zoobank.org/115E8B6A-E829-44EC-A4F2-A62FBC7B9F17

Fig. 10

##### Type material.

***Holotype*.** China – **Jilin Prov.** • ♀; Yanbian, Huangsongpu Forest Farm; 4 Oct. 2004; Yu-zhou Du, Zhi-jie Wang leg.; (ZJUH) No. 202401067. GenBank accession no. PV356317.

##### Diagnosis.

**Figure 10.**
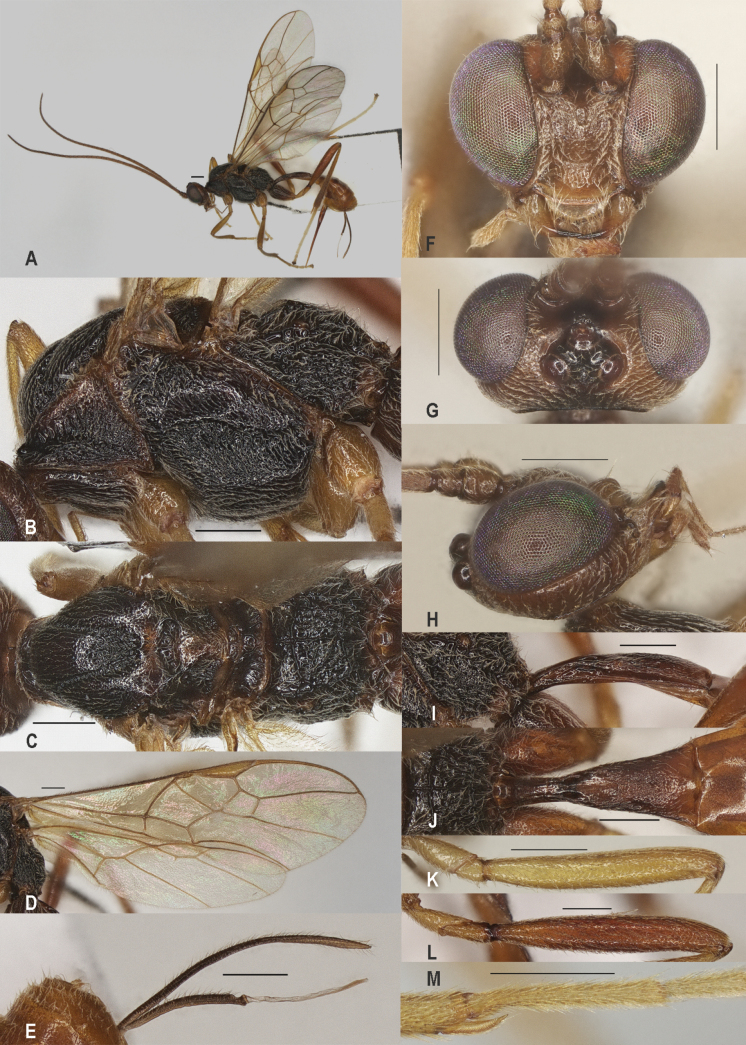
*Zelecurvatus* sp. nov., holotype, ♀ A. Habitus, lateral aspect; B. Mesosoma, lateral aspect; C. Mesosoma, dorsal aspect; D. Wings; E. Ovipositor sheath; F. Head, anterior aspect; G. Head, dorsal aspect; H. Head, lateral aspect; I. First metasomal tergite, lateral aspect; J. First metasomal tergite, dorsal aspect; K. Fore femur, lateral aspect; L. Hind femur, lateral aspect; M. Fore tibial spur and fore basitarsus. Scale bars: 500 μm.

Eyes comparatively large (Fig. 10G), in dorsal view of ♀ ~3.6× as long as temple; first metasomal tergite ~2.3× longer than its apical width and moderately narrowed in front of dorsope; dorsope large and space between dorsope approximately equal to width of dorsope (Fig. 10J); temple directly narrowed and lowered behind eye (Fig. 10G, H); hind tarsus mainly white; ovipositor sheath ~0.30× as long as fore wing; vein r of hind wing vaguely present (Fig. 10D); outline of propodeum rather curved in lateral view (Fig. 10B); subbasal carina of propodeum rather arched and median carina in front of carina comparatively long.

##### Comparative diagnosis.

Very similar to *Z.cristatus* but differs mainly by the large eyes (smaller in *Z.cristatus*), large dorsope and space between dorsope approximately equal to width of dorsope (hardly visible in *Z.cristatus*) and propodeum more curved in lateral view (convex in *Z.cristatus*).

##### Description.

Holotype, ♀, length of fore wing 7.1 mm, of body 7.6 mm, and antenna 1.2× as long as fore wing.

***Head*.** Antennal segments 40, third segment 1.1× longer than fourth segment and third, fourth and penultimate segments 3.4×, 3.2×, and 1.6× longer than wide, respectively; length of maxillary palp 1.4× longer than height of head; frons smooth and behind antennal sockets impressed; POL: diameter of posterior ocellus: OOL = 12: 11: 5; vertex convex, punctulate and densely setose (Fig. 10G); clypeus strongly convex in lateral view, punctate apically (Fig. 10H); face widely smooth but punctulate near antennal sockets, it widened ventrally (Fig. 10F), minimum width of face 1.2× height of face; length of eye 3.6× temple in dorsal view (Fig. 10G); length of malar space 0.2× basal width of mandible.

***Mesosoma*.** Length of mesosoma 1.75× its height; side of pronotum densely striate-rugose ventrally and posteriorly, crenulate medially; epicnemial area rather smooth anteriorly, rugose posteriorly; precoxal sulcus narrowly crenulate dorsally, coarsely reticulate-punctate ventrally; dorsal of mesopleuron smooth and shiny, finely punctulate (Fig. 10B); mesosternum finely punctulate and matt; metapleuron smooth anteriorly but striate-rugose posteriorly; mesoscutal lobes widely punctulate and shiny; notauli distinctly widely crenulate; scutellar sulcus deep and narrow, with one long median carina and two short carinae laterally; scutellum rather convex and weakly punctate; metanotum with small smooth knob medio-posteriorly and without median carina in front of it; propodeum reticulate-rugose, subbasal transverse carina straight to curved, evenly dome-shaped and finely sculptured, medio-longitudinal carina present; in lateral view propodeum gradually lowered posteriorly, posterior of not distinctly separated from antero-dorsal part distinctly (Fig. 10B, C).

***Wings*.** Fore wing (Fig. 10D): r:3-SR:SR1 = 9:16:90; 2-SR:3-SR: r-m = 15:16:13; cu-a vertical, interstitial. Hind wing (Fig. 10D): r present; M+CU:1-M = 60:10; 1r-m 2.5× 1-M.

***Legs*.** Hind coxa largely punctulate dorsally; length of fore femur 7.0× its width (Fig. 10K); length of fore tibial spur 0.3× fore basitarsus (Fig. 10M); lengths of hind femur and basitarsus 6.6× and 11.0× their widths, respectively (Fig. 10L).

***Metasoma*.** First tergite 2.3× longer than its apical width, it narrowed medially, its surface smooth, except some rugulosity behind spiracles; dorsope comparatively large, oval, area dorsope behind depressed (Fig. 10J), laterope comparatively narrow but deep (Fig. 10I); second tergite mainly bare, smooth; ovipositor comparatively robust basally; length of ovipositor sheath 0.30× as long as fore wing, sheath with moderately erect and medium-sized setae (Fig. 10E).

***Colour*.** Head, antenna, hind femur and metasoma (except first metasomal tergite dark brown) brownish yellow; mesosoma black; face, mandible, palpi, fore and middle legs and hind trochanters light brown; hind coxa and basal 1/2 of hind tibia and ovipositor sheath dark brown; hind tarsus white; veins (except dark brown veins C+SC+R, M+CU1, 1-M and cu-a) and pterostigma pale brown.

##### Distribution.

China (Jilin).

##### Biology.

Unknown.

##### Etymology.

Named after the evenly dome-shaped subbasal carina of the propodeum; *curvatus* is Latin for curved.

#### ﻿ Zele curvinervis

Taxon classificationAnimaliaHymenopteraBraconidae

Fang, van Achterberg & Chen sp. nov.

508E3266-99C8-5128-AEFF-02553DB0AD23

https://zoobank.org/06CD158E-01EF-4092-906E-8424171C1AD3

Fig. 11

##### Type material.

***Holotype*.** China – **Sichuan Prov.** • ♀; Ganzizangzu Zizhizhou, Luding, Moxi; 19 Jun. 2005; light trap; (ZJUH) No. 202401058. ***Paratype*.** China – **Qinghai Prov.** • 1 ♀; Yushuzangzu Zizhizhou, Nangqian; 25 Jun. 2005; Jiang-li Tan leg.; (ZJUH) No. 202401048. GenBank accession no. PV356308, PV356298.

##### Diagnosis.

**Figure 11.**
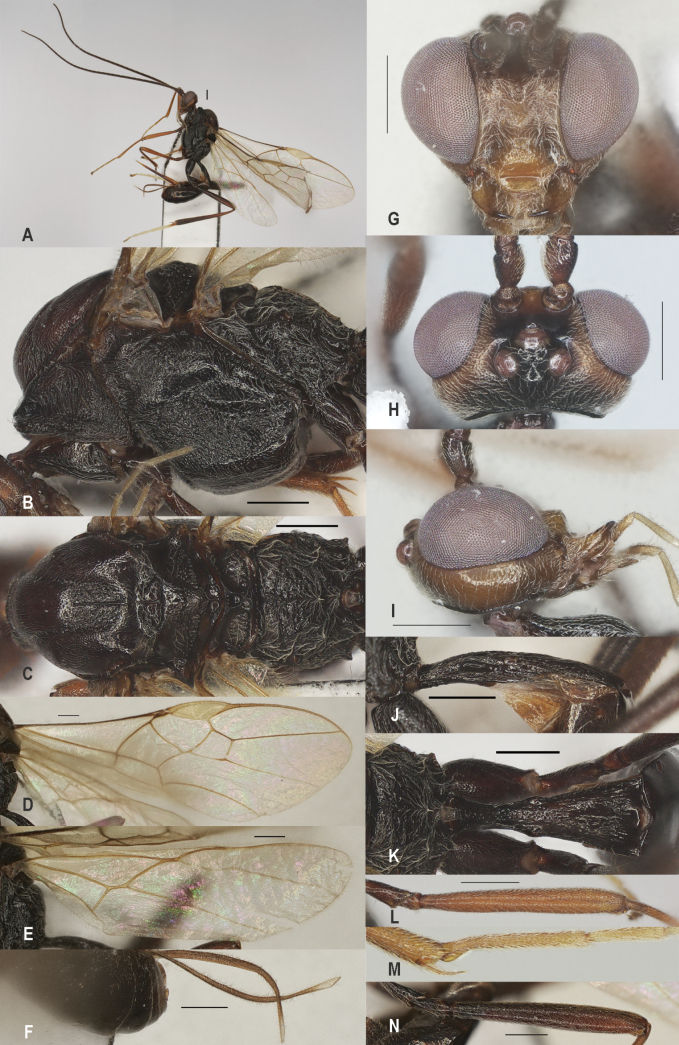
*Zelecurvinervis* sp. nov., holotype, ♀ A. Habitus, lateral aspect; B. Mesosoma, lateral aspect; C. Mesosoma, dorsal aspect; D. Fore wing; E. Hind wing; F. Ovipositor sheath; G. Head, anterior aspect; H. Head, dorsal aspect; I. Head, lateral aspect; J. First metasomal tergite, lateral aspect; K. First metasomal tergite, dorsal aspect; L. Fore femur, lateral aspect; M. Fore tibial spur and fore basitarsus; N. Hind femur, lateral aspect. Scale bars: 500 μm.

Eyes more protruding and temples more directly narrowed in dorsal view (Fig. 11H); vein m-cu of fore wing slightly curved (Fig. 11D); anterior tentorial pits close to eyes (Fig. 11G) second tergite black; mandible yellowish (except dark apex) (Fig. 11G); pterostigma pale yellowish (Fig. 11D); first tergite ~2.3× longer than its apical width; dorsope of first tergite small and space between dorsope much wider than dorsope and sculptured (Fig. 11K); hind tarsus mainly white; ovipositor sheath ~0.24× as long as fore wing.

##### Comparative diagnosis.

Very similar to *Z.shaanxiensis* but differs mainly by the more protruding eyes (less protruding in *Z.shaanxiensis*), curved vein m-cu of fore wing (straight in *Z.shaanxiensis*) and anterior tentorial pits close to eyes (distinctly removed from eyes in *Z.shaanxiensis*).

##### Description.

Holotype, ♀, length of fore wing 8.0 mm, of body 8.0 mm, and antenna 1.2× as long as fore wing.

***Head*.** Antennal segments 42, third segment nearly as long as fourth segment and third, fourth and penultimate segments 3.3×, 3.1×, and 1.8× longer than wide, respectively; length of maxillary palp 1.5× longer than height of head; frons smooth and behind antennal sockets impressed; POL: diameter of posterior ocellus: OOL = 12: 10: 5; vertex convex, punctulate and densely setose (Fig. 11H); clypeus rather convex in lateral view, widely smooth, punctate medially (Fig. 11I); face largely smooth, it widened ventrally, minimum width of face 1.1× height of face (Fig. 11G); length of eye 2.2× temple in dorsal view (Fig. 11H); length of malar space 0.3× basal width of mandible.

***Mesosoma*.** Length of mesosoma 1.6× its height; side of pronotum reticulate-rugulose ventrally and posteriorly, rugose anteriorly; epicnemial area reticulate-rugulose; precoxal sulcus densely strigate-rugose dorsally, narrowly reticulate-punctate antero-ventrally; dorsal of mesopleuron largely smooth (Fig. 11B); mesosternum almost smooth, finely punctulate; metapleuron smooth anteriorly, and widely scabrous posteriorly; mesoscutal lobes finely punctulate and shiny; notauli anteriorly finely and narrowly crenulate, mesoscutum medio-posteriorly widely crenulate-rugose and with a long carina; scutellar sulcus deep and rather narrow with one long obvious median carina; scutellum rather convex and finely punctulate; metanotum with small smooth knob medio-posteriorly, with one short media carina on knob, with two weakly converging long carinae medially; propodeum widely reticulate-rugose, subbasal transverse carina of propodeum mainly straight to curved posteriad, comparatively finely punctulate anteriorly; in lateral view propodeum gradually lowered posteriorly (Fig. 11B, C).

***Wings*.** Fore wing (Fig. 11D): r:3-SR:SR1 = 9:20:105; 2-SR:3-SR: r-m = 23:20:14; 1-CU1:2-CU1 = 3:60; cu-a vertical, postfurcal. Hind wing (Fig. 11E): r absent; M+CU:1-M = 62:12; 1r-m 3.3× 1-M; marginal cell strongly widened apically (Fig. 11E).

***Legs*.** Hind coxa densely punctate dorsally; length of fore femur 8.0× its width (Fig. 11L); length of fore tibial spur 0.3× fore basitarsus (Fig. 11M); lengths of hind femur and basitarsus 7.3× and 8.5× their widths, respectively (Fig. 11N).

***Metasoma*.** First tergite 2.3× longer than its apical width, its surface rugulose; dorsope comparatively small and narrow, area in front of dorsope depressed (Fig. 11K), laterope comparatively narrow (Fig. 11J); second tergite mainly bare, smooth; ovipositor comparatively robust basally; length of ovipositor sheath 0.24× length of fore wing, sheath with moderately erect and short setae (Fig. 11F).

***Colour*.** Mesosoma, metasoma, coxae and trochanters of middle and hind legs, hind femur, hind tibia (except dark brown apical 1/3) black; head, fore and middle legs mainly brownish yellow; hind tarsus white; antenna and veins C+SC+R, 1-M and cu-a of fore wing dark brown; palpi, pterostigma and other veins pale yellow; and ovipositor sheath (except pale whitish apex) brown.

***Variation*.** Vein 1r-m of hind wing 3.3–3.6× as long as vein 1-M; fore femur of ♀ 8.0–8.5× longer than wide; hind femur of ♀ 7.0–7.3× longer than wide; first metasomal tergite 2.3–2.5× its apical width.

##### Distribution.

China (Qinghai, Sichuan).

##### Biology.

Unknown.

##### Etymology.

Named after the slightly curved vein m-cu of the fore wing; *curvus* and *nervus* is Latin respectively for curved and vein.

#### ﻿ Zele deceptor

Taxon classificationAnimaliaHymenopteraBraconidae

(Wesmael, 1835)

5B65A8E2-D8E3-5797-8CEC-E97A18CED26F

Fig. 12

Perilitus deceptor Wesmael, 1835: 26.
Zele albiditarsus f. deceptor : van Achterberg 1979: 377–379 (lectotype designation).
Zele deceptor f. deceptor : van Achterberg 1984: 110–112 (with Z.deceptor re-instated as valid species); Chen et al. 1987: 94.

##### Type material examined.

***Lectotype*** of *Perilitusdeceptor*. Belgium • ♀; 1774; Coll Wesmael leg.; (KBIN Brussels).

##### Other material examined.

China – **Guizhou Prov.** • 1 ♀; Dashahe; 25 Aug. 2004; light trap; (ZJUH) No. 202401059. – **Heilongjiang Prov.** • 1 ♂; Harbin, Shangzhi, Mt. Maoer, Laoyeling; 5 Aug. 2023; Chun-hong Wang leg.; (ZJUH) No. 202401054. • 1 ♀; Yichun, Dajingshan, Liangshui Nature Reserve; 1 Aug. 2023; Jia-chen Zhu leg.; (ZJUH) No. 202401053. – **Liaoning Prov.** • 1 ♀; Shenyang, Laotuding; 15 Jul. 2011; Cheng-jin Yan leg.; (ZJUH) No. 202401052. – **Nei Mongol Zizhiqu** • 1 ♀; Mt. Helan, Luanchaigou; 26 Jul. 2010; Cheng-jin Yan leg.; (ZJUH) No. 202401051. – **Ningxia Huizu Zizhiqu** • 1 ♂; Mt. Liupan, Guamagou; 9 Jul. 2008; Jing-xian Liu leg.; (ZJUH) No. 202401081. – **Sichuan Prov.** • 1 ♀; Ganzizangzu Zizhizhou, Luding, Moxi; 18 Jun. 2005; Jing-xian Liu leg.; (ZJUH) No. 202401085. GenBank accession no. PV356301–PV356304, PV356309, PV356323, PV356324.

##### Diagnosis.

**Figure 12.**
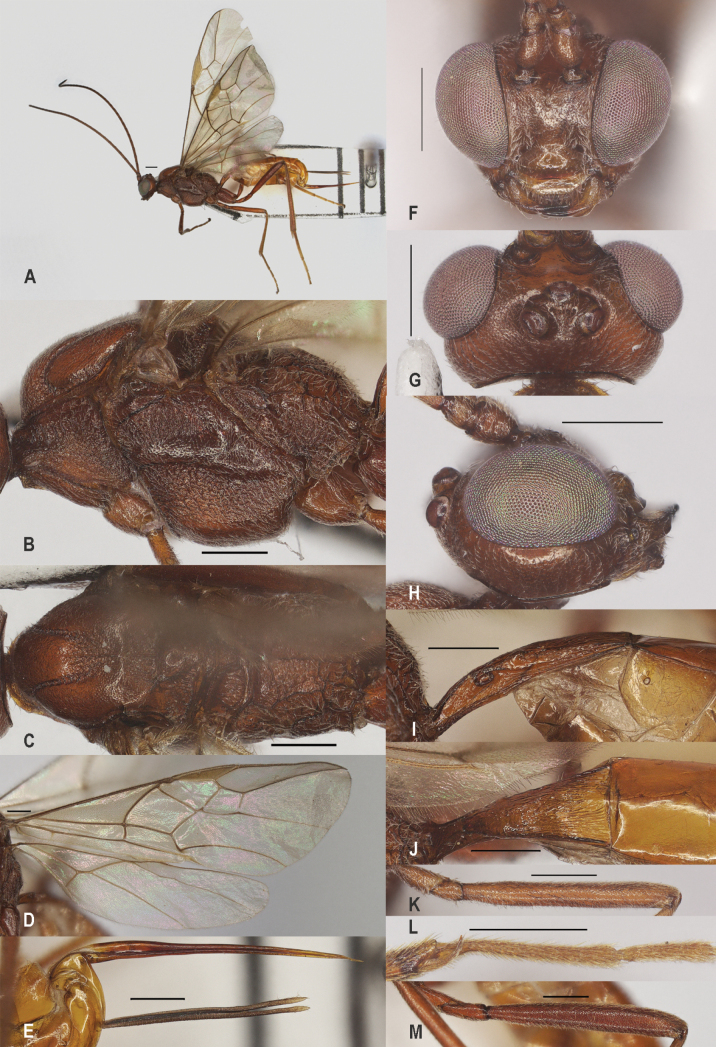
*Zeledeceptor* (Wesmael), China, Nei Mongol, ♀ A. Habitus, lateral aspect; B. Mesosoma, lateral aspect; C. Mesosoma, dorsal aspect; D. Wings; E. Ovipositor sheath; F. Head, anterior aspect; G. Head, dorsal aspect; H. Head, lateral aspect; I. First metasomal tergite, lateral aspect; J. First metasomal tergite, dorsal aspect; K. Fore femur, lateral aspect; L. Fore tibial spur and fore basitarsus; M. Hind femur, lateral aspect. Scale bars: 500 μm.

Subbasal carina of propodeum distinct diverging from anterior margin of propodeum medially with enclosed area narrow medially and superficially sculptured; vein 1-M of hind wing approximately as wide as vein M+CU; first tergite 2.1–2.5× longer than its apical width; dorsope of first tergite medium-sized, space between dorsope smooth and wider than dorsope (Fig. 12J); ovipositor sheath 0.26–0.32× as long as fore wing; first tergite largely finely sculptured (Fig. 12J); head yellowish brown (Fig. 12G) or pale brown; width of face 1.2–1.3× its height; first–third metasomal tergites yellowish brown (Fig. 12A); fore femur 7.0–8.6× longer than wide; hind femur of ♀ 6.2–7.6× its maximum width; setae of ovipositor adpressed or largely so; hind tibia brownish yellow; hind tarsus brownish yellow or yellowish brown, rarely pale yellowish.

##### Distribution.

Armenia, Albania, Austria, Belgium, Bulgaria, Canada, China (Anhui, Fujian, Guizhou, Hebei, Heilongjiang, Hubei, Hunan, Liaoning, Nei Mongol, Ningxia, Shaanxi, Sichuan, Taiwan, Xizang, Yunnan, Zhejiang), Croatia, former Czechoslovakia, Denmark, Finland, France, Georgia, Germany, Hungary,, Ireland, Italy, Japan, Kazakhstan, Latvia, Lithuania, Mexico, Netherlands, Norway, Poland, Romania, Russia, Slovakia, Sweden, Switzerland, United Kingdom, former Yugoslavia.

##### Biology.

Parasitoids of Geometridae, Saturniidae, Noctuidae, and Pyralidae.

#### ﻿ Zele densipunctatus

Taxon classificationAnimaliaHymenopteraBraconidae

Fang, van Achterberg & Chen sp. nov.

CEA76E02-4481-55A0-BBDB-50643D135237

https://zoobank.org/D62F65E2-FBB8-4BB9-89CF-BD58C154310F

Fig. 13

##### Type material.

***Holotype*.** China – **Guizhou Prov.** • ♀; Tongren, Jiangkou, Mt. Fanjing; 25 Aug. 2001; light trap; (ZJUH) No. 202401061. ***Paratypes*.** China – **Fujian Prov.** • 1 ♀; Mt. Wuyi, Chongan, Mt. Huanggang; 22 Jul. 1980; Nai-quan Lin leg.; (ZJUH) No. 20003955. • 1 ♂; same locality as for preceding; 28 Jun. 1984; Ju-chang Huang leg.; (ZJUH) No. 202315013. • 1 ♀; same locality as for preceding; 13 Jul. 1985; Dong-hong Huang leg.; No. 202315014. • 3 ♀♀, 1 ♂; same locality as for preceding; 13 Jul. 1985; Yu-chuan Tang leg.; (ZJUH) Nos. 20003963, 20003964, 20003966, 20003967. • 2 ♀♀; same locality as for preceding; 30 Jul. 1985; Nai-quan Lin leg.; (ZJUH) Nos. 202315015, 202315016. • 1 ♀; same locality as for preceding; 30 Jul. 1985; Ming-hui Liu leg.; (ZJUH) No. 202315017. • 1 ♀; same locality as for preceding; 30 Jul. 1985; Geng Zheng leg.; (ZJUH) No. 202315018. • 1 ♀; same locality as for preceding; 14 Jun. 1985; Xin-quan Chen leg.; (ZJUH) No. 202315019. • 3 ♀♀; same locality as for preceding; 30 Jul–1 Aug. 1985; Dong-hong Huang leg.; (ZJUH) Nos. 202315020–202315022. • 1 ♀; same locality as for preceding; • 1 Aug. 1985; Xuan-zhang Zhao leg.; (ZJUH) No. 202315023. • 2 ♂♂; same locality as for preceding; 14 Jun. 1994; Xue-xin Chen leg.; (ZJUH) Nos. 942615, 942617. • 1 ♂; same locality as for preceding; 17 Jun. 1994; Ping Cai leg.; (ZJUH) No. 943578. • 2 ♂♂; same locality as for preceding; 17 Jun. 1994; Zai-fu Xu leg.; (ZJUH) Nos. 942995, 942996. • 1 ♂; same locality as for preceding; 17 Jun. 1994; Shu-feng Ye leg.; (ZJUH) No. 943346. – **Guizhou Prov.** • 1 ♀; Mt. Fanjing, Jinding; alt. 1481 m; 30 Jul. 2001; Yun Ma leg.; (ZJUH) No. 200109310. • 1 ♀; Mt. Fanjing; 25 Aug. 2001; light trap; (ZJUH) No. No.202401061. • 1 ♀; Daozhen Gelaozu Miaozu Zizhixian, Dashahe National Nature Reserve; 25 Aug. 2004; light trap; (ZJUH) No. 202401062. – **Hunan Prov.** • 1 ♀; Zhangjiajie, Sangzhi, Mt. Tianping; Jun. 1984; Bao-wen Sun leg.; (ZJUH) No. 846398. – **Sichuan Prov.** • 1 ♀; Mt. Wu, Liziping; 4 Aug. 1993; Bao-wen Sun leg.; (ZJUH) No. 12569. GenBank accession no. PV356311, PV356312.

##### Diagnosis.

**Figure 13.**
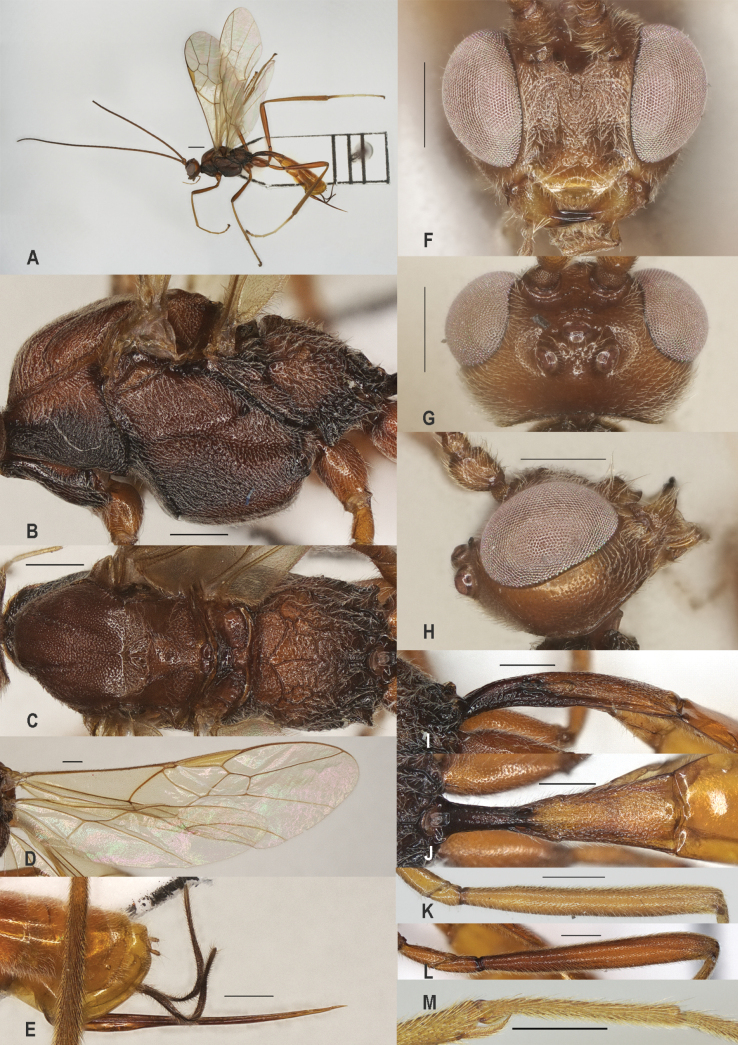
*Zeledensipunctatus* sp. nov., holotype, ♀ A. Habitus, lateral aspect; B. Mesosoma, lateral aspect; C. Msoasoma, dorsal aspect; D. Wings; E. Ovipositor sheath; F. Head, anterior aspect; G. Head, dorsal aspect; H. Head, lateral aspect; I. First metasomal tergite, lateral aspect; J. First metasomal tergite, dorsal aspect; K. Fore femur, lateral aspect; L. Hind femur, lateral aspect; M. Fore tibial spur and fore basitarsus. Scale bars: 1000 μm (A); 500 μm (B–M).

Entire middle lobe of mesoscutum densely punctate and rather dull, brown without posterior rugae or rugulae (Fig. 13C); hind femur 7.5–9.0× longer than wide (Fig. 13L); length of first tergite 2.6–2.8× its maximum width (Fig. 13I. J); face 1.3–1.4× wider than high (Fig. 13F); dorsope of first tergite large and space between dorsope approximately as wide as dorsope (Fig. 13J); hind tarsus mainly white; ovipositor sheath 0.25–0.33× as long as fore wing.

##### Comparative diagnosis.

Very similar to *Z.deceptor* (Wesmael) but differs mainly by the densely punctate and rather dull entire middle lobe of mesoscutum (smooth or nearly so in *Z.deceptor*), large dorsope and space between dorsope approximately as wide as dorsope (small in *Z.deceptor*) and slender first tergite, 2.6–2.8× its maximum width (robust in *Z.deceptor* and 2.1–2.5× its maximum width).

##### Description.

Holotype, ♀, length of fore wing 8.9 mm, of body 9.8 mm, and antenna 1.2× as long as fore wing.

***Head*.** Antennal segments 46, third segment 0.9× shorter than fourth segment and third, fourth and penultimate segments 3.0×, 4.2×, and 2.0× longer than wide, respectively; length of maxillary palp 1.6× longer than height of head; frons sculptured and behind antennal sockets impressed; POL: diameter of posterior ocellus: OOL = 12: 10: 6; vertex convex, punctulate and densely setose (Fig. 13G); clypeus rather convex in lateral view, punctate dorsally, smooth ventrally (Fig. 13H); face rugulose medially, punctate near antenna an it widened ventrally, minimum width of face 1.3× height of face (Fig. 13F); length of eye 2.0× temple in dorsal view (Fig. 13G); length of malar space 0.3× basal width of mandible.

***Mesosoma*.** Length of mesosoma 1.6× its height; side of pronotum striate-rugose ventrally, crenulate-rugose medially, crenulate postero-dorsally; epicnemial area anteriorly punctate-rugose, posteriorly rugose; precoxal sulcus densely reticulate-rugose dorsally, reticulate-punctate ventrally; dorsal of mesopleuron widely reticulate-punctate (Fig. 13B); mesosternum obviously punctate; metapleuron mainly rugose, postero-dorsally striate; mesoscutal lobes densely punctate and rather dull medially; notauli anteriorly widely crenulate, mesoscutum medio-posteriorly narrowly crenulate-rugose; mesoscutum medio- posteriorly with a long carina; scutellar sulcus deep and rather wide with one long obvious median carina; scutellum rather convex and punctulate; metanotum with small smooth knob medio-posteriorly, without median carina in front of it; propodeum widely reticulate-rugose, subbasal carina of propodeum mainly straight to curved posteriad, comparatively coarsely punctate anteriorly; propodeum without long straight median carina, in lateral view gradually lowered posteriorly, posterior part not separated from antero-dorsal part distinctly (Fig. 13B, C).

***Wings*.** Fore wing (Fig. 13D): r:3-SR:SR1 = 16:30:152; 2-SR:3-SR: r-m = 35:30:20; cu-a vertical, interstitial. Hind wing (Fig. 13D): r absent; M+CU:1-M = 85:12; 1r-m 3.3× 1-M.

***Legs*.** Hind coxa densely punctate dorsally; length of fore femur 9.5× its width (Fig. 13K); length of fore tibial spur 0.3× fore basitarsus (Fig. 13M); lengths of hind femur and basitarsus 9.0× and 12.6× their widths, respectively (Fig. 13L).

***Metasoma*.** First tergite 2.7× longer than its apical width, its surface rugulose; dorsope comparatively large, oval (Fig. 13J), laterope narrow (Fig. 13I); second tergite mainly bare, smooth; ovipositor comparatively robust basally; length of ovipositor sheath 0.23× as long as fore wing, sheath with rather erect and long setae (Fig. 13E).

***Colour*.** Basal 1/2 of antenna, head, mandible (except black apex), mesosoma (somewhat black), legs (except hind tarsus), metasoma (but base of first tergite before dorsope black) largely brownish yellow; apical 1/2 apical of antenna and ovipositor sheath (except pale brown apex) mainly dark brown; hind tarsus largely ivory, but its telotarsus dorsally and base of basitarsus brownish yellow; palpi, pterostigma pale yellow; wings subhyaline with some infuscation.

***Variation*.** Vein 1r-m of hind wing 3.3–4.0× as long as vein 1-M; fore femur of ♀ 8.2–9.5× longer than wide; hind femur of ♀ 7.5–9.0× longer than wide; first metasomal tergite 2.6–2.8× longer than its apical width. Colour of ♀ largely reddish; ♂ antenna, mesosoma (except reddish mesonotum), coxae and metasoma largely black; head mainly orange-yellow but frons and stemmaticum black; legs reddish (except largely ivory hind tarsus); pterostigma pale yellow; antennal segments of ♀ 46–50, of ♂ 49.

##### Distribution.

China (Fujian, Guizhou, Hunan, Sichuan).

##### Biology.

Unknown.

##### Etymology.

Named after the densely punctate middle lobe of mesoscutum; *densipunctatus* is Latin for densely punctate.

#### ﻿ Zele extensus

Taxon classificationAnimaliaHymenopteraBraconidae

Fang, van Achterberg & Chen sp. nov.

D893B275-0929-54EA-9D35-1AB59E172385

https://zoobank.org/7D0EED8D-26DE-46A1-9736-0938D8EBBAE6

Fig. 14

##### Type material.

***Holotype*.** China – **Sichuan Prov.** • ♀; Panzhihua, Yixian, Puwei, Pengjiayakou; 5 Jun. 2024; Xiao-qing Zhang, Ying Wang, Qing-zhen Meng leg.; tree canopy; (ZJUH) No. 202402467. GenBank accession no. PV356305.

##### Diagnosis.

**Figure 14.**
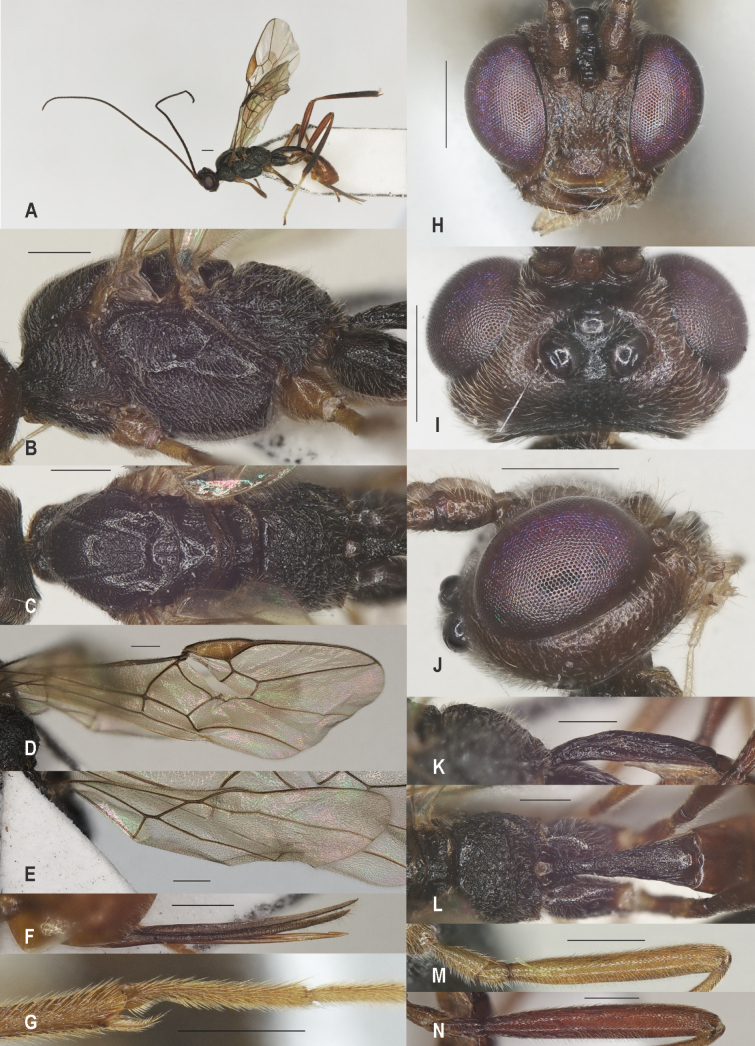
*Zeleextensus* sp. nov., holotype, ♀ A. Habitus, lateral aspect; B. Mesosoma, lateral aspect; C. Mesosoma, dorsal aspect; D. Fore wing; E. Hind wing; F. Ovipositor sheath; G. Fore tibial spur and fore basitarsus; H. Head, anterior aspect; I. Head, dorsal aspect; J. Head, lateral aspect; K. First metasomal tergite, lateral aspect; L. First metasomal tergite, dorsal aspect; M. Fore femur, lateral aspect; N. Hind femur, lateral aspect. Scale bars: 500 μm.

Vein 2-SC+R of hind wing distinctly longer than vein 1-M (Fig. 14E); metanotum with three distinct parallel carinae and no knob medio-posteriorly (Fig. 14C); tip of ovipositor sheath dark brown (Fig. 14F); ovipositor sheath ~0.26× as long as fore wing; hind tarsus mainly whitish yellow; dorsope of first tergite small, area between dorsope much wider than dorsope (Fig. 14L); [basal 1/3 of first tergite comparatively wide in lateral view (Fig. 14K); eyes distinctly protruding in dorsal view; fore wing and first subdiscal cell comparatively narrow; pterostigma infuscated (Fig. 14D)].

##### Comparative diagnosis.

Similar to *Z.rugulosus* but differs mainly by longer vein 2-SC+R of hind wing (compared with vein 1-M of hind wing) (shorter in *Z.rugulosus*), narrow subdiscal cell (wide in *Z.rugulosus*) and dark brown tip of ovipositor sheath (pale yellowish in *Z.rugulosus*).

##### Description.

Holotype, ♀, length of fore wing 6.7 mm, of body 7.1 mm, and antenna 1.3× as long as fore wing.

***Head*.** Antennal segments 44, third segment nearly as long as fourth segment and third, fourth and penultimate segments 3.8×, 3.6×, and 1.6× longer than wide, respectively; length of maxillary palp 1.2× longer than height of head; frons partly rugose and behind antennal sockets impressed; POL: diameter of posterior ocellus: OOL = 15: 12: 8; vertex convex, punctulate, matt and densely setose (Fig. 14I); clypeus slightly convex in lateral view, widely punctate dorsally, smooth ventrally (Fig. 14J); face widely rugulose, punctulate near antennal sockets and eyes, narrowed ventrally, minimum width of face 1.1× height of face (Fig. 14H); length of eye 2.2× temple in dorsal view (Fig. 14I); length of malar space 0.2× basal width of mandible.

***Mesosoma*.** Length of mesosoma 1.7× its height; side of pronotum striate-rugose ventrally, reticulate-rugose medially, punctate postero-dorsally; epicnemial area largely reticulate-rugose; precoxal sulcus widely punctate-rugose ventrally; dorsal of mesopleuron largely smooth, finely punctate, anteriorly narrowly sculptured (Fig. 14B); mesosternum coarsely punctate; metapleuron broadly striate anteriorly, rugose posteriorly smooth; mesoscutal lobes widely punctulate and comparatively shiny; notauli anteriorly distinctly and narrowly crenulate, mesoscutum medio-posteriorly narrowly crenulate-rugose and with a medium-sized carina; scutellar sulcus deep and rather wide with one long obvious median carina; scutellum rather convex and finely punctulate; metanotum with three similar subparallel carinae medially; propodeum densely reticulate-rugose, subbasal carina of propodeum absent, comparatively smooth anteriorly; propodeum without long straight median carina; propodeum gradually lowered posteriorly in lateral view (Fig. 14B, C).

***Wings*.** Fore wing (Fig. 14D): r:3-SR:SR1 = 11:27:131; 2-SR:3-SR: r-m = 27:27:16; cu-a vertical, interstitial. Hind wing (Fig. 14E): r absent; M+CU:1-M = 77:12; 1r-m 3.0× 1-M.

***Legs*.** Hind coxa largely smooth dorsally; length of fore femur 7.0× its width (Fig. 14M); length of fore tibial spur 0.3× fore basitarsus (Fig. 14G); lengths of hind femur and basitarsus 7.2× and 9.0× their widths, respectively (Fig. 14N).

***Metasoma*.** First tergite 2.7× longer than its apical width, its surface largely irregularly rugulose; dorsope narrow elliptical, elongate, and medium-sized, area in front of dorsope depressed (Fig. 14L), laterope comparatively small but elongate (Fig. 14K); second tergite mainly bare, smooth and shiny; ovipositor comparatively robust basally; length of ovipositor sheath 0.26× as long as fore wing, sheath with moderately erect and short setae (Fig. 14F).

***Colour*.** Mesosoma, hind coxa, hind tibia (except apical fourth brown), first metasomal tergite largely black,; antenna, head, mandible, veins C+SC+R, 1-M, and cu-a of fore wing, ovipositor sheath (except pale brown apex) mainly dark brown; palpi, fore and middle legs, metasoma (except first metasomal tergite) yellowish brown; hind femur reddish brown; hind tarsus largely whitish yellow, but its telotarsi dorsally and base of basitarsus yellowish brown; pterostigma brown.

##### Distribution.

China (Sichuan).

##### Biology.

Unknown.

##### Etymology.

Named after the comparatively stretched dorsope of the first metasomal tergite; *extensus* is Latin for stretched.

#### ﻿ Zele fulgidus

Taxon classificationAnimaliaHymenopteraBraconidae

Fang, van Achterberg & Chen sp. nov.

1C568AC3-FBA7-5906-827F-966AF18B736E

https://zoobank.org/7EE03045-598A-4240-90B1-562B48E8B3ED

Fig. 15

##### Type material.

***Holotype*.** China – **Xizang Zizhiqu** • ♀; Bomi; 9 Jul. 2013; Zhen Liu leg.; (ZJUH) No. 201405825.

##### Diagnosis.

**Figure 15.**
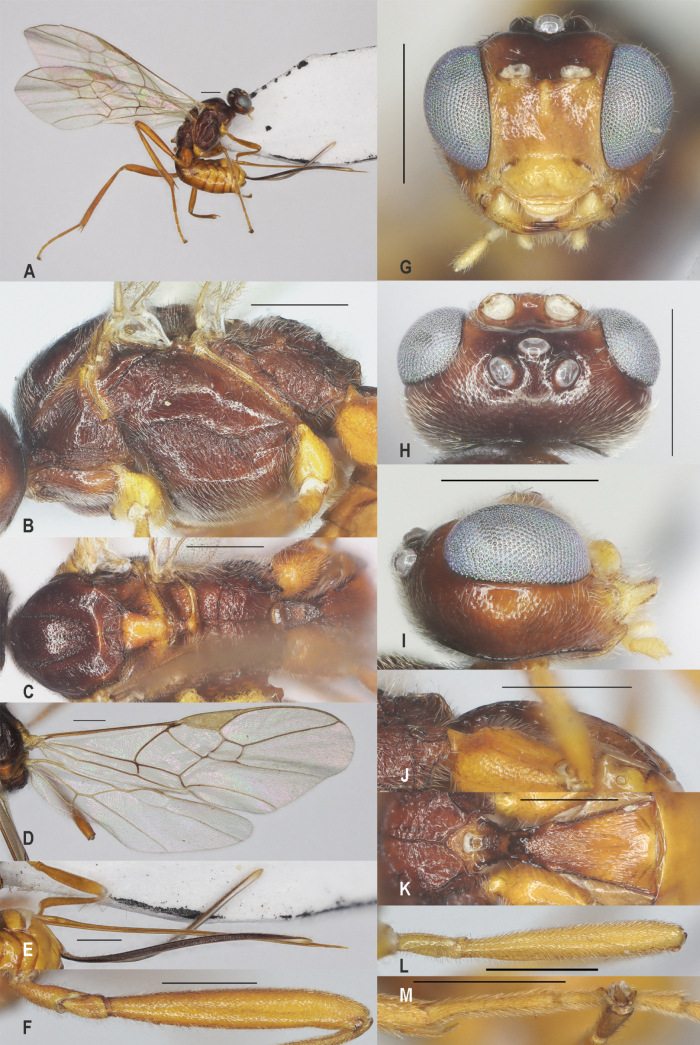
*Zelefulgidus* sp. nov., holotype, ♀ A. Habitus, lateral aspect; B. Mesosoma, lateral aspect; C. Mesosoma, dorsal aspect; D. Wings; E. Ovipositor sheath; F. Hind femur, lateral aspect; G. Head, anterior aspect; H. Head, dorsal aspect; I. Head, lateral aspect; J. First metasomal tergite, lateral aspect; K. First metasomal tergite, dorsal aspect; L. Fore femur, lateral aspect; M. Fore tibial spur and fore basitarsus Scale bars: 500 μm.

Length of first metasomal tergite 1.7× its apical width; first tergite robust, petiolate part comparatively short and dorsope close to base of tergite (Fig. 15J, K); precoxal sulcus comparatively narrowly sculptured anteriorly and mesopleuron shiny (Fig. 15B); in dorsal view eyes of ♀ 1.2× as long as temple (Fig. 15H); malar space 0.6× as long as basal width of mandible; scutellum yellowish, strongly contrasting with dark brown mesosoma (Fig. 15C); pterostigma of ♀ pale yellowish (Fig. 15D); dorsope of first tergite medium-sized and area between dorsope wider than dorsope (Fig. 15K); hind tarsus yellowish; ovipositor sheath ~0.58× as long as fore wing.

##### Comparative diagnosis.

Similar to *Z.annulicrus* but differs mainly by longer ovipositor sheath, ~0.58× as long as fore wing (shorter in *Z.annulicrus*), longer malar space (shorter in *Z.annulicrus*) and straight hind femur (swollen in *Z.annulicrus*).

##### Description.

Holotype, ♀, length of fore wing 4.8 mm, of body 4.7 mm.

***Head*.** Antenna missing; maxillary palp broken; frons smooth and behind antennal sockets impressed; POL: diameter of posterior ocellus: OOL = 5: 3: 6; vertex nearly smooth and setose (Fig. 15H); clypeus strongly convex in lateral view, weakly punctate (Fig. 15I); face largely smooth and shiny, slightly punctulate near antennal sockets, minimum width of face 1.5× height of face (Fig. 15G); length of eye 1.2× temple in dorsal view (Fig. 15H); length of malar space 0.6× basal width of mandible.

***Mesosoma*.** Length of mesosoma 1.8× its height; side of pronotum crenulate antero-ventrally, widely smooth and shiny postero-dorsally; epicnemial area slightly sculptured; precoxal sulcus comparatively narrowly crenulate, remainder of mesopleuron distinctly smooth and shiny (Fig. 15B); mesosternum smooth, finely punctulate; metapleuron almost smooth, ventrally with some crenulae; mesoscutal lobes punctulate and shiny; notauli comparatively narrowly crenulate, mesoscutum medio-posteriorly widely crenulate-rugose and with a medium-sized carina. scutellar sulcus shallow and wide, with one long median carina; scutellum slightly convex, smooth; metanotum with medium-sized posterior knob and with two medium-sized carinae; propodeum mainly smooth dorsally, subbasal carina complete, angulate; medio-longitudinal carina complete, and mostly well developed; propodeum gradually lowered posteriorly except dorsal part in front of subbasal carina; posterior part not distinctly separated from antero-dorsal part distinctly in lateral view (Fig. 15B, C).

***Wings*.** Fore wing (Fig. 15D): r:3-SR:SR1 = 8:18:98; 2-SR:3-SR: r-m = 18:18:14; 1-CU1:2-CU1 = 3:50; cu-a nearly vertical, postfurcal. Hind wing (Fig. 15D): r absent; M+CU:1-M = 58:15; 1r-m 2.0× 1-M. marginal cell slightly widened (Fig. 15D).

***Legs*.** Hind coxa largely smooth dorsally; length of fore femur 7.1× its width (Fig. 15L); length of fore tibial spur 0.4× fore basitarsus (Fig. 15M); lengths of hind femur and basitarsus 6.5× and 9.7× their widths, respectively (Fig. 15F).

***Metasoma*.** First tergite 1.7× longer than its apical width, it robust, petiolate part comparatively short and dorsope close to base of tergite; its surface indistinctly strigulate but shiny; dorsope elliptical and medium-sized, area behind depressed (Fig. 15K), laterope sublateral (Fig. 15J); second tergite mainly bare, smooth and shiny; ovipositor comparatively slender basally; length of ovipositor sheath 0.58× as long as fore wing, sheath with less erect and short setae (Fig. 15E).

***Colour*.** Mesosoma (except yellowish scutellum), dorsal view of head, ovipositor sheath (but apex of ovipositor sheath yellowish) dark brown; face, legs, metasoma (first metasomal tergite between dorsope darked) yellowish; palpi, veins and pterostigma pale yellowish.

##### Distribution.

China (Xizang).

##### Biology.

Unknown.

##### Etymology.

Named after the shining mesopleuron; *fulgidus* is Latin for shiny.

#### ﻿ Zele fuscatus

Taxon classificationAnimaliaHymenopteraBraconidae

Fang, van Achterberg & Chen sp. nov.

77FF7752-5E7D-5B47-A6BD-73AEE6C65A61

https://zoobank.org/5DA91A10-25C1-41D2-B77D-38D105C4C751

Fig. 16

##### Type material.

***Holotype*.** China – **Shaanxi Prov.** • ♀; Xunyangba, Ningshan; 33.55°N, 108.55°E; alt. 1481 m; 20 May–23 Jun. 2016; Jiang-li Tan, Qing-qing Tan leg.; Black Malaise trap; (ZJUH) No. 202315004. ***Paratype*.** China – **Shaanxi Prov.** • 1 ♀; same data as for holotype; (NWU) No. 202315003.

##### Diagnosis.

**Figure 16.**
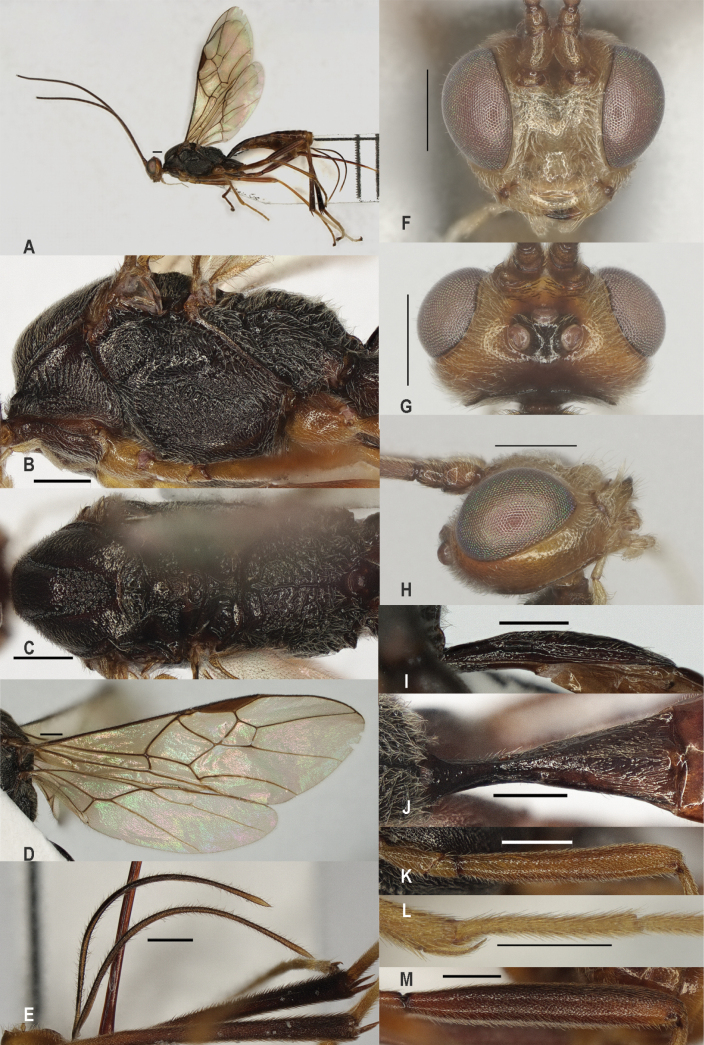
*Zelefuscatus* sp. nov., holotype, ♀ A. Habitus, lateral aspect; B. Mesosoma, lateral aspect; C. Mesosoma, dorsal aspect; D. Wings; E. Ovipositor sheath; F. Head, anterior aspect; G. Head, dorsal aspect; H. Head, lateral aspect; I. First metasomal tergite, lateral aspect; J. First metasomal tergite, dorsal aspect; K. Fore femur, lateral aspect; L. Fore tibial spur and fore basitarsus; M. Hind femur, lateral aspect. Scale bars: 500 μm.

Malar space 0.4× as long as basal width of mandible; pterostigma of ♀ dark brown (Fig. 16D); ventral 1/2 of temple yellowish, strongly contrasting with blackish mesosoma (Fig. 16G, C); face 1.4× wider than high (Fig. 16F); first tergite ~2.4× longer than its apical width; dorsope of first tergite small and area between dorsope much wider than dorsope (Fig. 16J); hind tarsus mainly white; ovipositor sheath ~0.45× as long as fore wing.

##### Comparative diagnosis.

Similar to *Z.petiolatus* but differs mainly by longer malar space (shorter in *Z.petiolatus*), wide face (narrow in *Z.petiolatus*) and dark brown pterostigma (pale brown in *Z.petiolatus*).

##### Description.

Holotype, ♀, length of fore wing 7.2 mm, of body 8.4 mm.

***Head*.** Antenna incomplete, third segment nearly as long as fourth segment and third and fourth segments 3.0× and 3.1× longer than wide, respectively; length of maxillary palp 1.4× longer than height of head; frons partly rugulose and behind antennal sockets impressed; POL: diameter of posterior ocellus: OOL = 10: 8: 11; vertex finely punctulate and densely setose; clypeus strongly convex in lateral view, weakly punctate (Fig. 16H); face almost smooth, widened ventrally, minimum width of face 1.4× height of face (Fig. 16F); length of eye 2.0× temple in dorsal view (Fig. 16G); length of malar space 0.4× basal width of mandible.

***Mesosoma*.** Length of mesosoma 1.7× its height; side of pronotum largely reticulate-crenulate and matt, with some striae ventrally; epicnemial area mainly reticulate-rugose; precoxal sulcus comparatively carinate dorsally, largely reticulate-rugose; remainder of mesopleuron smooth with few sculptured (Fig. 16B); mesosternum coarsely punctate; metapleuron narrowly smooth anteriorly, largely rugose-reticulate; mesoscutal lobes punctate and shiny; notauli rather widely crenulate, mesoscutum medio-posteriorly widely crenulate-rugose. scutellar sulcus deep and wide, with a long median carina; scutellum slightly convex, smooth; metanotum with small posterior knob and with three rather long carinae; propodeum mainly rugulose, subbasal carina of propodeum mainly straight to curved posteriad; medio-longitudinal carina complete, and mostly well developed; in lateral view propodeum gradually lowered posteriorly except for dorsal part in front of subbasal carina and this dorsal part comparatively large (Fig. 16B, C).

***Wings*.** Fore wing (Fig. 16D): r:3-SR:SR1 = 7:15:91; 2-SR:3-SR: r-m = 27:22:11; 1-CU1:2-CU1 = 5:70; cu-a nearly vertical, postfurcal. Hind wing: r absent; M+CU:1-M = 82:20; 1r-m 2.0× 1-M.

***Legs*.** Hind coxa widely punctate dorsally; length of fore femur 7.4× its width (Fig. 16K); length of fore tibial spur 0.3× fore basitarsus (Fig. 16L); lengths of hind femur and basitarsus 7.0× and 12.1× their widths, respectively (Fig. 16M).

***Metasoma*.** First tergite 2.4× longer than its apical width, it narrow medially, its surface finely strigate basally and shiny; dorsope elliptical and comparatively small, area behind dorsope depressed (Fig. 16J), laterope small and sublateral (Fig. 16I); second tergite mainly bare, smooth and shiny; ovipositor comparatively robust basally; length of ovipositor sheath 0.45× as long as fore wing, sheath with long semi-erect setae (Fig. 16E).

***Colour*.** Mesosoma, hind coxa, hind tibia (except apical 1/6) and part of hind femur and apical 1/2 of first metasomal tergite, ovipositor sheath (but apex pale brown) mainly black; head, face, legs except hind leg yellowish; hind tarsus largely white, but its telotarsus dorsally and base of basitarsus brownish yellow; pterostigma dark brown; wings subhyaline with slight infuscation.

***Variation*.** Vein 1r-m of hind wing 2.0–2.3× as long as vein 1-M; fore femur of ♀ 7.4–7.6× longer than wide; hind femur of ♀ 6.8–7.0× longer than wide;

##### Distribution.

China (Shaanxi).

##### Biology.

Unknown.

##### Etymology.

Named after the dark mesosoma, strongly contrasting with the ventral half of the yellowish temple; *fuscatus* is Latin for darkened.

#### ﻿ Zele impolitus

Taxon classificationAnimaliaHymenopteraBraconidae

Fang, van Achterberg & Chen sp. nov.

15CE9F54-3DFF-53F9-B237-8C7F3FB69166

https://zoobank.org/3112BC61-60A8-45FA-92AD-317B292E4FA0

Fig. 17

##### Type material.

***Holotype*.** China – **Sichuan Prov.** • ♀; Ganzizangzu Zizhizhou, Luding, Moxi; 19 Jun. 2005; light trap; (ZJUH) No. 202401057. GenBank accession no. PV356307.

##### Diagnosis.

**Figure 17.**
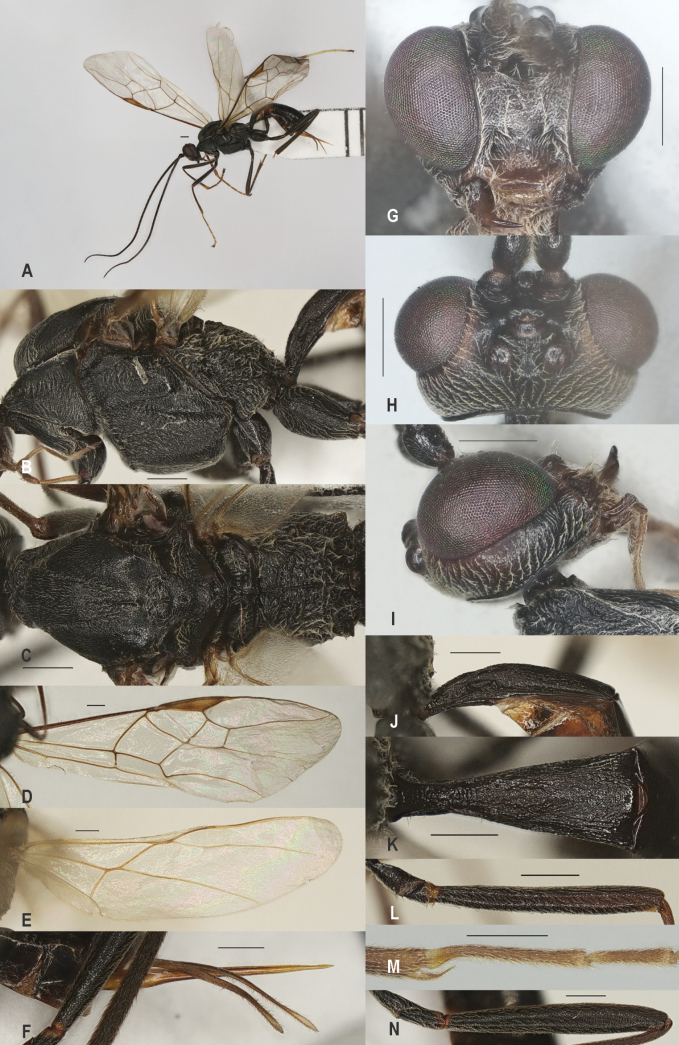
*Zeleimpolitus* sp. nov., holotype, ♀ A. Habitus, lateral aspect; B. Mesosoma, lateral aspect; C. Mesosoma, dorsal aspect; D. Fore wing; E. Hind wing; F. Ovipositor sheath; G. Head, anterior aspect; H. Head, dorsal aspect; I. Head, lateral aspect; J. First metasomal tergite, lateral aspect; K. First metasomal tergite, dorsal aspect; L. Fore femur, lateral aspect; M. Fore tibial spur and fore basitarsus; N. Hind femur, lateral aspect. Scale bars: 500 μm.

Pterostigma partly infuscated (Fig. 17D); lateral lobes of mesoscutum comparatively matt; metanotum with three medium-sized carinae dorsally and posterior knob keeled (Fig. 17C); anteriorly propodeum superficially sculptured between carinae (Fig. 17C); first tergite ~2.3× longer than its apical width; dorsope of first tergite small and area between dorsope much wider than dorsope (Fig. 17K); hind tarsus mainly whitish yellow; ovipositor sheath ~0.24× as long as fore wing; mandible dark brown or brown; laterope wide elliptical (Fig. 17K).

##### Comparative diagnosis.

Very similar to *Z.carinatus* but differs mainly by the pale yellow and partly infuscated pterostigma (dark brown in *Z.carinatus*), the weakly curved subbasal carina of propodeum (distinctly oblique in *Z.carinatus*) and the dark brown mandible (yellow except dark apex in *Z.carinatus*).

##### Description.

Holotype, ♀, length of fore wing 9.5 mm, of body 9.0 mm, and antenna 1.1× as long as fore wing.

***Head*.** Antennal segments 46, third segment 1.1× longer than fourth segment and third, fourth and penultimate segments 4.0×, 3.5×, and 2.0× longer than wide, respectively; length of maxillary palp 1.7× longer than height of head; frons smooth and behind antennal sockets impressed; POL: diameter of posterior ocellus: OOL = 10: 7: 5; vertex convex, punctulate and densely setose (Fig. 17H); clypeus rather convex in lateral view, widely smooth, only punctate medially (Fig. 17I); face largely punctulate and matt, it narrowed ventrally, minimum width of face 1.2× height of face (Fig. 17G); length of eye 2.3× temple in dorsal view (Fig. 17H); length of malar space 0.2× basal width of mandible.

***Mesosoma*.** Length of mesosoma 1.5× its height; side of pronotum striate-rugose ventrally, rugulose-crenulate medially, smooth postero-dorsally; epicnemial area rugulose anteriorly, striate posteriorly; precoxal sulcus narrowly crenulate-rugose dorsally, coarsely reticulate-rugose ventrally; dorsal of mesopleuron densely punctate anteriorly, reticulate-rugose medially, smooth posteriorly (Fig. 17B); mesosternum widely punctulate and comparatively matt; metapleuron mainly smooth and shiny, postero-ventrally rugose, postero-dorsally striate and smooth medially; mesoscutal lobes finely punctulate and comparatively matt; notauli anteriorly finely and narrowly crenulate; mesoscutum medio-posteriorly narrowly crenulate-rugose with a medium-sized carina; scutellar sulcus deep and rather wide with one long median carina; scutellum rather convex and widely punctulate; metanotum with small smooth knob medio-posteriorly, with three medium-sized carina in front of knob; propodeum widely reticulate-rugose, subbasal carina of propodeum irregular submedially, anterior area comparatively inconspicuously rugulose; propodeum with long straight median carina, and comparatively flat in lateral view, gradually lowered posteriorly and comparatively long (Fig. 17C).

***Wings*.** Fore wing (Fig. 17D): r:3-SR:SR1 = 8:20:101; 2-SR:3-SR: r-m = 26:24:18; 1-CU1:2-CU1 = 4:75; cu-a vertical, postfurcal. Hind wing (Fig. 17E): r absent; M+CU:1-M = 70:12; 1r-m 3.3× 1-M.

***Legs*.** Hind coxa densely punctate dorsally; length of fore femur 8.3× its width (Fig. 17L); length of fore tibial spur 0.3× fore basitarsus (Fig. 17M); lengths of hind femur and basitarsus 7.4× and 10.0× their widths, respectively (Fig. 17N).

***Metasoma*.** First tergite 2.3× longer than its apical width, its surface rugulose anteriorly, irregularly and distinctly rugose behind spiracles; dorsope narrow elliptical and comparatively small, area behind dorsope depressed (Fig. 17K), laterope comparatively small but deep (Fig. 17J); second tergite mainly bare, smooth; ovipositor comparatively robust basally; length of ovipositor sheath 0.24× as long as fore wing, sheath with moderately erect and short setae (Fig. 17F).

***Colour*.** Antenna, mesosoma, coxae, trochanters, femora of all legs, hind tibia and metasoma largely black; head, mandible, palpi, fore and middle tibiae, and tarsus, veins C+SC+R, 1-M and cu-a of fore wing, ovipositor sheath (except pale brown apex) mainly dark brown; hind tarsus largely whitish yellow, but its telotarsus dorsally and base of basitarsus dark brown; pterostigma pale yellowish but partly infuscated.

##### Distribution.

China (Sichuan).

##### Biology.

Unknown.

##### Etymology.

Named after the comparatively matt lateral lobes of the mesoscutum; *impolitus* is Latin for unpolished.

#### ﻿ Zele inclinator

Taxon classificationAnimaliaHymenopteraBraconidae

Fang, van Achterberg & Chen sp. nov.

7EF41E81-AF4C-5996-A6E0-4986977C6E1D

https://zoobank.org/11ABB7F6-BB24-4C53-B7CC-8144F8D1D764

Fig. 18

##### Type material.

**Holotype.** China – **Ningxia Huizu Zizhiqu** • ♀; Mt. Liupan, Heshangpu Forest Farm; 25 Jun. 2008; Jing-xian Liu leg.; (ZJUH) No. 202401065. ***Paratype*.** China – **Ningxia Huizu Zizhiqu** • 1 ♀; Liupan, Fengtai Forest Farm; 27 Jun. 2008; Jing-xian Liu leg.; (ZJUH) No. 202401064. GenBank accession no. PV356314, PV356315.

##### Diagnosis.

**Figure 18.**
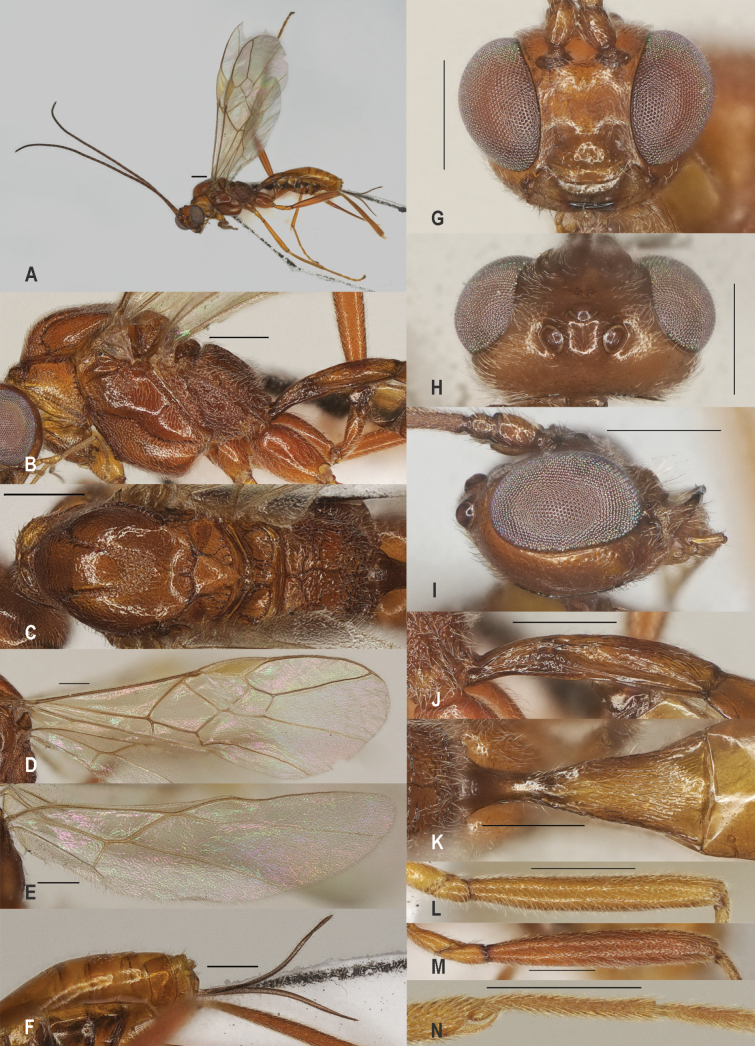
*Zeleinclinator* sp. nov., holotype, ♀ A. Habitus, lateral aspect; B. Mesosoma, lateral aspect; C. Mesosoma, dorsal aspect; D. Fore wing; E. Hind wing; F. Ovipositor sheath; G. Head, anterior aspect; H. Head, dorsal aspect; I. Head, lateral aspect; J. First metasomal tergite, lateral aspect; K. First metasomal tergite, dorsal aspect; L. Fore femur, lateral aspect; M. Hind femur, lateral aspect; N. Fore tibial spur and fore basitarsus. Scale bars: 500 μm.

Marginal cell of hind wing strongly widened apically (Fig. 18E); densely sculptured area of precoxal sulcus medium-sized medially (Fig. 18B); malar space ~0.2× as long as basal width of mandible; metanotum with enlarged smooth knob medio-posteriorly and median carina in front of it short (Fig. 18C); first tergite 2.1–2.2× longer than its apical width; dorsope of first tergite large and area between dorsope approximately as wide as dorsope and nearly smooth (Fig. 18K); hind tarsus yellowish; ovipositor sheath ~0.26× as long as fore wing; vein cu-a of fore wing slightly oblique (Fig. 18D).

##### Comparative diagnosis.

Similar to *Z.caligatus* but differs mainly by the strongly widened marginal cell of hind wing (slightly widened in *Z.caligatus*), the short malar space (long in *Z.caligatus*) and the enlarged smooth knob of metanotum (small in *Z.caligatus*).

##### Description.

Holotype, ♀, length of fore wing 6.1 mm, of body 6.3 mm, and antenna nearly as long as fore wing.

***Head*.** Antennal segments 38, third segment 0.9× shorter than fourth segment and third, fourth and penultimate segments 3.4×, 3.7×, and 1.8× longer than wide, respectively; length of maxillary palp 1.3× longer than height of head; frons smooth and behind antennal sockets impressed; POL: diameter of posterior ocellus: OOL = 9: 5: 3; vertex finely punctulate and densely setose (Fig. 18H); clypeus convex in lateral view, largely smooth (Fig. 18I); face widely smooth but punctulate near antennal sockets, it slightly narrowed ventrally (Fig. 18G), minimum width of face 1.3× height of face; length of eye 2.5× temple in dorsal view (Fig. 18H); length of malar space 0.2× basal width of mandible.

***Mesosoma*.** Length of mesosoma 1.6× its height; side of pronotum largely smooth, with some striae medially and ventrally; epicnemial area widely smooth, narrowly rugulose postero-dorsally; precoxal sulcus finely rugulose anteriorly, and narrowly short striate posteriorly; dorsal of mesopleuron widely smooth and shiny (Fig. 18B); mesosternum smooth and shiny; metapleuron smooth medially but striate-rugose anteriorly and posteriorly; mesoscutal lobes finely punctulate and shiny; notauli indistinctly crenulate; scutellar sulcus shallow, with a long median carina and four short indistinctly ones; scutellum slightly convex, weakly punctate; metanotum with enlarged smooth knob medio-posteriorly and short median carina in front of it; propodeum remotely reticulate, without subbasal transverse carina and medio-longitudinal carina; propodeum gradually lowered posteriorly, posterior part not distinctly separated from antero-dorsal part distinctly (Fig. 18B, C).

***Wings*.** Fore wing (Fig. 18D): r:3-SR:SR1 = 5:11:50; 2-SR:3-SR: r-m = 10:11:8; 1-CU1:2-CU1 = 5:52; cu-a oblique, postfurcal. Hind wing (Fig. 18E): r absent; M+CU:1-M = 85:15; 1r-m 3.0× 1-M.

***Legs*.** Hind coxa largely smooth and shiny dorsally; length of fore femur 7.4× its width (Fig. 18L); length of fore tibial spur 0.3× fore basitarsus (Fig. 18N); lengths of hind femur and basitarsus 7.0× and 13.3× their widths, respectively (Fig. 18M).

***Metasoma*.** First tergite 2.2× longer than its apical width, it narrow the apical 1/3, its surface indistinctly striate and shiny; dorsope elliptical, comparatively large, area behind depressed (Fig. 18K), laterope comparatively large and deep (Fig. 18J); second tergite mainly bare, smooth and glossy; ovipositor comparatively slender basally; length of ovipositor sheath 0.26× length of fore wing, sheath with short semi-erect setae (Fig. 18F).

***Colour*.** Mainly yellowish; apical 1/2 of antenna and ovipositor sheath brown; veins and pterostigma pale yellow.

***Variation*.** Vein 1r-m of hind wing 2.0–3.0× as long as vein 1-M; fore femur of ♀ 7.4–8.0× longer than wide; hind femur of ♀ 6.0–7.0× longer than wide; first metasomal tergite 2.2–2.3× its apical width. Antennal segments of ♂ unknown.

##### Distribution.

China (Ningxia).

##### Biology.

Unknown.

##### Etymology.

Named after the oblique vein cu-a of the fore wing; *clino* is Latin for slope.

#### ﻿ Zele irregularis

Taxon classificationAnimaliaHymenopteraBraconidae

Fang, van Achterberg & Chen sp. nov.

06C7812E-CC60-5888-8ADF-5F2A4C89624F

https://zoobank.org/93C4E9E2-5D2E-492E-915A-DD1A81D53862

Fig. 19

##### Type material.

***Holotype*.** China – **Jilin Prov.** • ♀; Yanbian, Huangsongpu Forest Farm; 4 Aug. 2004; light trap; (ZJUH) No. 202401070. ***Paratypes*.** China – **Gansu Prov.** • 1 ♀; Mt. Qingsong; 9 Aug. 1991; Ling Li leg.; (ZJUH) No. 974297. – **Henan Prov.** • 1 ♀; Ludai, Qihe; 29 Aug. 1996; Ping Cai leg.; (ZJUH) No. 973261. – **Jilin Prov.** • 2 ♀♀; Yanbian, Huangsongpu Forest Farm; 4 Aug. 2004; Yu-zhou Du, Zhi-jie Wang leg.; (ZJUH) Nos. 202401068, 202401069. • 1 ♀; Wangqing; 21 May 1980; En-yu Jin leg.; (ZJUH) No. 948232.– **Shaanxi Prov.** • 1 ♀; Niubeiliang; 33.76°N, 108.77°E; alt. 1135 m; 11 Aug. 2013; Bing-bing Tu leg.; light trap; (ZJUH) No. 201300221. • 2 ♀♀; Qingling, Chezhan; 18 Aug. 1965; Yao Zhou leg.; (ZJUH) Nos. 200011735, 200011736. • 1 ♀; Zhouzhi, Banfangzi; 7 Sep. 1994; Xue-yuan Lin leg.; (ZJUH) No. 200011632. GenBank accession no. PV356318, PV356319.

##### Diagnosis.

**Figure 19.**
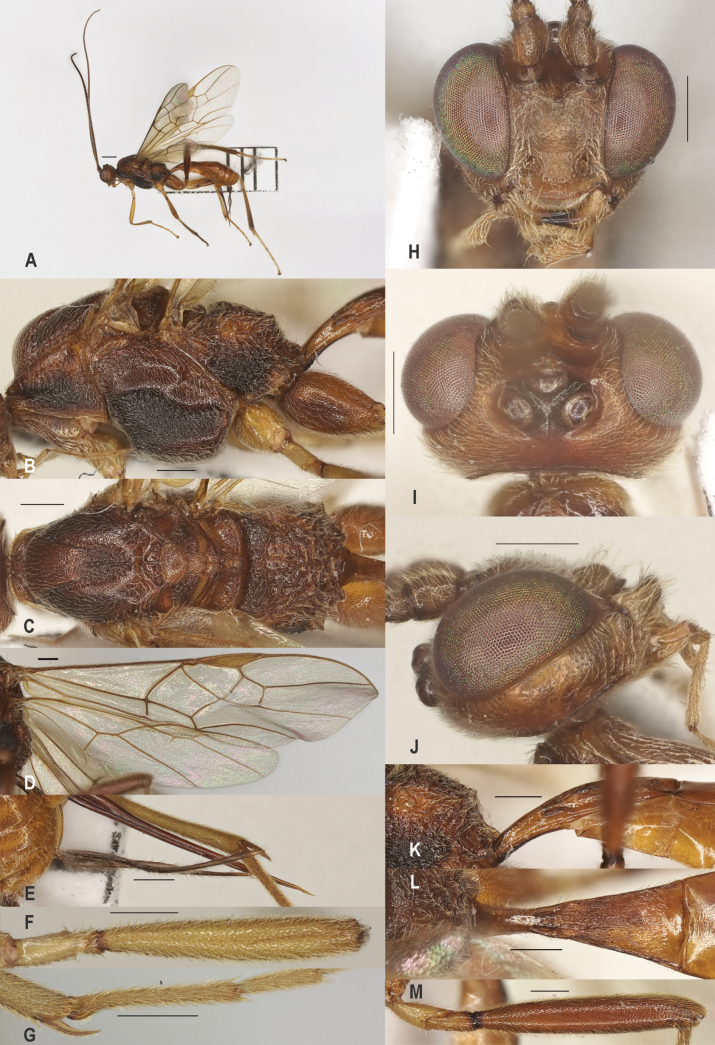
*Zeleirregularis* sp. nov., holotype, ♀ A. Habitus, lateral aspect; B. Mesosoma, lateral aspect; C. Mesosoma, dorsal aspect; D. Wings; E. Ovipositor sheath; F. Fore femur, lateral aspect; G. Fore tibial spur and fore basitarsus; H. Head, anterior aspect; I. Head, dorsal aspect; J. Head, lateral aspect; K. First metasomal tergite, lateral aspect; L. First metasomal tergite, dorsal aspect; M. Hind femur, lateral aspect. Scale bars: 1000 μm (A); 500 μm (B–M).

Propodeum with coarse and irregular sculpture including subbasal carina (Fig. 19C, B) propodeum oblique anteriorly in front of subbasal carina (Fig. 19B); fore femur comparatively robust, 6.0–6.3× as long as wide; mesosoma in lateral view usually more darkened contrasting with dorsal view; vein r of hind wing present as unsclerotised vein (Fig. 19D); first tergite 2.3–2.4× longer than its apical width; dorsope of first tergite medium-sized and area between dorsope wider than dorsope, basal part of first tergite in front of dorsope largely smooth (Fig. 19L); hind tarsus mainly white; ovipositor sheath ~0.27× as long as fore wing; vein 1r-m of hind wing comparatively long, vein 1r-m of hind wing 1.6–2.0× as long as vein 1-M; fore tibial spur medium-sized (Fig. 19G).

##### Comparative diagnosis.

Very similar to *Z.albiditarsus* but differs mainly by the subbasal carina of propodeum removed from anterior margin of propodeum (close to anterior margin of propodeum in *Z.albiditarsus*), the less developed fore spur, 0.3–0.4× as long as fore basitarsus (more developed in *Z.albiditarsus*) and the propodeum with coarse and irregular sculpture including subbasal carina (less coarse in *Z.albiditarsus*).

##### Description.

Holotype, ♀, length of fore wing 9.5 mm, of body 10.0 mm, and antenna 1.3× as long as fore wing.

***Head*.** Antennal segments 47, third segment as long as fourth segment and third, fourth and penultimate segments 3.6×, 3.6×, and 1.8× longer than wide, respectively; length of maxillary palp 1.6× longer than height of head; frons smooth and behind antennal sockets impressed; POL: diameter of posterior ocellus: OOL = 8: 10: 5; vertex convex, punctulate and densely setose (Fig. 19I); clypeus rather convex in lateral view, widely punctate (Fig. 19J); face widely smooth but punctulate near antennal sockets, it slightly narrowed ventrally, minimum width of face 1.4× height of face (Fig. 19H); length of eye 2.7× temple in dorsal view (Fig. 19I); length of malar space 0.3× basal width of mandible.

***Mesosoma*.** Length of mesosoma 1.7× its height; side of pronotum densely rugulose ventrally and posteriorly, sub-strigulate anteriorly; epicnemial area anteriorly rather smooth and matt, posteriorly punctate-rugose; precoxal sulcus narrowly crenulate, densely reticulate-punctate dorsally; dorsal of mesopleuron largely punctulate (Fig. 19B); mesosternum finely punctulate medially, punctate anteriorly; metapleuron roughly rugose; mesoscutal lobes densely punctulate; notauli rather widely crenulate, mesoscutum medio-posteriorly with a short media carina; scutellar sulcus deep and wide with one long median carina and four short carinae; scutellum rather convex and finely punctulate; metanotum with small smooth knob medio-posteriorly and without a median carina in front of it; propodeum reticulate-rugose, subbasal transverse carina of propodeum straight to curved posteriad, it irregular and laterally reduced, comparatively coarse; in lateral view propodeum gradually lowered posteriorly, posterior part not distinctly separated from antero-dorsal part distinctly (Fig. 19B, C).

***Wings*.** Fore wing (Fig. 19D): r:3-SR:SR1 = 8:20:95; 2-SR:3-SR: r-m = 17:20:14; cu-a vertical, interstitial. Hind wing (Fig. 19D): r present; M+CU:1-M = 80:15; 1r-m 1.6× 1-M.

***Legs*.** Hind coxa largely punctulate dorsally; length of fore femur 6.3× its width (Fig. 19F); length of fore tibial spur 0.3× fore basitarsus (Fig. 19G); lengths of hind femur and basitarsus 6.3× and 12.2× their widths, respectively (Fig. 19M).

***Metasoma*.** First tergite 2.3× longer than its apical width, its surface smooth, except some rugulosity behind spiracles; dorsope comparatively medium-sized, area behind dorsope depressed (Fig. 19L), laterope comparatively narrow but deep (Fig. 19K); second tergite mainly bare, smooth; ovipositor comparatively robust basally; length of ovipositor sheath 0.27× length of fore wing, sheath with moderately erect and short setae (Fig. 19E).

***Colour*.** Body brownish yellow; side of pronotum, mesosternum and metapleuron largely black; hind coxa and femur reddish brown; basal fourth of hind tibia nearly dark brown; hind tarsus white; veins C+SC+R, M+CU1, 1-M, cu-a and vein SR1 of fore wing dark brown, other veins and pterostigma yellowish; ovipositor sheath (except pale apex) dark brown.

***Variation*.** Vein 1r-m of hind wing 1.6–2.0× as long as vein 1-M; fore femur of ♀ 6.0–6.3× longer than wide; hind femur of ♀ 6.3–6.4× longer than wide; first metasomal tergite 2.3–2.4× its apical width.

##### Distribution.

China (Gansu, Henan, Jilin, Shaanxi).

##### Biology.

Unknown.

##### Etymology.

Named after the irregular subbasal carina; *in* and *regularis* is Latin for ‘not according to rule’.

#### ﻿ Zele peronatus

Taxon classificationAnimaliaHymenopteraBraconidae

(Shestakov, 1940), reinstated

BAF4459C-9C86-5BC7-89BE-5DCD187C8AE2

Fig. 20

Meteorus peronatus Shestakov, 1940: 16. van Achterberg 1979 (as junior synonym of Zeleniveitarsus (Cresson, 1872)).
Zele niveitarsis f. peronatus : van Achterberg 1979: 370; Chen et al. 1987: 94.

##### Type material examined.

***Holotype*.** Russia • ♀; Vladivostok, Sedanka; 10 Aug. 1930; R. Malaise leg.; 406, [19]77 (NRMS, Stockholm).

##### Other material examined.

China – **Fujian Prov.** • 1 ♀; Sangang; 27 Apr. 1984; Jia-zhuang Wang leg.; (ZJUH) No. 854298. – **Gansu Prov.** • 1 ♀; Pingliang; Jul. 1986; Shou-ming Liu leg.; (ZJUH) No. 865148. – **Jilin Prov.** • 1 ♀; Huangsongpu Forest Farm; 4 Aug. 2004; Yu-zhou Du, Zhi-jie Wang leg.; (ZJUH) No. 202401066. – **Shaanxi Prov.** • 1 ♀; Danfeng; 10 Aug. 2014; light trap; (ZJUH) No. 202401063. • 1 ♀; Liuba, Weituogou; alt. 1600 m; 21 Jun. 1998; Jun Chen leg; (ZJUH) No. 200104796. • 1 ♀; Ningshan, Xunyang; 12 Aug. 2013; Bing-bing Tu leg; light trap; (ZJUH) No. 201310084. • 1 ♀; Xunyangba; alt. 1485 m; 13 Aug. 2013; Bing-bing Tu leg; (ZJUH) No. 201300257. • 1 ♀; Xunyangba, Ningshan; 33.54°N, 108.55°E; alt. 1481 m; 17 Aug.–3 Oct. 2016; Qing-Qing Tan leg; Yellow and Green Malaise trap; (ZJUH) No. 202315006. • 1 ♀; Shanshuping, Lower Changqing Nature Reserve; 33.67°N, 107.57°E; alt. 1445 m; 25 Aug.–22 Sept. 2016; Qing-Qing Tan leg; Yellow Malaise trap; (ZJUH) No. 202315005. – **Zhejiang Prov.** • 2 ♀♀; Anji, Mt. Longwang; 31 Aug. 1993; Jun-hua He leg.; (ZJUH) Nos. 9310777, 9310778. • 1 ♀; Mt. Tianmu, Longfengjian; 27 Jul. 1999; Ming-shui Zhao leg.; (ZJUH) No. 200010956. GenBank accession no. PV356313.

##### Diagnosis.

**Figure 20.**
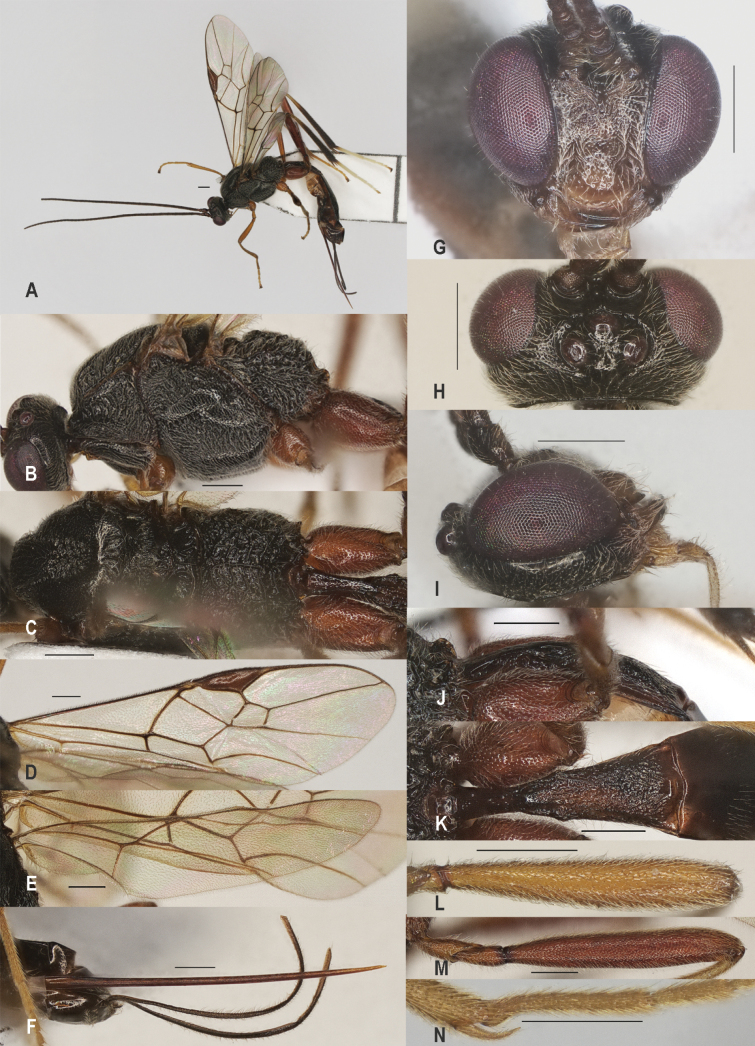
*Zeleperonatus* (Shestakov), China, Shaanxi, ♀ A. Habitus, lateral aspect; B. Mesosoma, lateral aspect; C. Mesosoma, dorsal aspect; D. Fore wing; E. Hind wing; F. Ovipositor sheath; G. Head, anterior aspect; H. Head, dorsal aspect; I. Head, lateral aspect; J. First metasomal tergite, lateral aspect; K. First metasomal tergite, dorsal aspect; L. Fore femur, lateral aspect; M. Hind femur, lateral aspect; N. Fore tibial spur and fore basitarsus. Scale bars: 500 μm.

Hind coxa punctate and with small smooth interspaces, rather shiny (Fig. 20A); pterostigma dark brown; dorsope of first tergite large and oval, area between dorsope slightly wider than dorsope and sculptured (Fig. 20K); hind tibia (except basal 1/4) dark brown or brown; frons largely smooth, rugulose or finely carinate (Fig. 20H); base of hind basitarsus often blackish brown, rarely infuscated. first tergite 2.3–2.8× its apical width; ovipositor sheath 0.46–0.50× as long as fore wing.

##### Distribution.

China (Fujian, Gansu, Jilin, Shaanxi, Zhejiang), Indonesia, Korea, Russia.

##### Biology.

Unknown.

##### Remarks.

This species was as a form included in the species *Z.niveitarsis* (van Achterberg 1979: figs 796–807), because of their whitish hind tarsus and eyes of ♀ comparatively large, 2.4–3.2× length of temple. However, the species is different from *Z.niveitarsis*, separable by the diffenences of face, dorsope, pterostigma and hind tibia. *Z.peronatus* have narrower face, ~1.0–1.1× wider than high (1.3× in *Z.niveitarsis*); comparatively smaller dorsope, area between dorsope slightly wider than dorsope (larger dorsope, area between dorsope smaller than dorsope in *Z.niveitarsis*); dark brown pterostigma (pale yellowish in *Z.niveitarsis*) and apical 1/3 of hind tibia dark brown (brownish yellow in *Z.niveitarsis*).

#### ﻿ Zele petiolatus

Taxon classificationAnimaliaHymenopteraBraconidae

Fang, van Achterberg & Chen sp. nov.

31881FDE-5C12-5BA8-9ED6-AB5EC733558C

https://zoobank.org/09A6B10D-8141-49DB-A3BC-5F3BB72AA91C

Fig. 21

##### Type material.

***Holotype*.** China – **Jiangxi Prov.** • ♀; Shangrao, Mt. Huanggang; 16 Aug. 2015; Ning Mao leg.; (ZJUH) No. 202401060. ***Paratype*.** China – **Hubei Prov.** • 1 ♀; Shennongjia, Muyuzhen, Tanbao River; 21 May 2012; Lu-jing Yang leg.; (ZJUH) No. 201203030. GenBank accession no. PV356310.

##### Diagnosis.

**Figure 21.**
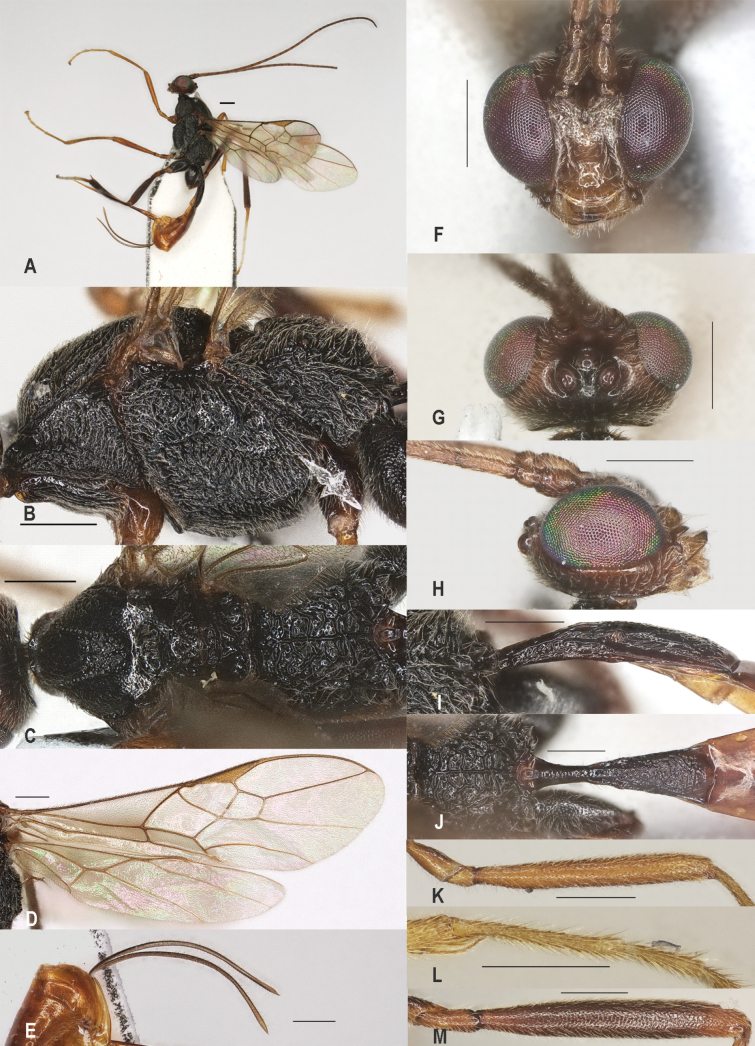
*Zelepetiolatus* sp. nov., holotype, ♀ A. Habitus, lateral aspect; B. Mesosoma, lateral aspect; C. Mesosoma, dorsal aspect; D. Wings; E. Ovipositor sheath; F. Head, anterior aspect; G. Head, dorsal aspect; H. Head, lateral aspect; I. First metasomal tergite, lateral aspect; J. First metasomal tergite, dorsal aspect; K. Fore femur, lateral aspect; L. Fore tibial spur and fore basitarsus; M. Hind femur, lateral aspect. Scale bars: 500 μm.

Pterostigma of ♀ pale brown (Fig. 21D); length of first tergite 2.7–2.9× its apical width (Fig. 21I, J); first metasomal tergite conspicuously narrowed in front of dorsope (Fig. 21J); dorsope of first tergite narrow in dorsal view and rather wide in lateral view, area between dorsope wider than dorsope and sculptured (Fig. 21I, J); hind tarsus white or ivory medially, distinctly contrasting with apex of hind tibia (Fig. 21A); setae of ovipositor sheath conspicuous and erect or semi-erect (Fig. 21E); hind tarsus mainly white; ovipositor sheath ~0.45× as long as fore wing.

##### Comparative diagnosis.

Similar to *Z.chlorophthalmus* but differs mainly by longer first metasomal tergite (shorter in *Z.chlorophthalmus*), white hind tarsus (yellowish in *Z.chlorophthalmus*) and erect or semi-erect setae of ovipositor sheath (less erect, more slanted in *Z.chlorophthalmus*).

##### Description.

Holotype, ♀, length of fore wing 5.8 mm, of body 6.9 mm, and antenna 1.3× as long as fore wing.

***Head*.** Antennal segments 38, third segment 0.9× shorter than fourth segment and third, fourth and penultimate segments 3.3×, 4.3×, and 2.0× longer than wide, respectively; maxillary palp broken; frons smooth and behind antennal sockets impressed; POL: diameter of posterior ocellus: OOL = 7: 6: 3; vertex finely punctulate and densely setose (Fig. 21G); clypeus convex in lateral view, weakly punctate (Fig. 21H); face medially punctate and near antennal sockets, it narrowed ventrally, minimum width of face 1.0× height of face (Fig. 21F); length of eye 2.7× temple in dorsal view (Fig. 21G); length of malar space 0.2× basal width of mandible.

***Mesosoma*.** Length of mesosoma 1.7× its height; side of pronotum largely reticulate-rugose and matt, with some striae ventrally and punctate antero-medially; epicnemial area mainly striate-rugose; precoxal sulcus coarsely reticulate-rugose dorsally, dorsal of mesopleuron largely smooth with few punctures (Fig. 21B); mesosternum double-punctate; metapleuron coarsely irregular rugose; mesoscutal lobes densely punctate and shiny; notauli rather widely crenulate, posteriorly widely crenulate-rugose; scutellar sulcus deep and wide, with a long median carina and two indistinct short carinae; scutellum slightly convex, weakly punctate; metanotum with small posterior knob and with one rather long carina; propodeum mainly areolate, subbasal carina complete, angulate, medio-longitudinal carina complete, and mostly well developed; propodeum gradually lowered posteriorly except dorsal part in front of subbasal carina, dorsal part comparatively large and mainly densely rugose-reticulate (Fig. 21B, C).

***Wings*.** Fore wing (Fig. 21D): r:3-SR:SR1 = 8:21:99; 2-SR:3-SR: r-m = 19:21:14; 1-CU1:2-CU1 = 1:30; cu-a nearly vertical, postfurcal. Hind wing (Fig. 21D): r absent; M+CU:1-M = 65:20; 1r-m 1.7× 1-M.

***Legs*.** Hind coxa largely punctate dorsally; length of fore femur 8.2× its width (Fig. 21K); length of fore tibial spur 0.3× fore basitarsus (Fig. 21L); lengths of hind femur and basitarsus 7.4× and 11.9× their widths, respectively (Fig. 21M).

***Metasoma*.** First tergite 2.9× longer than its apical width, it narrow medially, narrow petiolate part approximately as long as remaining posterior part and posterior part comparatively narrow, its surface striate and basally rugose but shiny; dorsope elliptical and comparatively narrow, area behind dorsope depressed (Fig. 21J), laterope large and sublateral (Fig. 21I); second tergite mainly bare, smooth and matt; ovipositor comparatively slender basally; length of ovipositor sheath 0.45× as long as fore wing, sheath with short semi-erect setae (Fig. 21E).

***Colour*.** Mesosoma, hind coxa and first metasomal tergite mainly black; head, antenna, and legs except hind leg reddish brown; hind tarsus largely white, but its telotarsus dorsally and base of basitarsus orange brown; hind tibia (except apical 1/6) and hind femur dark brown; veins and pterostigma pale brown; wings subhyaline with slight infuscation; apex of ovipositor sheath pale yellow.

***Variation*.** Vein 1r-m of hind wing 2.0–2.7× as long as vein 1-M; fore femur of ♀ 7.7–8.2× longer than wide; hind femur of ♀ 7.2–7.4× longer than wide; first metasomal tergite 2.7–2.9× its apical width. Antennal segments of ♂ unknown.

##### Distribution.

China (Hubei, Jiangxi).

##### Biology.

Unknown.

##### Etymology.

Named after the conspicuously narrowed base of the first tergite, *petiolatus* is Latin for stalk, stem.

#### ﻿ Zele romani

Taxon classificationAnimaliaHymenopteraBraconidae

(Fahringer, 1929), reinstated

63818F9A-1945-5941-8BC7-322579F8CFCC

Fig. 22

Meteorus romani Fahringer, 1929: 1–12. Syn. by van Achterberg 1979 with Zelealbiditarsus Curtis, 1832.
Zele albiditarsus f. albiditarsus : van Achterberg 1979: 380–383.

##### Type material examined.

***Lectotype*** of *Zeleromani*. Russia • ♀; “Kamtschatka Malaise”; “1231”; “Type”; “403, 77”; NHRS-HYME 000005237; (NRMS, Stockholm).

##### Other material examined.

None in this study.

##### Diagnosis.

**Figure 22.**
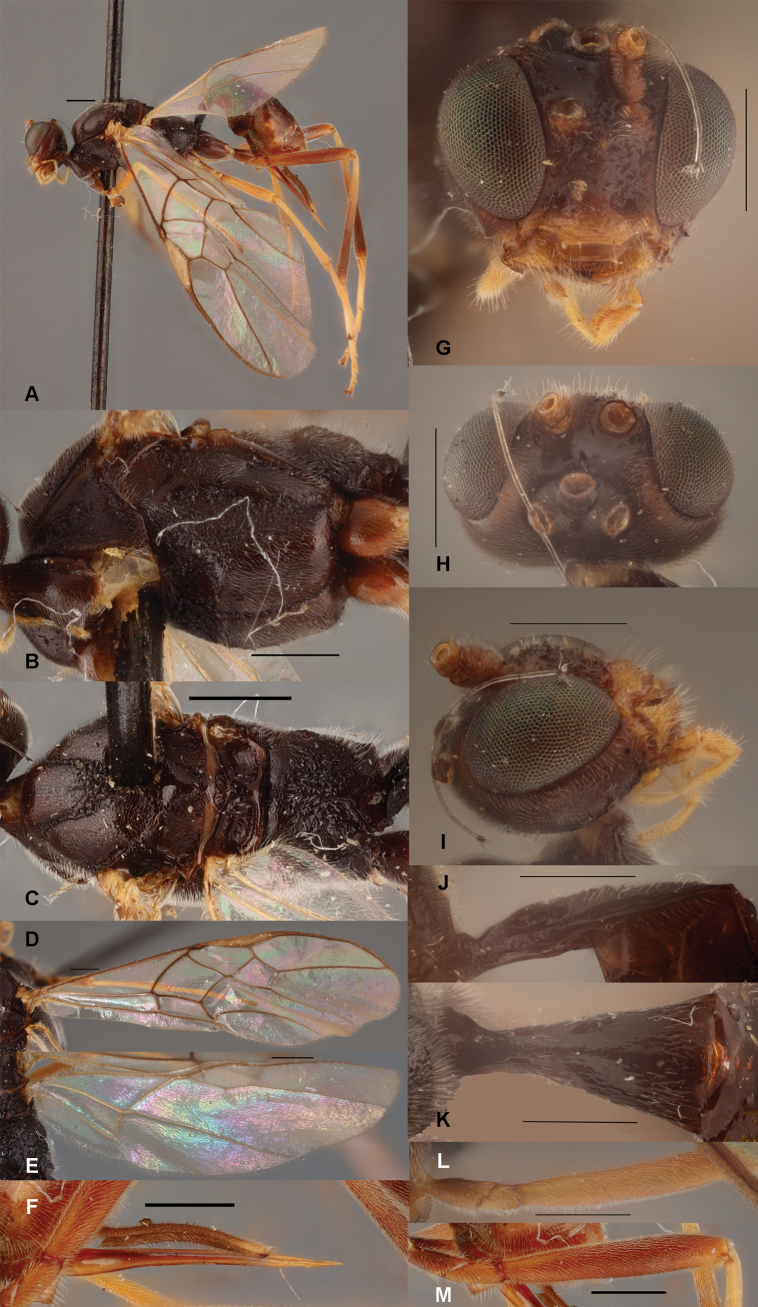
*Zeleromani* Fahringer, lectotype, Russia, ♀ A. Habitus, lateral aspect; B. Mesosoma, lateral aspect; C. Mesosoma, dorsal aspect; D. Fore wing; E. Hind wing; F. Ovipositor sheath; G. Head, anterior aspect; H. Head, dorsal aspect; I. Head, lateral aspect; J. First metasomal tergite, lateral aspect; K. First metasomal tergite, dorsal aspect; L. Fore femur, lateral aspect; M. Hind femur, lateral aspect. Scale bars: 500 μm. Photos: H. Vårdal.

Vein 1r-m of hind wing ~7.0× longer than vein 1-M (Fig. 22E); first metasomal tergite comparatively robust (~2.0× longer than its apical width), dorsope comparatively close to base of tergite and largely smooth, dorsope of first tergite small in dorsal view, area between dorsope much wider than dorsope (Fig. 22J, K); head largely dark brown (except yellowish clypeus and mandible) (Fig. 22G, H); precoxal sulcus area mainly coarsely punctate; ovipositor sheath ~0.45× as long as fore wing; hind tarsus largely ivory or white; length of malar space 0.2× basal width of mandible; pedicellus yellowish; hind coxa, first and second tergites dark brown; length of hind femur ~6.3× as long as wide (lectotype from photo); length of eye 2.3–2.4× temple.

##### Distribution.

Russia (Kamchatka Peninsula).

##### Biology.

Unknown.

##### Remarks.

This species was as a synonym included in the species Z.albiditarsusf.albiditarsus (van Achterberg, 1979), because of their length of vein 1-M of hind wing 0.3–0.8× vein cu-a; hind tarsus whitish, paler than hind femur; However, they can separated by having vein 1r-m of hind wing ~7.0× longer than vein 1-M (much shorter in *Z.albiditarsus*); less developed fore spur, 0.3–0.4× as long as fore basitarsus (more developed in *Z.albiditarsus*); vein r of hind wing absent (present in *Z.albiditarsus*) and largely dark brown head (yellowish in *Z.albiditarsus*).

#### ﻿ Zele rufulus

Taxon classificationAnimaliaHymenopteraBraconidae

(Thomson, 1895), reinstated

A2054146-6DD3-5344-9FC6-8B46C2F7368E

Fig. 23

Meteorus (Zemiotes) rufulus Thomson, 1895: 2149. Syn. by van Achterberg 1979 of Zelealbitarsusf.deceptor (Wesmael, 1835).
Zele albitarsus f. deceptor : van Achterberg 1979: 376.
Zele deceptor f. rufulus : van Achterberg 1984: 110–112; Chen et al. 1987: 94–96.

##### Type material examined.

***Neotype*** (here designated). Germany • ♀; Spessart, Nöhe von Lochmühle; 22–23 Aug. 1970; G. van Rossem leg.; RMNH (Leiden).

##### Diagnosis.

**Figure 23.**
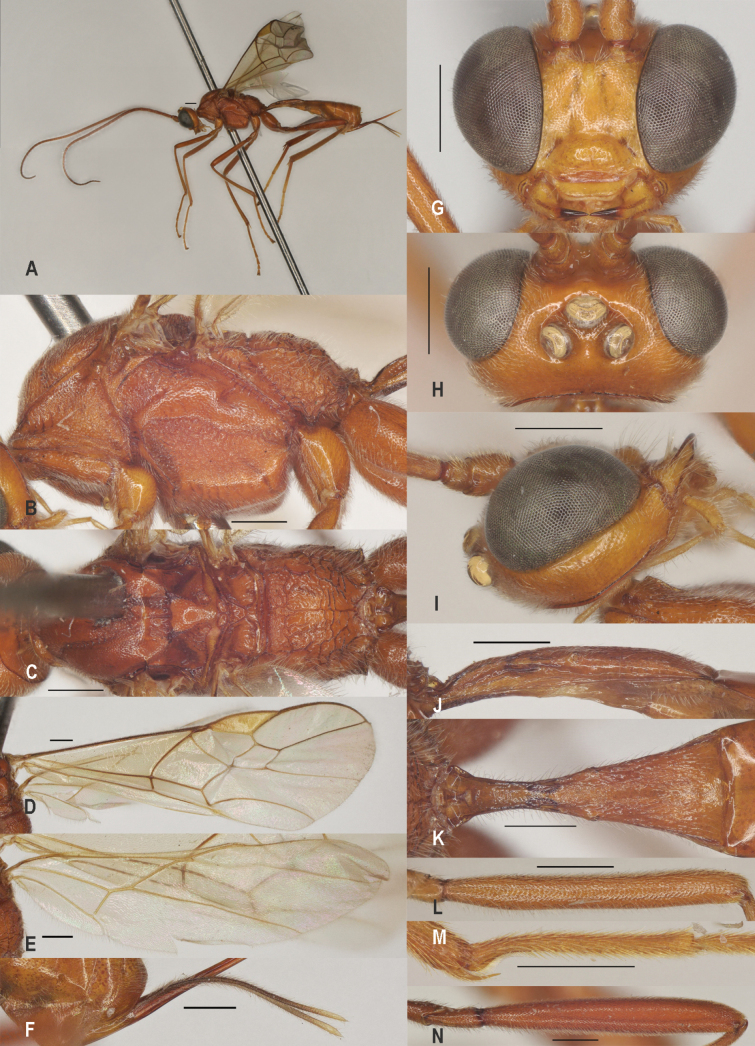
*Zelerufulus* (Thomson), Germany, neotype, ♀ A. Habitus, lateral aspect; B. Mesosoma, lateral aspect; C. Mesosoma, dorsal aspect; D. Fore wing; E. Hind wing; F. Ovipositor sheath; G. Head, anterior aspect; H. Head, dorsal aspect; I. Head, lateral aspect; J. First metasomal tergite, lateral aspect; K. First metasomal tergite, dorsal aspect; L. Fore femur, lateral aspect; M. Fore tibial spur and fore basitarsus; N. Hind femur, lateral aspect. Scale bars: 500 μm.

Subbasal carina of propodeum medially subparallel with anterior margin of propodeum (somewhat wider laterally) and enclosed area comparatively wide and rugose (Fig. 23C); vein 1-M of hind wing distinctly wider than vein M+CU (Fig. 23E); hind tarsus largely whitish; first tergite 2.4× longer than its apical width; dorsope of first tergite medium-sized and area between dorsope distinctly wider than dorsope (Fig. 23K); ovipositor sheath ~0.27× as long as fore wing.

##### Distribution.

Albania, Austria, China (Anhui, Fujian, Guizhou, Hubei, Hunan, Shaanxi, Sichuan, Xizang, Yunnan, Zhejiang), Germany, Japan, Lithuania, Russia, Sweden, former Yugoslavia.

##### Biology.

Parasitoid of Geometridae.

##### Remarks.

This species as a form was included in the species Z.deceptorf.rufulus (van Achterberg, 1984), because length of fore femur 6.2–8.4× its maximum width; length of fore spur comparatively short, vein r of hind wing absent, fit with Z.deceptorf.deceptor. It is only different by the colour of the hind tarsus and the sculpture of the propodeum. Although we obtained partial COI gene sequences, we failed to acquire COI data for *Z.rufulus*. Through a comprehensive study of the genus *Zele* of China and comparative morphological analysis, we conclude that these two taxa are different species. They can be separated by subbasal carina of propodeum medially subparallel with anterior margin of propodeum (distinct diverging from anterior margin in *Z.deceptor*); vein 1-M of hind wing distinctly wider than vein M+CU (as wide as in *Z.deceptor*), and hind tarsus largely whitish (usually brownish yellow or yellowish brown in *Z.deceptor*).

#### ﻿ Zele rugulosus

Taxon classificationAnimaliaHymenopteraBraconidae

Fang, van Achterberg & Chen sp. nov.

3344B3E5-8BA3-524D-88DB-737AC698DC87

https://zoobank.org/8A80F651-B244-4204-971D-6A6F3FAE2518

Fig. 24

##### Type material.

***Holotype*.** China – **Sichuan Prov.** • ♀; Ganzizangzu Zizhizhou, Luding, Gongkou; 27 Jul. 2005; Jiang-li Tan leg.; (ZJUH) No. 202401049. ***Paratypes*.** China – **Sichuan Prov.** • 2 ♀♀; Ganzizangzu Zizhizhou, Luding, Moxi; 19 Jun. 2005; light trap; (ZJUH) Nos. 202401087, 202401088. GenBank accession no. PV356299, PV356326, PV356327.

##### Diagnosis.

**Figure 24.**
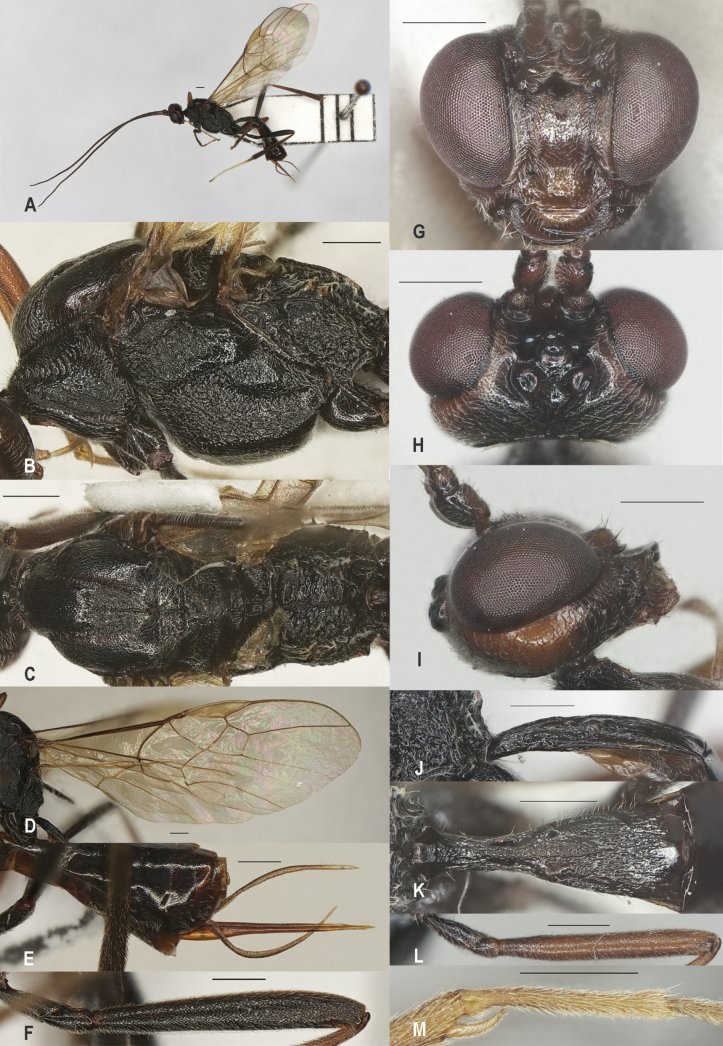
*Zelerugulosus* sp. nov., holotype, ♀ A. Habitus, lateral aspect; B. Mesosoma, lateral aspect; C. Mesosoma, dorsal aspect; D. Wings; E. Ovipositor sheath; F. Hind femur, lateral aspect r; G. Head, anterior aspect; H. Head, dorsal aspect; I. Head, lateral aspect; J. First metasomal tergite, lateral aspect; K. First metasomal tergite, dorsal aspect; L. Fore femur, lateral aspect; M. Fore tibial spur and fore basitarsus. Scale bars: 500 μm.

Propodeum depressed posteriorly in lateral view (Fig. 24B); mandible blackish (Fig. 24I); first tergite more widened posteriorly (compared to its minimum width: Fig. 24K); pterostigma pale yellowish, at most posterior rim slightly infuscated (Fig. 24D); hind tarsus whitish yellow; laterope narrow elliptical (Fig. 24K); lateral lobe of mesoscutum shiny; metanotum with 2 long carinae; mesoscutum medio-posteriorly with medium-sized carina (Fig. 24C); first tergite robust posteriorly and anteriorly comparatively abruptly narrowed (Fig. 24K), ~2.2–2.3× longer than its apical width; dorsope of first tergite long and moderately wide, area between dorsope distinctly wider than dorsope (Fig. 24K); ovipositor sheath ~0.19× as long as fore wing.

##### Comparative diagnosis.

Very similar to *Z.impolitus* but differs mainly by pale yellowish pterostigma (partly infuscated in *Z.impolitus*), anteriorly propodeum remotely sculptured between carinae (superficially sculptured in *Z.impolitus*) and metanotum with two rather long carinae dorsally and without knob posteriorly (with three medium-sized carinae dorsally and knob keeled posteriorly in *Z.impolitus*).

##### Description.

Holotype, ♀, length of fore wing 9.0 mm, of body 9.0 mm and antenna 1.1× as long as fore wing.

***Head*.** Antennal segments 45, third segment 1.1× longer than fourth segment and third, fourth and penultimate segments 3.8×, 3.2×, and 1.8× longer than wide, respectively; length of maxillary palp 1.3× longer than height of head; frons smooth and behind antennal sockets impressed; POL: diameter of posterior ocellus: OOL = 8: 8: 5; vertex convex, punctulate and densely setose; clypeus rather convex in lateral view, widely punctate dorsally, smooth ventrally (Fig. 24I); face largely smooth, rugulose near antennal sockets and eyes, and narrowed ventrally (Fig. 24G), minimum width of face 1.2× height of face; length of eye 2.1× temple in dorsal view (Fig. 24H); length of malar space 0.3× basal width of mandible.

***Mesosoma*.** Length of mesosoma 1.6× its height; side of pronotum striate-rugose ventrally, reticulate-rugose medially, smooth postero-dorsally; epicnemial area largely rugulose; precoxal sulcus widely crenulate-rugulose dorsally, narrowly punctulate ventrally, posteriorly narrowly smooth; dorsal of mesopleuron largely smooth and shiny, finely punctulate (Fig. 24B); mesosternum finely punctulate; metapleuron mainly smooth and shiny anteriorly, postero-ventrally rugose, postero-dorsally smooth; mesoscutal lobes widely punctulate and comparatively shiny; notauli anteriorly finely and narrowly crenulate, mesoscutum medio-posteriorly narrowly crenulate-rugose and with a long carina; scutellar sulcus deep and rather wide with one long obvious median carina; scutellum rather convex and finely punctulate; metanotum with small smooth knob medio-posteriorly, with two long carinae in front of knob; propodeum widely reticulate-rugose, subbasal carina of propodeum absent or fragmented submedially, anterior part of propodeum rather smooth anteriorly; propodeum with long straight median carina, and comparatively flat in lateral view and comparatively long; propodeum gradually lowered posteriorly, and posterior part not distinctly separated from antero-dorsal part distinctly (Fig. 24B, C).

***Wings*.** Fore wing (Fig. 24D): r:3-SR:SR1 = 9:23:120; 2-SR:3-SR: r-m = 26:23:17; 1-CU1:2-CU1 = 3:86; cu-a vertical, postfurcal. Hind wing (Fig. 24D): r absent; M+CU:1-M = 80:12; 1r-m 3.4× 1-M.

***Legs*.** Hind coxa densely punctate dorsally; length of fore femur 8.2× its width (Fig. 24L); length of fore tibial spur 0.3× fore basitarsus (Fig. 24M); lengths of hind femur and basitarsus 7.5× and 9.0× their widths, respectively (Fig. 24F).

***Metasoma*.** First tergite 2.2× longer than its apical width, its surface largely irregularly rugulose, only narrowly smooth posteriorly near second tergite; dorsope narrow elliptical and comparatively small, area behind dorsope depressed (Fig. 24K), laterope comparatively small but deep (Fig. 24J); second tergite mainly bare, smooth; ovipositor comparatively robust basally; length of ovipositor sheath 0.19× length of fore wing, sheath with short semi-erect setae (Fig. 24E).

***Colour*.** Antenna, vertex, mesosoma, coxae, trochanters of all legs, hind femur, hind tibia (except dark brown basal of 1/3), and metasoma largely black; head, mandible, fore and middle femora reddish brown; fore and middle tibias and tarsus yellowish brown; vein C+SC+R, vein 1-M and vein cu-a of fore wing, ovipositor sheath (except pale yellowish apex) mainly dark brown; hind tarsus largely whitish yellow, but its telotarsus dorsally and base of basitarsus dark brown; palpi and pterostigma pale yellowish.

***Variation*.** Vein 1r-m of hind wing 3.3–4.0× as long as vein 1-M; fore femur of ♀ 7.9–8.3× longer than wide; hind femur of ♀ 7.0–7.5× longer than wide; first metasomal tergite 2.2–2.3× its apical width.

##### Distribution.

China (Sichuan).

##### Biology.

Unknown.

##### Etymology.

Named after the very finely and densely rugulose precoxal sulcus; *rugulosus* is Latin for finely creased.

#### ﻿ Zele ruricola

Taxon classificationAnimaliaHymenopteraBraconidae

Maetô, 1986

1629288A-C15A-5D15-BF83-2B341575995A

Fig. 25

Zele ruricola Maetô, 1986: 253; Chen et al. 1987: 94.

##### Other material examined.

China – **Fujian Prov.** • 1 ♀; Dehua, Dongli, Mt. Xiaodaiyun; 6 Jun. 1960; Cheng-lin Ma leg.; (ZJUH) No. 871667. – **Heilongjiang Prov.** • 1 ♀; Liangshui; 7 Jul. 1977; Jun-hua He leg.; (ZJUH) No. 770470. – **Jilin Prov.** • 2 ♀♀; Mt. Changbai; 9 Aug. 1977; Jun-hua He leg.; (ZJUH) Nos. 770571, 770640. • 1 ♀; Hunjiang; 2 Aug. 1983; Fa-shen Li leg.; (ZJUH) No. 200012131. – **Zhejiang Prov.** • 1 ♀; Anming; 8 Apr. 1980; Han-lin Chen leg.; (ZJUH) No. 907728. • 1 ♀; Longquan, Mt. Fengyang; 25–29 Jul. 2007; Jing-xian Liu leg.; (ZJUH) No. 200802957. • 1 ♀; Mt. Xitianmu, Xianrending; 23 Aug. 1998; Ming-shui Zhao leg.; Malaise trap; (ZJUH) No. 994527.

##### Diagnosis.

**Figure 25.**
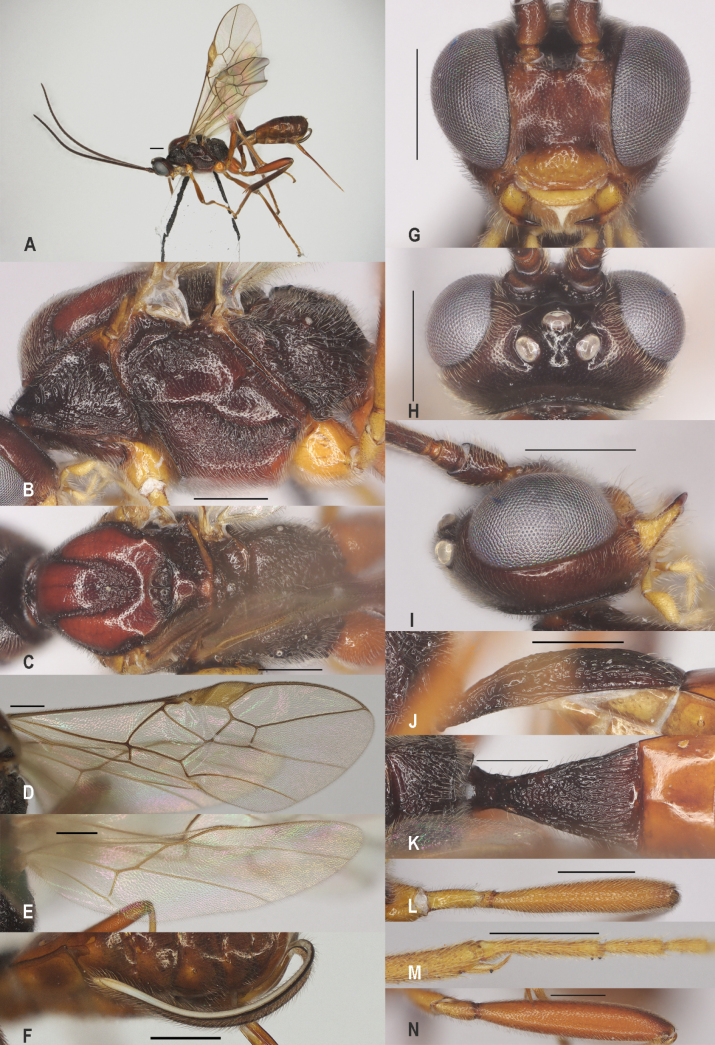
*Zeleruricola* Maetô, China, Zhejiang, ♀ A. Habitus, lateral aspect; B. Mesosoma, lateral aspect; C. Mesosoma, dorsal aspect; D. Fore wing; E. Hind wing; F. Ovipositor sheath; G. Head, anterior aspect; H. Head, dorsal aspect; I. Head, lateral aspect; J. First metasomal tergite, lateral aspect; K. First metasomal tergite, dorsal aspect; L. Fore femur, lateral aspect; M. Fore tibial spur and fore basitarsus; N. Hind femur, lateral aspect. Scale bars: 500 μm.

Length of hind femur of ♀ 5.0–6.2× its maximum width (Fig. 25L); length of first metasomal tergite 1.6–1.9× its apical width, robust (Fig. 25J, K); first metasomal tergite blackish and more or less contrasting with more or less yellowish (Fig. 25J); hind tarsus infuscated and apical 3/4 of hind tibia dark brown; pterostigma pale brown; dorsope of first tergite narrow and area between dorsope much wider than dorsope and area sculptured (Fig. 25K); ovipositor comparatively slender and setose part of sheath 0.34–0.40× as long as fore wing (Fig. 25F, D); notauli narrow and smooth or very finely crenulate anteriorly (Fig. 25C).

##### Distribution.

China (Fujian, Heilongjiang, Jilin, Zhejiang), Japan, Korea, Russia.

##### Biology.

Unknown.

##### Remarks.

As mentioned by Maetô (1986) this species is very similar to the European *Z.annulicrus* (Thomson, 1895), however, the difference in the head shape (dorsal view eye 2.0–2.5× longer than temple, but 1.4× in lectotype of *Z.annulicrus*). We recognise *Z.ruricola* as valid species because of the rectangular stemmaticum with larger ocelli (less so in *Z.annulicrus*), area below precoxal sulcus finely sculptured (mainly smooth in *Z.annulicrus*), swollen hind femur (slender) and subbasal carina of propodeum oblique (transverse, parallel with anterior margin of propodeum in *Z.annulicrus*: van Achterberg 1979: fig. 754) and dark brown scapus and pedicellus (yellowish in *Z.annulicrus*).

#### ﻿ Zele sculpticoxis

Taxon classificationAnimaliaHymenopteraBraconidae

Fang, van Achterberg & Chen sp. nov.

9E832CA7-50B8-5C8C-9A76-EAFDA5735428

https://zoobank.org/02F42CEF-E4C2-45B7-96AC-D938AE003285

Fig. 26

##### Type material.

***Holotype*.** China – **Hubei Prov.** • ♀; Shennongjia, Qianjiaping; 26 Aug. 1982; Jun-hua He leg.; (ZJUH) No. 825529. ***Paratypes*.** China –**Hubei Prov.** • 2 ♀♀; topotypic and same date; Nos. 825551, 825470; • 2 ♀♀; Shennongjia, Qianjiaping; 26 Aug. 1982; Shang-bo Shi leg.; (ZJUH) Nos. 870107, 870106. • 1 ♀; Shennongjia, Dashennongjia; 27 Aug. 1982; Jun-hua He leg.; (ZJUH) No. 825695. • 1 ♀; Jiuhu; 27 Aug. 1982; Shang-bo Shi leg.; (ZJUH) No. 870113. – **Shaanxi Prov.** • 1 ♀; Mt. Taibai; 20 Aug. 2003; Zai-fu Xu leg.; (ZJUH) No. 20059093.

##### Diagnosis.

**Figure 26.**
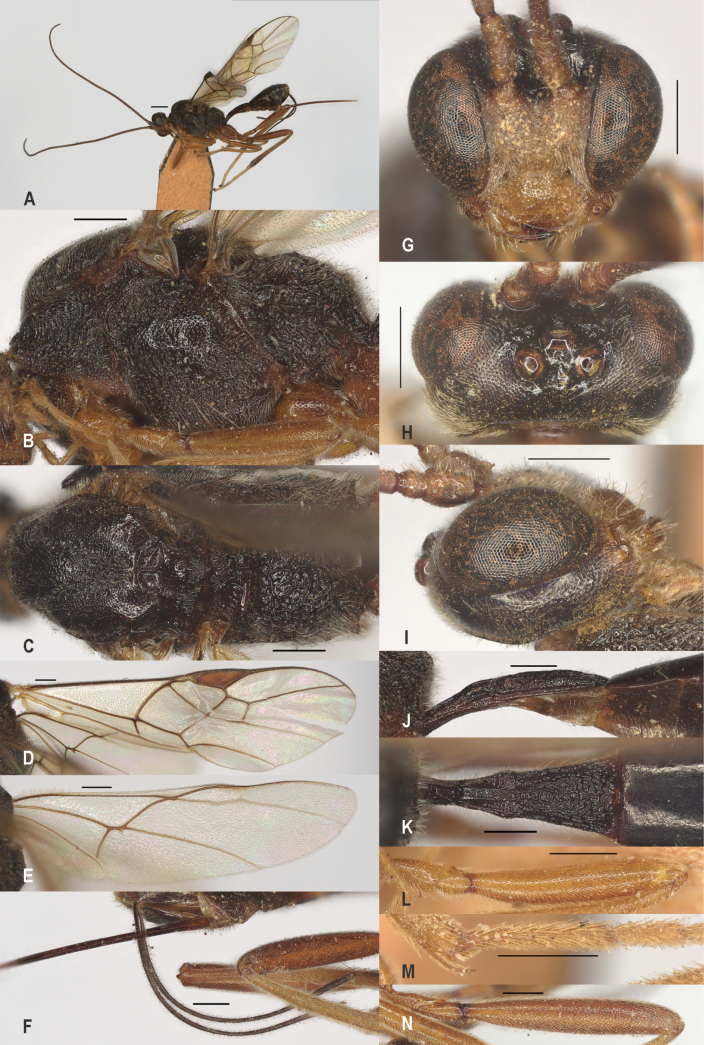
*Zelesculpticoxis* sp. nov., holotype, ♀ A. Habitus, lateral aspect; B. Mesosoma, lateral aspect; C. Mesosoma, dorsal aspect; D. Fore wing; E. Hind wing; F. Ovipositor sheath; G. Head, anterior aspect; H. Head, dorsal aspect; I. Head, lateral aspect; J. First metasomal tergite, lateral aspect; K. First metasomal tergite, dorsal aspect; L. Fore femur, lateral aspect; M. Fore tibial spur and fore basitarsus; N. Hind femur, lateral aspect. Scale bars: 500 μm.

Hind coxa densely punctate-rugulose, rather matt, and without distinct smooth interspaces (Fig. 26A); first tergite 2.4× longer than its apical width; hind tarsus mainly whitish, but base of basitarsus infuscated; dorsope of first tergite small and narrow elliptical, area between dorsope much wider than dorsope (Fig. 26K); hind tibia (except apical 1/3) ivory; frons usually partly rugose (Fig. 26G); base of hind basitarsus only infuscated; ovipositor sheath 0.45–0.58× as long as fore wing.

##### Comparative diagnosis.

Very similar to *Z.peronatus* but differs mainly by the hind coxa densely punctate-rugulose, rather matt, and without distinct smooth interspaces (punctate and with small smooth interspaces, rather shiny in *Z.peronatus*), small and narrow elliptical dorsope in first tergite (large and oval in *Z.peronatus*) and ivory hind tibia (except apical 1/3) (mostly dark brown or brown in *Z.peronatus*).

##### Description.

Holotype, ♀, length of fore wing 8.5 mm, of body 8.9 mm, and antenna 1.2× as long as fore wing.

***Head*.** Antennal segments 46, third segment 1.1× longer than fourth segment and third, fourth and penultimate segments 3.8×, 3.5×, and 2.0× longer than wide, respectively; length of maxillary palp 1.2× longer than height of head; frons sculptured and behind antennal sockets impressed; POL: diameter of posterior ocellus: OOL = 9: 9: 6; vertex convex, distinctly punctate and densely setose (Fig. 26H); clypeus rather convex in lateral view, largely smooth, only punctate medially (Fig. 26I); face largely punctate and matt, rugulose near antennal sockets, and slightly narrowed ventrally (Fig. 26G), minimum width of face 1.3× height of face; length of eye 2.2× temple in dorsal view (Fig. 26H); length of malar space 0.2× basal width of mandible.

***Mesosoma*.** Length of mesosoma 1.8× its height; side of pronotum largely reticulate-rugose, narrowly punctate posteriorly; epicnemial area reticulate-rugose; precoxal sulcus densely reticulate-rugose dorsally, narrowly reticulate-punctate ventrally; mesopleuron densely triple-punctate (Fig. 26B); mesosternum punctulate and shiny; dorsal of metapleuron mainly rugose, postero-dorsally canaliculate; mesoscutal lobes punctate and comparatively shiny; notauli anteriorly widely and distinctly crenulate, mesoscutum medio-posteriorly narrowly crenulate-rugose and without longitudinal carina; scutellar sulcus deep and rather wide with a long median carina; scutellum rather convex and punctulate; metanotum with three short converging carinae; propodeum widely reticulate-rugose, subbasal carina of propodeum irregular submedially, anterior area comparatively conspicuously rugulose; propodeum without long straight median carina; in lateral view propodeum gradually lowered posteriorly, posterior of subbasal carina weakly separated from antero-dorsal part and robust (Fig. 26B, C).

***Wings*.** Fore wing (Fig. 26D): r:3-SR:SR1 = 10:32:145; 2-SR:3-SR: r-m = 30:32:26; 1-CU1:2-CU1 = 5:85; cu-a vertical, antefurcal. Hind wing (Fig. 26E): r present; M+CU:1-M = 75:10; 1r-m 3.7× 1-M.

***Legs*.** Hind coxa densely punctate-rugulose dorsally; length of fore femur 6.0× its width (Fig. 26L); length of fore tibial spur 0.4× fore basitarsus (Fig. 26M); lengths of hind femur and basitarsus 6.5× and 10.8× their widths, respectively (Fig. 26N).

***Metasoma*.** First tergite 2.4× longer than its apical width, its surface coarsely reticulate-rugose; dorsope elliptical and comparatively small, area in front of dorsope depressed (Fig. 26K), laterope comparatively narrow (Fig. 26J); second tergite mainly bare, smooth; ovipositor comparatively slender basally; length of ovipositor sheath 0.58× as long as fore wing, sheath with long semi-erect setae (Fig. 26F).

***Colour*.** Head in dorsal view, mesosoma, metasoma and ovipositor sheath largely black; face, clypeus, mandible (except black apex), palpi, fore and middle legs yellowish, hind coxa and femur reddish brown; antenna, pterostigma, veins C+SC+R, 1-M and cu-a of fore wings, mainly dark brown; hind tibia brown but apical 1/3 blackish; hind tarsus largely whitish yellow, but its telotarsus dorsally brown.

***Variation*.** Vein 1r-m of hind wing 3.6–4.2× as long as vein 1-M; fore femur of ♀ 5.5–6.0× longer than wide; hind femur of ♀ 6.5–6.7× longer than wide; first metasomal tergite 2.3–2.5× as long as its apical width.

##### Distribution.

China (Hubei, Shaanxi).

##### Biology.

Unknown.

##### Etymology.

Named after the densely punctate-rugulose hind coxa; *sculpticoxis* is Latin for sculptured coxa.

#### ﻿ Zele shaanxiensis

Taxon classificationAnimaliaHymenopteraBraconidae

Fang, van Achterberg & Chen sp. nov.

17858996-D7EA-565C-94F1-99371E94749A

https://zoobank.org/1FC9BFB1-F7AE-47B7-B524-3680EE054502

Fig. 27

##### Type material.

***Holotype*.** China – **Shaanxi Prov.**• ♀; Ningshan; 24 Sep. 1979; Bu-guang Jin leg.; (ZJUH) No. 791192.

##### Diagnosis.

**Figure 27.**
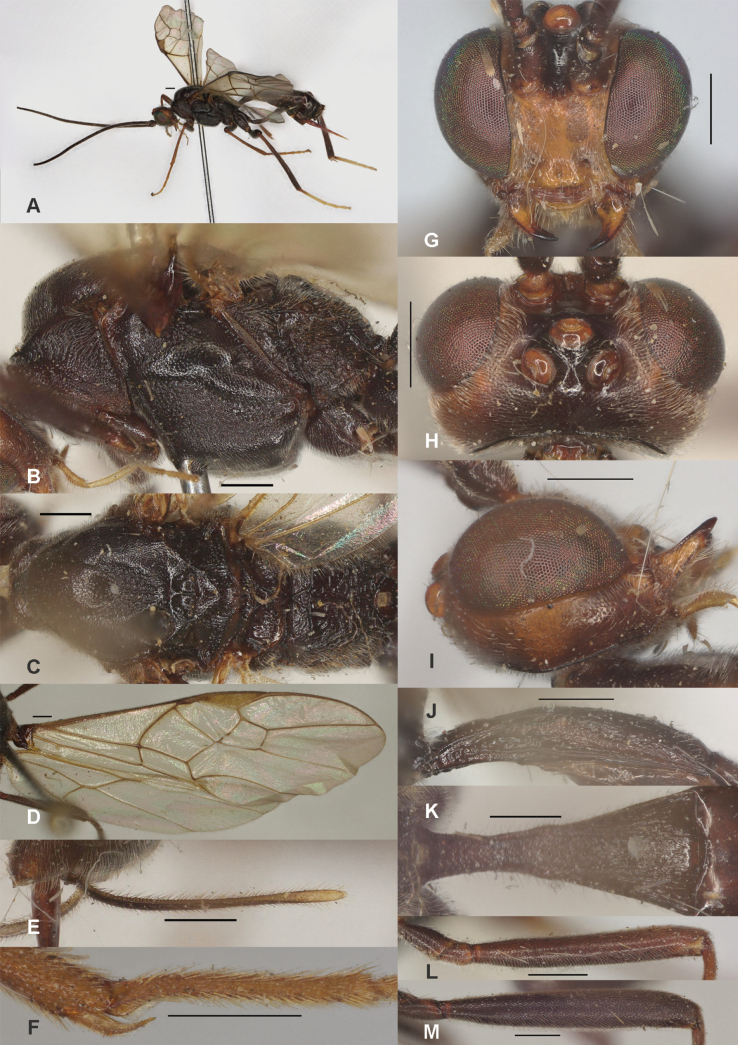
*Zeleshaanxiensis* sp. nov., holotype, ♀ A. Habitus, lateral aspect; B. Mesosoma, lateral aspect; C. Mesosoma, dorsal aspect; D. Fore wing; E. Hind wing; F. Ovipositor sheath; G. Head, anterior aspect; H. Head, dorsal aspect; I. Head, lateral aspect; J. First metasomal tergite, lateral aspect; K. First metasomal tergite, dorsal aspect; L. Fore femur, lateral aspect; M. Fore tibial spur and fore basitarsus; N. Hind femur, lateral aspect. Scale bars: 500 μm.

Eyes less protruding and temples less directly narrowed in dorsal view (Fig. 27H); vein m-cu of fore wing straight (Fig. 27D); anterior tentorial pits distinctly removed from eyes (Fig. 27G); second tergite dark brown basally; first metasomal tergite 2.2× its apical width, with comparatively small dorsope and area between dorsope much wider than dorsope; hind tarsus mainly white; ovipositor sheath ~0.21× as long as fore wing.

##### Comparative diagnosis.

Very similar to *Z.curvinervis* but differs mainly by the less protruding eyes (more protruding in *Z.curvinervis*), straight vein m-cu of fore wing (curved in *Z.curvinervis*) and anterior tentorial pits distinctly removed from eyes (close to eyes in *Z.curvinervis*).

##### Description.

Holotype, ♀, length of fore wing 9.3 mm, of body 8.9 mm.

***Head*.** Antenna incomplete, third segment nearly as long as fourth segment and third and fourth segments 2.7× and 2.5× longer than wide, respectively; length of maxillary palp 1.4× longer than height of head; frons smooth and behind antennal sockets impressed; POL: diameter of posterior ocellus: OOL = 9: 10: 5; vertex convex, punctulate and densely setose (Fig. 27H); clypeus slightly convex in lateral view, punctate apically (Fig. 27I); face widely smooth, it widened ventrally (Fig. 27G), minimum width of face 1.3× height of face; length of eye 2.2× temple in dorsal view (Fig. 27H); length of malar space 0.3× basal width of mandible.

***Mesosoma*.** Length of mesosoma 1.6× its height; side of pronotum reticulate-crenulate with few striae ventrally and crenulate posteriorly; epicnemial area rather rugulose; precoxal sulcus narrowly crenulate posteriorly, widely reticulate-rugulose; dorsal of mesopleuron punctate and shiny (Fig. 27B); mesosternum largely punctulate and shiny; metapleuron largely smooth anteriorly but striate-rugose posteriorly; mesoscutal lobes widely punctulate and shiny; notauli finely and narrowly crenulate, mesoscutum medio-posteriorly narrowly crenulate-rugose and with a long carina; scutellar sulcus deep and wide, with a long distinct median carina; scutellum slightly convex and weakly punctulate; metanotum with small smooth knob medio-posteriorly and with medium-sized carina in front of it; propodeum reticulate-rugose, subbasal transverse carina more straight and protruding, with comparatively large triangular area medially, area in front of subbasal transverse carina comparatively smooth; medio-longitudinal carina present; in lateral view propodeum gradually lowered posteriorly, posterior part not distinctly separated from antero-dorsal part distinctly (Fig. 27B, C).

***Wings*.** Fore wing (Fig. 27D): r:3-SR:SR1 = 12:30:123; 2-SR:3-SR: r-m = 25:30:20; cu-a oblique, interstitial. Hind wing (Fig. 27D): r absent; M+CU:1-M = 90:15; 1r-m 2.7× 1-M.

***Legs*.** Hind coxa largely punctulate dorsally; length of fore femur 7.0× its width (Fig. 27L); length of fore tibial spur 0.3× fore basitarsus (Fig. 27F); lengths of hind femur and basitarsus 7.0× and 9.6× their widths, respectively (Fig. 27M).

***Metasoma*.** First tergite 2.2× longer than its apical width, its surface coarsely reticulate-rugose; dorsope narrow and comparatively small, area in front of dorsope depressed (Fig. 27K), laterope comparatively narrow (Fig. 27J); second tergite mainly bare, smooth; ovipositor comparatively slender basally; length of ovipositor sheath 0.21× as long as fore wing, sheath with long semi-erect setae (Fig. 27E).

***Colour*.** Antenna mesosoma, all coxae, middle and hind femora, metasoma largely black; face, clypeus, mandible (except black apex) brownish yellow; pterostigma and palpi yellowish; fore and middle tibia and tarsus, ovipositor sheath (except pale apex) dark brown; hind tarsus largely whitish.

##### Distribution.

China (Shaanxi).

##### Biology.

Unknown.

##### Etymology.

Named after the province of the collection site, Shaanxi.

#### ﻿ Zele syntomus

Taxon classificationAnimaliaHymenopteraBraconidae

Fang, van Achterberg & Chen sp. nov.

4B74208E-8228-5DBB-8822-BA148E1F118C

https://zoobank.org/0D3F6B59-2DF0-42BC-9427-7DFEE9B45E2E

Fig. 28

##### Type material.

***Holotype*.** China – **Zhejiang Prov.** • ♀; Lishui, Qingyuan, Baishanzu; 25 Oct. 1996; Hong Wu leg.; (ZJUH) No. 945871. ***Paratypes*.** China – **Zhejiang Prov.** • 1 ♀; same collection data as for Holotype; (ZJUH) No. 945878. • 1 ♀; same collection data as fore preceding but different date; 20 Nov. 1993; (ZJUH) No. 946983. – **Taiwan Prov.** • 1 ♀; Mt. Xue; 15 Oct. 2009; Pu Tang leg.; (ZJUH) No. 201301734. – **Yunnan Prov.** • 1 ♀; Baoshan, Lujiangba, Mt. Gaoligong Natural Park; 10–11 May 2009; Jie Zeng leg.; (ZJUH) No. 201904147.

##### Diagnosis.

**Figure 28.**
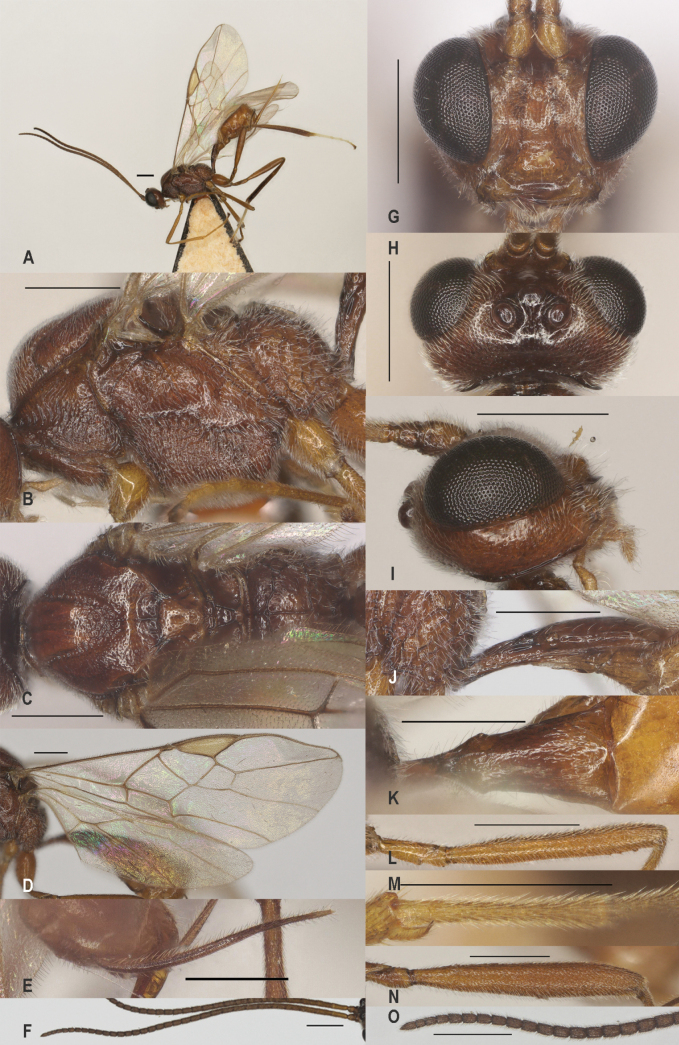
*Zelesyntomus* sp. nov., holotype, ♀ A. Habitus, lateral aspect; B. Mesosoma, lateral aspect; C. Mesosoma, dorsal aspect; D. Wings; E. Ovipositor sheath; F. Antenna; G. Head, anterior aspect; H. Head, dorsal aspect; I. Head, lateral aspect; J. First metasomal tergite, lateral aspect; K. First metasomal tergite, dorsal aspect; L. Fore femur, lateral aspect; M. Fore tibial spur and fore basitarsus; N. Hind femur, lateral aspect; O. Apical 1/3 of antenna. Scale bars: 500 μm.

Segments of apical 1/3 of antenna of ♀ shortened and ~1.4× longer than wide (Fig. 28O); first metasomal tergite 2.3–2.5× its apical width, it shiny and with comparatively large dorsope and area between dorsope wider than dorsope, part behind dorsope smooth or finely sculptured (Fig. 28K); hind coxa largely smooth or finely punctulate dorsally; pterostigma of ♀ pale yellowish (Fig. 28D); eyes length of eye 2.0–2.2× temple in dorsal view; hind tarsus mainly white; ovipositor sheath ~0.25× as long as fore wing.

##### Comparative diagnosis.

Similar to *Z.confusus* but differs mainly by having the segments of apical 1/3 of antenna ♀ shortened, ~1.4× longer than wide (longer, at least 2× in *Z.confusus*), largely smooth or finely punctulate hind coxa (densely punctate in *Z.confusus*) and brownish or yellowish hind femur (largely black in *Z.confusus*).

##### Description.

Holotype, ♀, length of fore wing 5.1 mm, of body 4.8 mm, and antenna 0.9× as long as fore wing.

***Head*.** Antennal segments 35, third segment as long as fourth segment and third, fourth, and penultimate segments 4.0×, 3.3×, and 1.2× longer than wide, respectively; length of maxillary palp 1.2× longer than height of head; frons smooth and behind antennal sockets impressed; POL: diameter of posterior ocellus: OOL = 6: 5: 4; vertex finely punctulate and densely setose (Fig. 28H); clypeus rather convex in lateral view, punctulate (Fig. 28I); face finely punctulate and shiny, minimum width of face 1.4× height of face (Fig. 28G); length of eye 2.0× temple in dorsal view (Fig. 28H); length of malar space 0.5× basal width of mandible.

***Mesosoma*.** Length of mesosoma 1.6× its height; side of pronotum shiny, slightly striate antero-medially, largely smooth, faintly punctulate posteriorly; epicnemial area rather smooth, postero-dorsally finely rugulose; precoxal sulcus rather widely reticulate-rugulose, postero-ventrally punctate (Fig. 28B); remainder of mesopleuron smooth and shiny; mesosternum punctulate; metapleuron spaced rugose; mesoscutal lobes superficially punctulate, interspaces smooth and shiny; scutellar sulcus deep and wide, with a long median carina; scutellum slightly convex, weakly punctulate; metanotum with small posterior knob and with a pair of rather long carinae; propodeum mainly areolate, subbasal carina complete, angulate, medio-longitudinal carina complete, and mostly well developed; propodeum gradually lowered posteriorly except dorsal part in front of subbasal carina, dorsal part comparatively large, mainly smooth and shiny (Fig. 28B, C).

***Wings*.** Fore wing (Fig. 28D): r:3-SR:SR1 = 8:25:131; 2-SR:3-SR: r-m = 25:25:15; 1-CU1:2-CU1 = 3:50; cu-a vertical, postfurcal. Hind wing (Fig. 28D): r absent; M+CU:1-M = 65:20; 1r-m 2.0× 1-M.

***Legs*.** Hind coxa largely smooth dorsally; length of fore femur 9.4× its width (Fig. 28L); length of fore tibial spur 0.3× fore basitarsus (Fig. 28M); lengths of hind femur and basitarsus 7.0× and 8.2× their widths, respectively (Fig. 28N).

***Metasoma*.** First tergite 2.1× longer than its apical width, smooth and shiny, part behind level of spiracles distinctly widened, dorsope elliptical and rather large, area behind dorsope depressed (Fig. 28K), laterope medium-sized and sublateral (Fig. 28J); second tergite mainly bare, smooth and strongly shiny; ovipositor comparatively slender basally; length of ovipositor sheath 0.25× as long as fore wing and sheath with long oblique setae (Fig. 28E).

***Colour*.** Head, mesosoma and first metasomal tergite mainly rather dark brown; palpi, antenna, and legs brown; hind tarsus largely white, but telotarsus dorsally and base of basitarsus brown; hind tibia (except apical 1/3) black; veins and pterostigma pale yellow; wings subhyaline with slight infuscation; apex of ovipositor sheath pale brown.

***Variation*.** Vein 1r-m of hind wing 1.9–2.3× as long as vein 1-M; fore femur of ♀ 7.5–9.4× longer than wide; hind femur of ♀ 7.0–7.4× longer than wide; first metasomal tergite 2.4–2.5× its apical width. Antennal segments of ♀ 35(3), 31(1); of ♂ unknown. ♀ (Yunnan) mainly yellow, except apical 1/3 of antenna brown, telotarsus and base of basitarsus of hind leg yellow.

##### Distribution.

China (Taiwan, Yunnan, Zhejiang).

##### Biology.

Unknown.

##### Etymology.

Named after the shortened antennal segments; *syntomos* is Greek for abridged.

#### ﻿ Zele vacatus

Taxon classificationAnimaliaHymenopteraBraconidae

Fang, van Achterberg & Chen sp. nov.

9B8C79D6-9A19-52B2-9BAA-8E1D57B9217F

https://zoobank.org/839AC532-4AD1-4329-80C2-267C82897CE4

Fig. 29

##### Type material.

***Holotype*.** China – **Yunnan Prov.** • ♀; Lijiang, Lashi; 18 Aug. 2003; Ting-jing Li leg.; (ZJUH) No. 20046094.

##### Diagnosis.

**Figure 29.**
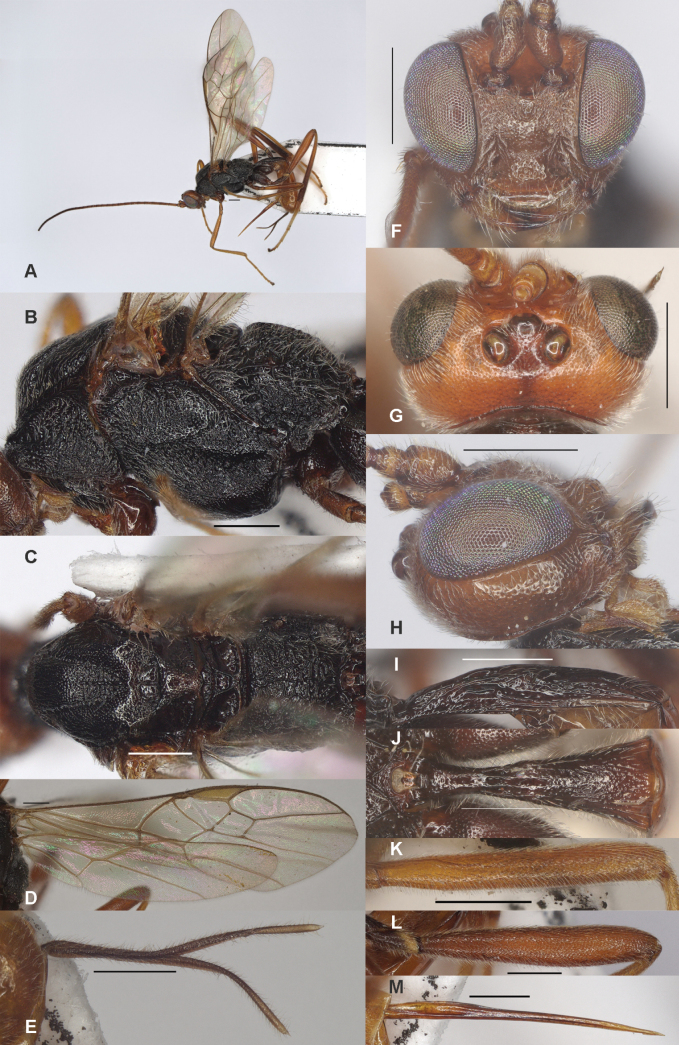
*Zelevacatus* sp. nov., holotype, ♀ A. Habitus, lateral aspect; B. Mesosoma, lateral aspect; C. Mesosoma, dorsal aspect; D. Wings; E. Ovipositor sheath; F. Head, anterior aspect; G. Head, dorsal aspect; H. Head, lateral aspect; I. First metasomal tergite, lateral aspect; J. First metasomal tergite, dorsal aspect; K. Fore femur, lateral aspect; L. Hind femur, lateral aspect; M. Ovipositor. Scale bars: 500 μm.

Hind femur robust, and basal part of ovipositor comparatively slender, maximum width of basal part of ovipositor 0.4× maximum width of hind femur (Fig. 29L, M); subbasal transverse carina of propodeum not discernible from surrounding sculpture (Fig. 29C); fore femur robust, ~7.4× as long as wide; hind femur reddish brown and apical 1/2 of hind tibia dark brown (Fig. 29A); first metasomal tergite 2.5× its apical width, it less shiny and with comparatively small dorsope and area between dorsope much wider than dorsope, part behind dorsope irregular and obvious rugose (Fig. 29K); hind tarsus mainly whitish yellow; ovipositor sheath ~0.26× as long as fore wing.

##### Comparative diagnosis.

Very similar to *Z.confusus* but differs mainly by the robust hind femur and slender basal part of ovipositor, maximum width of basal part of ovipositor 0.4× maximum width of hind femur (slender hind femur and robust basal part of ovipositor, 0.6× in *Z.confusus*), subbasal transverse carina of propodeum not discernible from surrounding sculpture (different from surrounding sculpture in *Z.confusus*), reddish brown hind femur (largely black in *Z.confusus*) and yellowish hind tarsus (largely white in *Z.confusus*).

##### Description.

Holotype, ♀, length of fore wing 6.7 mm, of body 7.1 mm.

***Head*.** Antenna incomplete, third segment nearly as long as fourth segment and third and fourth segments 4.0× and 4.2× longer than wide, respectively; length of maxillary palp 1.3× longer than height of head; frons smooth and behind antennal sockets impressed; POL: diameter of posterior ocellus: OOL = 8: 6: 4; vertex convex, punctulate and densely setose (Fig. 29H); clypeus rather convex in lateral view, punctate apically (Fig. 29I); face widely punctulate and smooth, it widened ventrally (Fig. 29G), minimum width of face 1.6× height of face; length of eye 1.4× temple in dorsal view(Fig. 29H); length of malar space 0.5× basal width of mandible.

***Mesosoma*.** Length of mesosoma 1.5× its height; side of pronotum reticulate-crenulate with few striae ventrally and punctate posteriorly; epicnemial area rather rugulose; precoxal sulcus widely reticulate-rugulose; reminder of mesopleuron punctate dorsally and reticulate-punctate ventrally (Fig. 29B); mesosternum finely punctulate and shiny; metapleuron smooth anteriorly but striate-rugose posteriorly; mesoscutal lobes punctate and less shiny; notauli widely crenulate, mesoscutum medio-posteriorly narrowly crenulate-rugose and with a long carina; scutellar sulcus shallow and wide, one long median carina distinctly; scutellum rather convex and weakly punctulate; metanotum with small smooth knob medio-posteriorly and with two long carina in front of it; propodeum reticulate-rugose, subbasal transverse carina of propodeum not discernible from surrounding sculpture, area in front if subbasal carina coarsely punctate; medio-longitudinal carina present; in lateral view propodeum gradually lowered posteriorly, posterior part not distinctly separated from antero-dorsal part distinctly (Fig. 29B, C).

***Wings*.** Fore wing (Fig. 29D): r:3-SR:SR1 = 13:35:130; 2-SR:3-SR: r-m = 30:35:25; cu-a vertical, interstitial. Hind wing (Fig. 29D): r absent; M+CU:1-M = 87:15; 1r-m 2.8× 1-M.

***Legs*.** Hind coxa largely punctate dorsally; length of fore femur 7.4× its width (Fig. 29K); length of fore tibial spur 0.3× fore basitarsus; lengths of hind femur and basitarsus 6.2× and 10.8× their widths, respectively (Fig. 29L).

***Metasoma*.** First tergite 2.5× longer than its apical width, its surface coarsely reticulate-rugose; dorsope elliptical and comparatively small, area in front of dorsope depressed (Fig. 29J), laterope comparatively narrow (Fig. 29I); second tergite mainly bare, smooth; ovipositor comparatively slender basally; length of ovipositor sheath 0.26× as long as fore wing, sheath with short semi-erect setae (Fig. 29E).

***Colour*.** Antenna (except black apical), head, mandible (except black apex), fore and middle legs (except black middle coxa), metasoma (except black first tergite) mainly orange yellow; mesosoma, hind coxa and trochanter largely black; palpi pale yellowish; pterostigma and ovipositor sheath (except pale apex) pale brown; hind femur reddish brown; apical 1/2 of hind tibia dark brown; hind tarsus largely whitish yellow, but its telotarsus dorsally yellow; wings subhyaline with some infuscation.

##### Distribution.

China (Yunnan).

##### Biology.

Unknown.

##### Etymology.

Named after the subbasal transverse carina of propodeum which is not discernible from surrounding sculpture; *vacatus* is Latin for empty.

## ﻿Discussion

In this comprehensive study of the Chinese species of *Zele*, a genus of koinobiont endoparasitoids of caterpillars, we adopted an integrative taxonomic approach by effectively merging morphological research with molecular phylogenetic analyses. Initial morphological assessments revealed subtle interspecific variations, but molecular characterisation uncovered phylogenetically informative diagnostic traits, including the carinae patterns on the mesoscutum and propodeum, as well as the ratios of vein 1r-m and vein 1-M of hind wing.

The COI sequences of DNA barcoding were used to investigate the intra- and interspecific variation within *Zele* through two different molecular species delimitation methods (ABGD and bPTP). It resulted in the recognition of 27 species, including 19 new species, an increase of ~70% of the valid species worldwide. The COI gene effectively captures species-level divergence in *Zele*, making it a suitable barcode marker for future systematic and ecological studies of this genus. At the moment 30 species are recognised (Yu et al. 2016), but most likely four species described from India (Sharma 1985) do not belong to *Zele*. The identification key to all Chinese species provided includes two extra species that likely occur in northeast China.

The bPTP results for *Zeleperonatus* suggest that the specimen GBAH12655-15, sourced from Japan, exhibits genetic differences when compared to the single specimen analysed in this study, which are from Shaanxi, China. Owing to the environmental disparities between island and mainland habitats, there is a limited genetic divergence at the COI sequences within this species. Furthermore, no reliable morphological diagnostic characters were found to distinguish the Japanese specimen from mainland individuals. Therefore, this level of variation does not support the classification of the Japanese specimen in this study as a separate new species. These findings show the efficacy of integrating COI barcode sequences with morphological analysis for species delimitation in taxa with numerous cryptic species. Nevertheless, we observed methodological discrepancies in species delimitation, as different analytical approaches yielded incongruent assessments of putative species and MOTUs (Macko et al. 2024). Specifically, we employed ABGD and bPTP to evaluate species delineations in *Zele*, which showed that these methods did not consistently delineate all studied *Zele* species into the same potential species or MOTUs. This methodological discordance strongly advocates for an integrated approach in species delimitation, particularly for taxa with extensive cryptic diversity. Consequently, we propose that future systematic investigations of *Zele* and similar groups should persist in combining multiple species delimitation methods to more accurately define species and reveal the diversity of species.

## Supplementary Material

XML Treatment for Zele

XML Treatment for Zele admirabilis

XML Treatment for Zele albiditarsus

XML Treatment for Zele aquilus

XML Treatment for Zele caligatus

XML Treatment for Zele carinatus

XML Treatment for Zele chinensis

XML Treatment for Zele chlorophthalmus

XML Treatment for Zele confusus

XML Treatment for Zele cristatus

XML Treatment for Zele curvatus

XML Treatment for Zele curvinervis

XML Treatment for Zele deceptor

XML Treatment for Zele densipunctatus

XML Treatment for Zele extensus

XML Treatment for Zele fulgidus

XML Treatment for Zele fuscatus

XML Treatment for Zele impolitus

XML Treatment for Zele inclinator

XML Treatment for Zele irregularis

XML Treatment for Zele peronatus

XML Treatment for Zele petiolatus

XML Treatment for Zele romani

XML Treatment for Zele rufulus

XML Treatment for Zele rugulosus

XML Treatment for Zele ruricola

XML Treatment for Zele sculpticoxis

XML Treatment for Zele shaanxiensis

XML Treatment for Zele syntomus

XML Treatment for Zele vacatus

## ﻿Additional information

### Use of AI

No use of AI was reported.

### Author ORCIDs

Yu Fang https://orcid.org/0009-0000-7685-8757

Cornelis van Achterberg https://orcid.org/0000-0002-6495-4853

Pu Tang https://orcid.org/0000-0003-2296-9544

Xue-xin Chen https://orcid.org/0000-0002-9109-8853

### Data availability

All of the data that support the findings of this study are available in the main text or Supplementary Information.

## Supplementary materials

Supplementary material 1 Information of terminal taxa of *Zele* molecular analysis Data type xlsx https://binary.pensoft.net/file/1388536This dataset is made available under the Open Database License (http://opendatacommons.org/licenses/odbl/1.0/). The Open Database License (ODbL) is a license agreement intended to allow users to freely share, modify, and use this Dataset while maintaining this same freedom for others, provided that the original source and author(s) are credited.Yu Fang, Cornelis van Achterberg, Pu Tang, Xue-xin Chen

Supplementary material 2 MEGA K2p distance Data type xlsx https://binary.pensoft.net/file/1388537This dataset is made available under the Open Database License (http://opendatacommons.org/licenses/odbl/1.0/). The Open Database License (ODbL) is a license agreement intended to allow users to freely share, modify, and use this Dataset while maintaining this same freedom for others, provided that the original source and author(s) are credited.Yu Fang, Cornelis van Achterberg, Pu Tang, Xue-xin Chen

Supplementary material 3 *Zele* align Data type fasta https://binary.pensoft.net/file/1388538This dataset is made available under the Open Database License (http://opendatacommons.org/licenses/odbl/1.0/). The Open Database License (ODbL) is a license agreement intended to allow users to freely share, modify, and use this Dataset while maintaining this same freedom for others, provided that the original source and author(s) are credited.Yu Fang, Cornelis van Achterberg, Pu Tang, Xue-xin Chen

Supplementary material 4 ABGD web results Data type png https://binary.pensoft.net/file/1388539This dataset is made available under the Open Database License (http://opendatacommons.org/licenses/odbl/1.0/). The Open Database License (ODbL) is a license agreement intended to allow users to freely share, modify, and use this Dataset while maintaining this same freedom for others, provided that the original source and author(s) are credited.Yu Fang, Cornelis van Achterberg, Pu Tang, Xue-xin Chen

Supplementary material 5
*Zele* ML
Data type treefile https://binary.pensoft.net/file/1388540This dataset is made available under the Open Database License (http://opendatacommons.org/licenses/odbl/1.0/). The Open Database License (ODbL) is a license agreement intended to allow users to freely share, modify, and use this Dataset while maintaining this same freedom for others, provided that the original source and author(s) are credited.Yu Fang, Cornelis van Achterberg, Pu Tang, Xue-xin Chen

Supplementary material 6 PTP species delimitation results MLP artition Data type png https://binary.pensoft.net/file/1388541This dataset is made available under the Open Database License (http://opendatacommons.org/licenses/odbl/1.0/). The Open Database License (ODbL) is a license agreement intended to allow users to freely share, modify, and use this Dataset while maintaining this same freedom for others, provided that the original source and author(s) are credited.Yu Fang, Cornelis van Achterberg, Pu Tang, Xue-xin Chen

Supplementary material 7 PTP species delimitation results likelihood trace plot Data type pdf https://binary.pensoft.net/file/1388542This dataset is made available under the Open Database License (http://opendatacommons.org/licenses/odbl/1.0/). The Open Database License (ODbL) is a license agreement intended to allow users to freely share, modify, and use this Dataset while maintaining this same freedom for others, provided that the original source and author(s) are credited.Yu Fang, Cornelis van Achterberg, Pu Tang, Xue-xin Chen

Supplementary material 8 *Zele* + outgroup align Data type fasta https://binary.pensoft.net/file/1388543This dataset is made available under the Open Database License (http://opendatacommons.org/licenses/odbl/1.0/). The Open Database License (ODbL) is a license agreement intended to allow users to freely share, modify, and use this Dataset while maintaining this same freedom for others, provided that the original source and author(s) are credited.Yu Fang, Cornelis van Achterberg, Pu Tang, Xue-xin Chen

Supplementary material 9 *Zele* + outgroup ML Data type treefile https://binary.pensoft.net/file/1388544This dataset is made available under the Open Database License (http://opendatacommons.org/licenses/odbl/1.0/). The Open Database License (ODbL) is a license agreement intended to allow users to freely share, modify, and use this Dataset while maintaining this same freedom for others, provided that the original source and author(s) are credited.Yu Fang, Cornelis van Achterberg, Pu Tang, Xue-xin Chen
