# Supplementary figures and images for: ﻿Revision of genus Zele Curtis (Hymenoptera, Braconidae, Euphorinae) from China, with description of nineteen new species
Source: Zookeys. 2025 Aug 5;1248:125–208. doi: 10.3897/zookeys.1248.158182 (PMC12344441; doi:10.3897/zookeys.1248.158182)

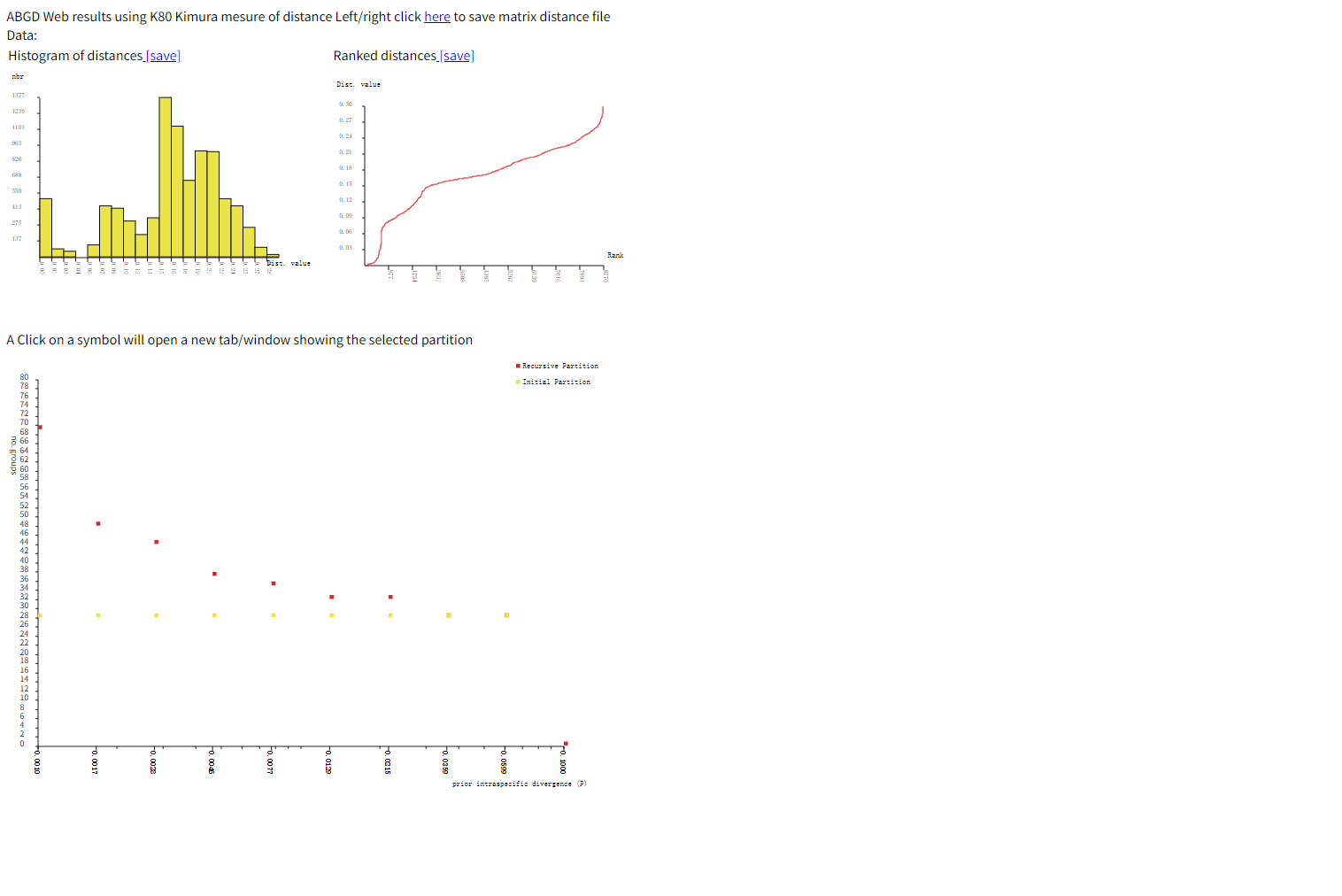

Supplement: Supplementary material 4 — ABGD web results [file zookeys-1248-125_article-158182__-s004.png]

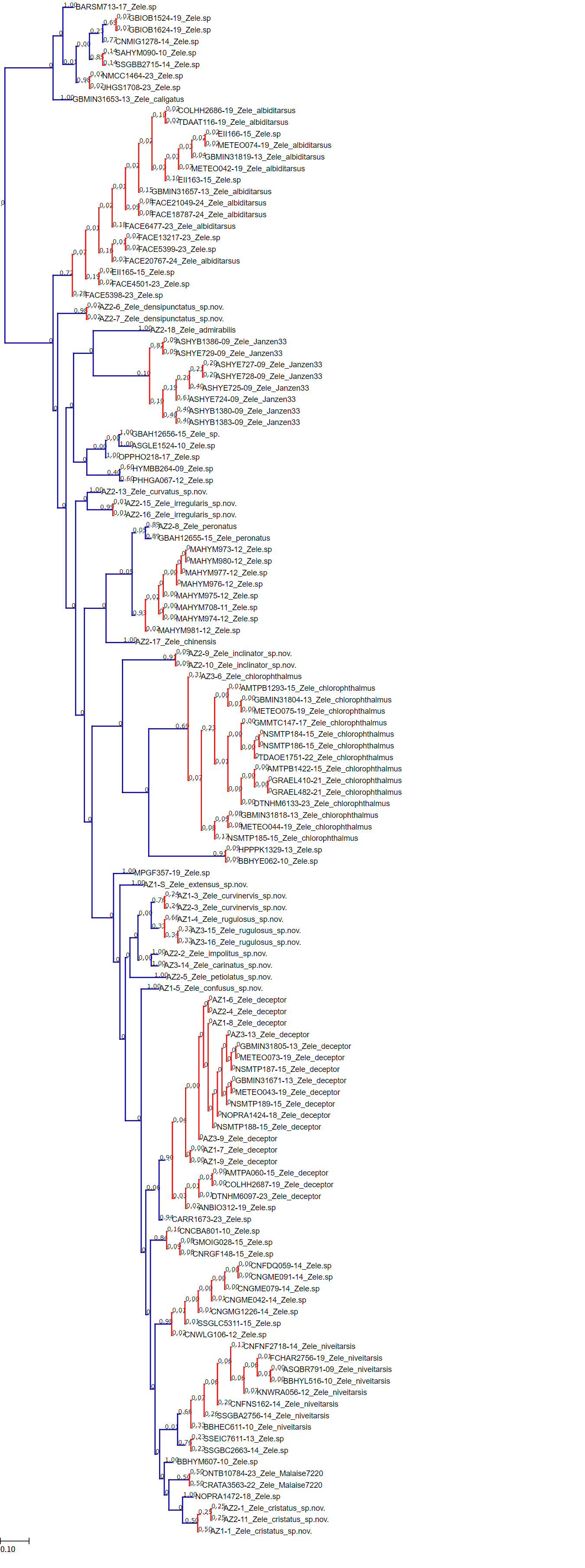

Supplement: Supplementary material 6 — PTP species delimitation results MLP artition [file zookeys-1248-125_article-158182__-s006.png]

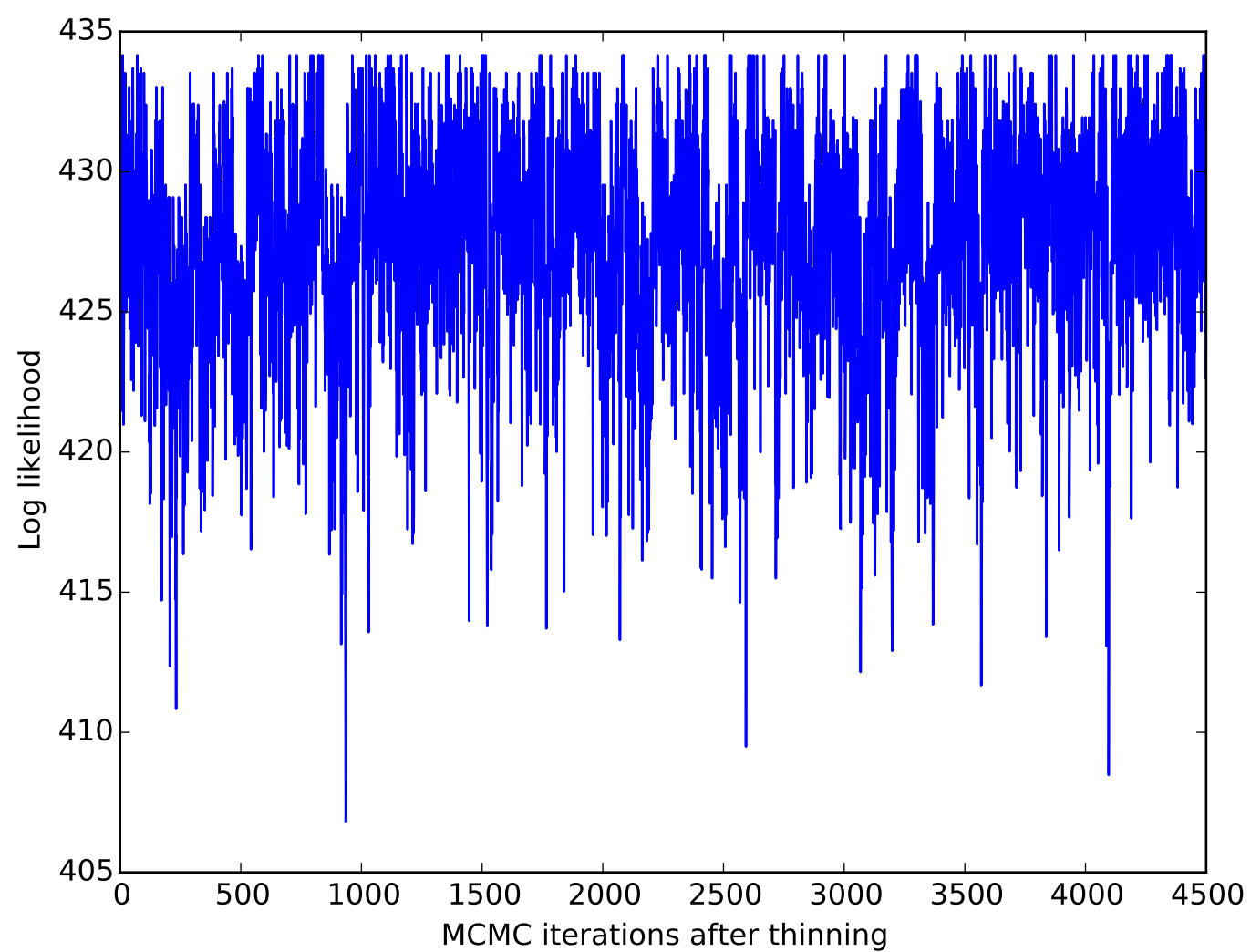

Supplement: Supplementary material 7 — PTP species delimitation results likelihood trace plot [file zookeys-1248-125_article-158182__-s007.pdf]
